# Supplementary material for: CodonTransformer: a multispecies codon optimizer using context-aware neural networks
Source: Nat Commun. 2025 Apr 3;16:3205. doi: 10.1038/s41467-025-58588-7 (PMC11968976; doi:10.1038/s41467-025-58588-7)
Supplement: Supplementary file 1 — Supplementary Information [file 41467_2025_58588_MOESM1_ESM.pdf]

Supplementary information for

CodonTransformer: a multispecies codon optimizer using context-aware neural networks

Adibvafa Fallahpour[1,2*], Vincent Gureghian[3,4*], Guillaume J. Filion[2‡], Ariel B. Lindner[3,4,5‡], Amir Pandi[3,4,5‡]

[1] Vector Institute for Artificial Intelligence, Toronto ON, Canada
[2] University of Toronto Scarborough; Department of Biological Science; Scarborough ON, Canada
[3] Sorbonne Université, CNRS, ERL U1338 Inserm, Department of Computational, Quantitative and Synthetic Biology, F-75005 Paris, France
[4] Sorbonne Université, CNRS, Inserm, Institut de Biologie Paris-Seine, F-75005 Paris, France
[5] Sorbonne Université, CNRS, Université de Technologie de Compiègne, Inserm, Biofoundry Alliance Sorbonne Université, F-75005 Paris, France

[*] These authors contributed equally to this work.

[‡] To whom correspondence should be addressed:
guillaume.filion@utoronto.ca, ariel.lindner@inserm.fr, amir.pandi@inserm.fr

This supplementary information contains:

Supplementary Fig. 1: Kernel density plots for DNA similarity.
Supplementary Fig. 2: CSI, GC content, and CFD of genomic and generated DNA sequences for *E. coli*.
Supplementary Fig. 3: CSI, GC content, and CFD of genomic and generated DNA sequences for *B. subtilis*.
Supplementary Fig. 4: CSI, GC content, and CFD of genomic and generated DNA sequences for *P. putida*.
Supplementary Fig. 5: CSI, GC content, and CFD of genomic and generated DNA sequences for *T. barophilus*.
Supplementary Fig. 6: CSI, GC content, and CFD of genomic and generated DNA sequences for *S. cerevisiae*.
Supplementary Fig. 7: CSI, GC content, and CFD of genomic and generated DNA sequences for *C. reinhardtii*.
Supplementary Fig. 8: CSI, GC content, and CFD of genomic and generated DNA sequences for *C. reinhardtii* chloroplast.
Supplementary Fig. 9: CSI, GC content, and CFD of genomic and generated DNA sequences for *A. thaliana*.
Supplementary Fig. 10: CSI, GC content, and CFD of genomic and generated DNA sequences for *N. tabacum*.
Supplementary Fig. 11: CSI, GC content, and CFD of genomic and generated DNA sequences for *N. tabacum* chloroplast.
Supplementary Fig. 12: CSI, GC content, and CFD of genomic and generated DNA sequences for *C. elegans*.
Supplementary Fig. 13: CSI, GC content, and CFD of genomic and generated DNA sequences for *D. rerio*.
Supplementary Fig. 14: CSI, GC content, and CFD of genomic and generated DNA sequences for *D. melanogaster*.
Supplementary Fig. 15: CSI, GC content, and CFD of genomic and generated DNA sequences for *M. musculus*.
Supplementary Fig. 16: CSI, GC content, and CFD of genomic and generated DNA sequences for *H. sapiens*.
Supplementary Fig. 17: CodonTransformer embedding for organisms used for training.
Supplementary Fig. 18: Model comparison based on normalized DTW distances for subsets of natural genes.
Supplementary Fig. 19: Model comparison based on the minimum free energy of RNA folding.
Supplementary Fig. 20: Model comparison based on Jaccard index.
Supplementary Fig. 21: Model comparison based on sequence similarity.
Supplementary Fig. 22: Model comparison based on the distribution of 64 codons for benchmark proteins.
Supplementary Fig. 23: Model comparison based on the distribution of 64 codons for subsets of endogenous proteins.
Supplementary Fig. 24 Distribution of 64 codons among genomes in natural genes and optimized counterparts by Base and Fine-tuned CodonTransformer.
Supplementary Fig. 25: Model comparison based on normalized DTW distances for benchmark proteins.
Supplementary Fig. 26: The average number of negative cis-regulatory elements in subsets of E. coli genes.

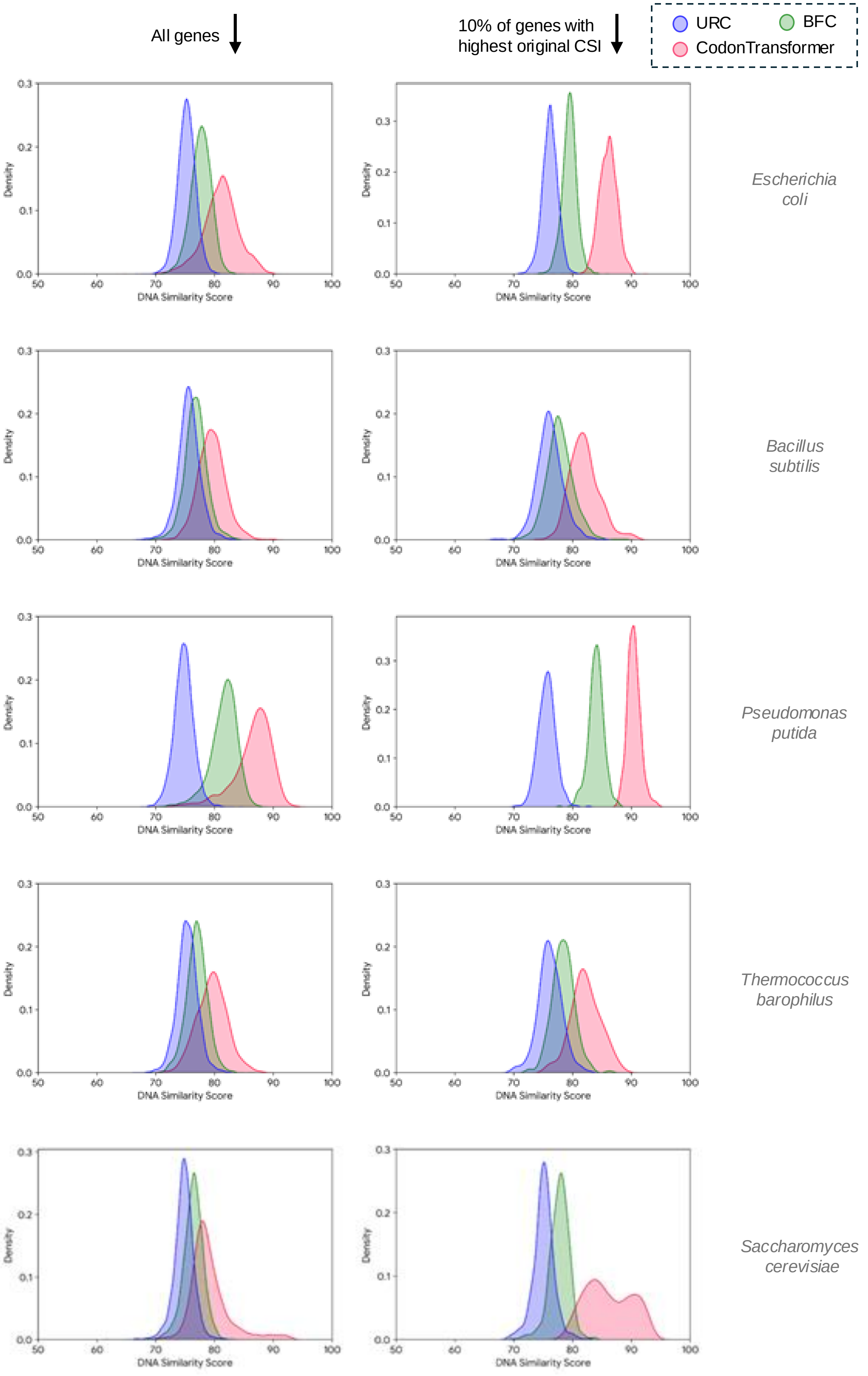

Supplementary Fig. 1, continued on the next page.

Continued Supplementary Fig. 1

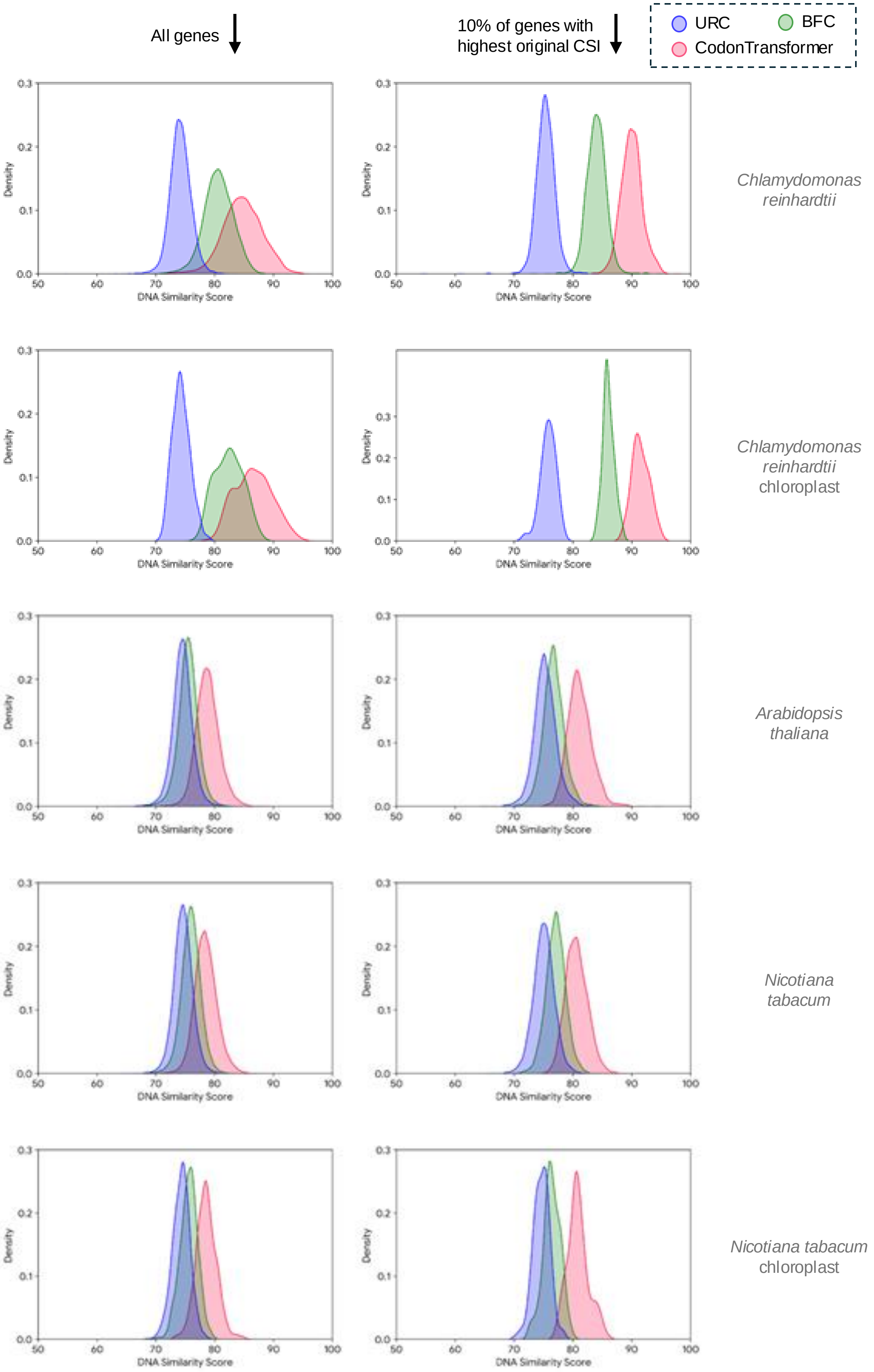

Supplementary Fig. 1, continued on the next page.

Continued Supplementary Fig. 1

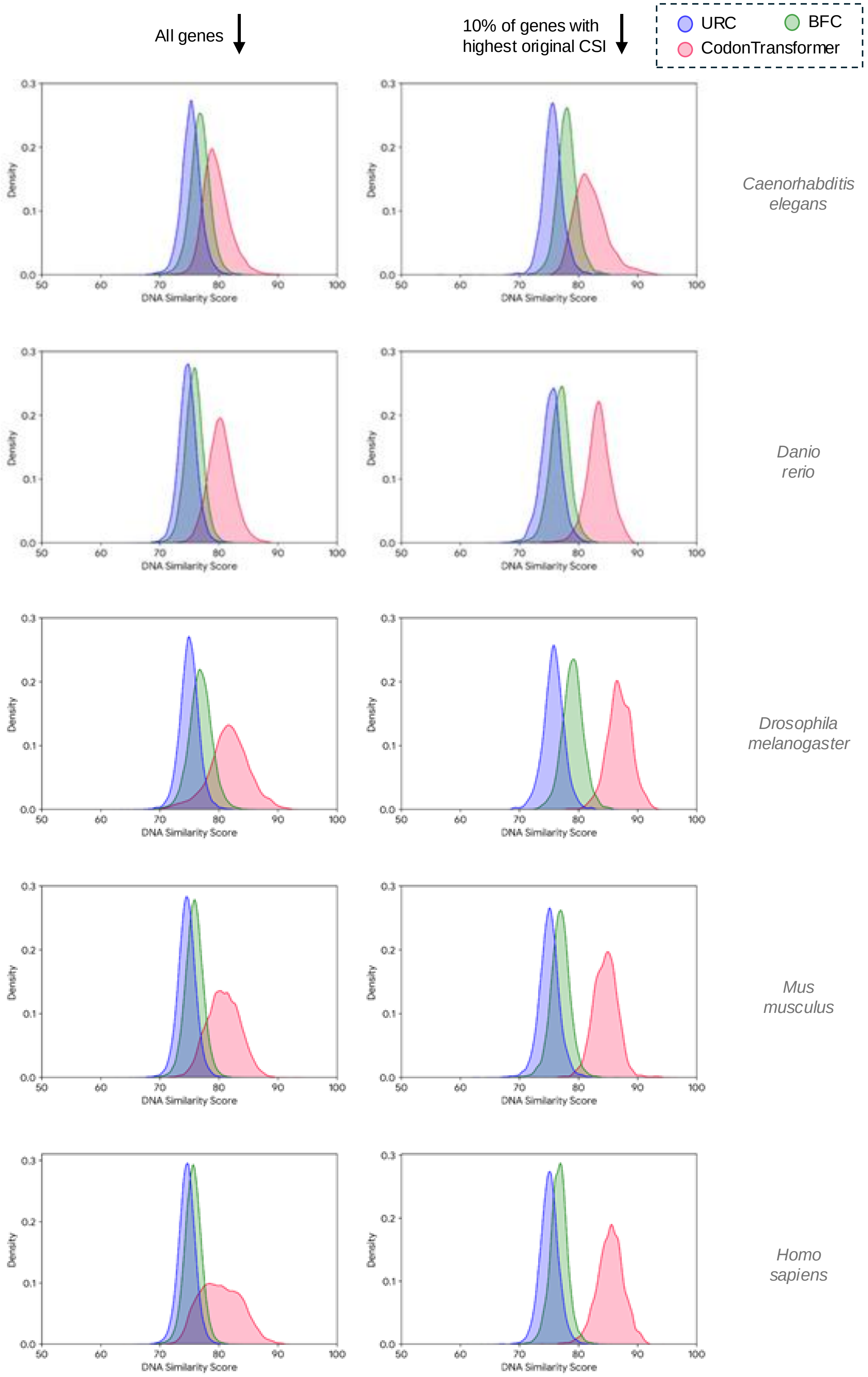

Supplementary Fig. 1: Kernel density plots for DNA similarity between original genes and DNA sequences designed by CodonTransformer (red), codons with Unified Random Choice (URC, blue) and Background Frequency Choice (BFC, green). The left plots are for all genes for each organism and right plots are for 10% genes with the highest original CSI).

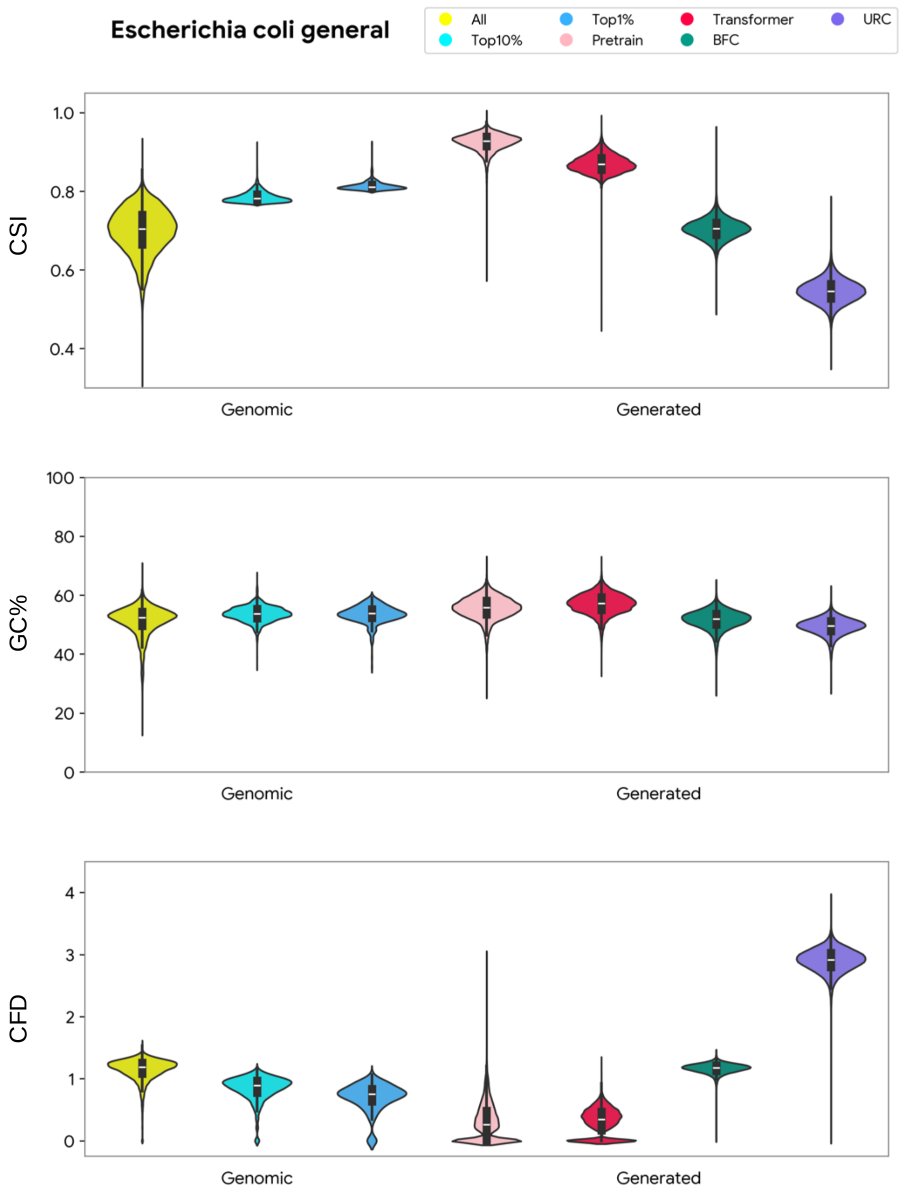

Supplementary Fig. 2: Codon similarity index (CSI), GC content, and codon frequency distribution (CFD) of genomic DNA sequences of *E. coli* general (merged *E. coli* genomes) and their generated counterparts by CodonTransformer, base (Pretrain) and fine-tuned (Transformer), BFC (Background Frequency Choice) and URC (Unified Random Choice). Source data for this figure is available at https://zenodo.org/records/13262517.

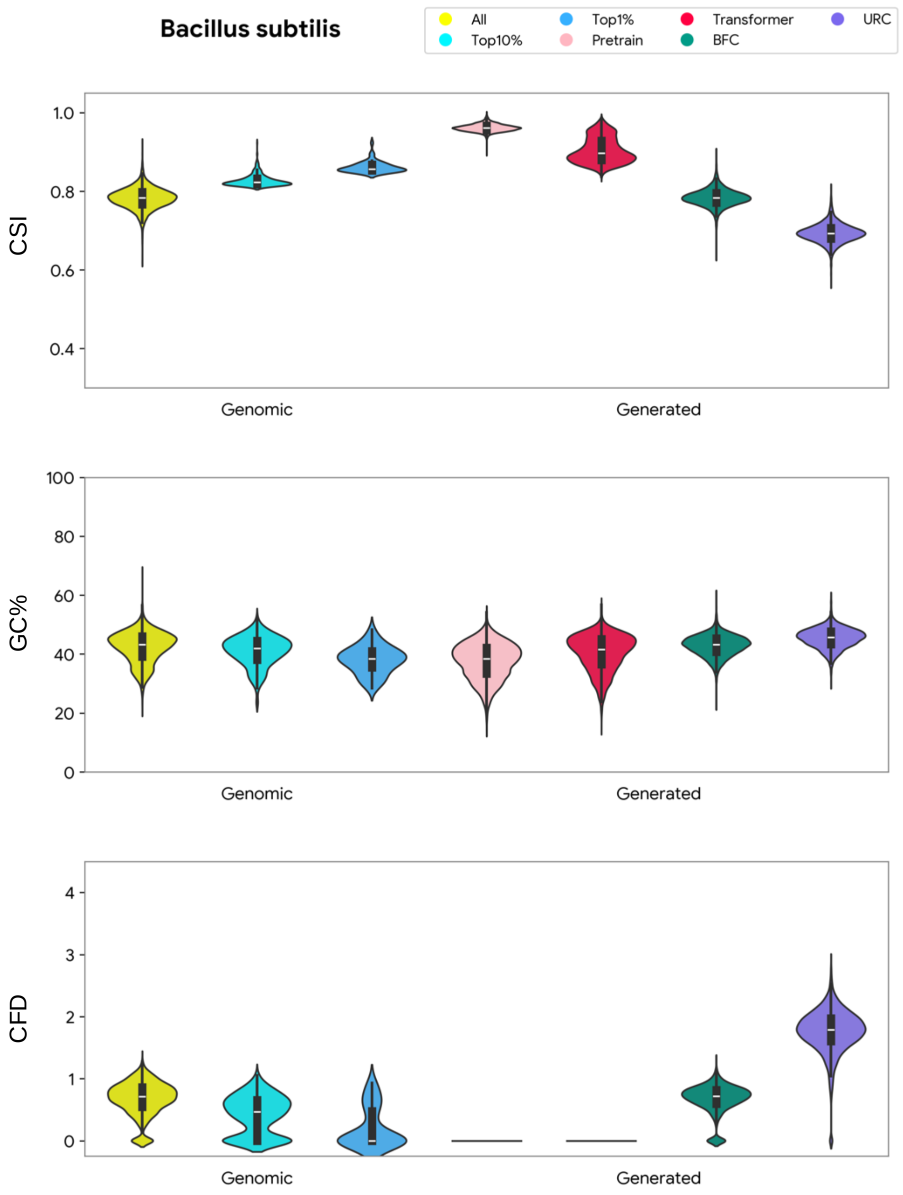

Supplementary Fig. 3: Codon similarity index (CSI), GC content, and codon frequency distribution (CFD) for genomic DNA sequences of *B. subtilis* and their generated counterparts by CodonTransformer base (Pretrain) and fine-tuned (Transformer), BFC (Background Frequency Choice) and URC (Unified Random Choice). Source data for this figure is available at https://zenodo.org/records/13262517.

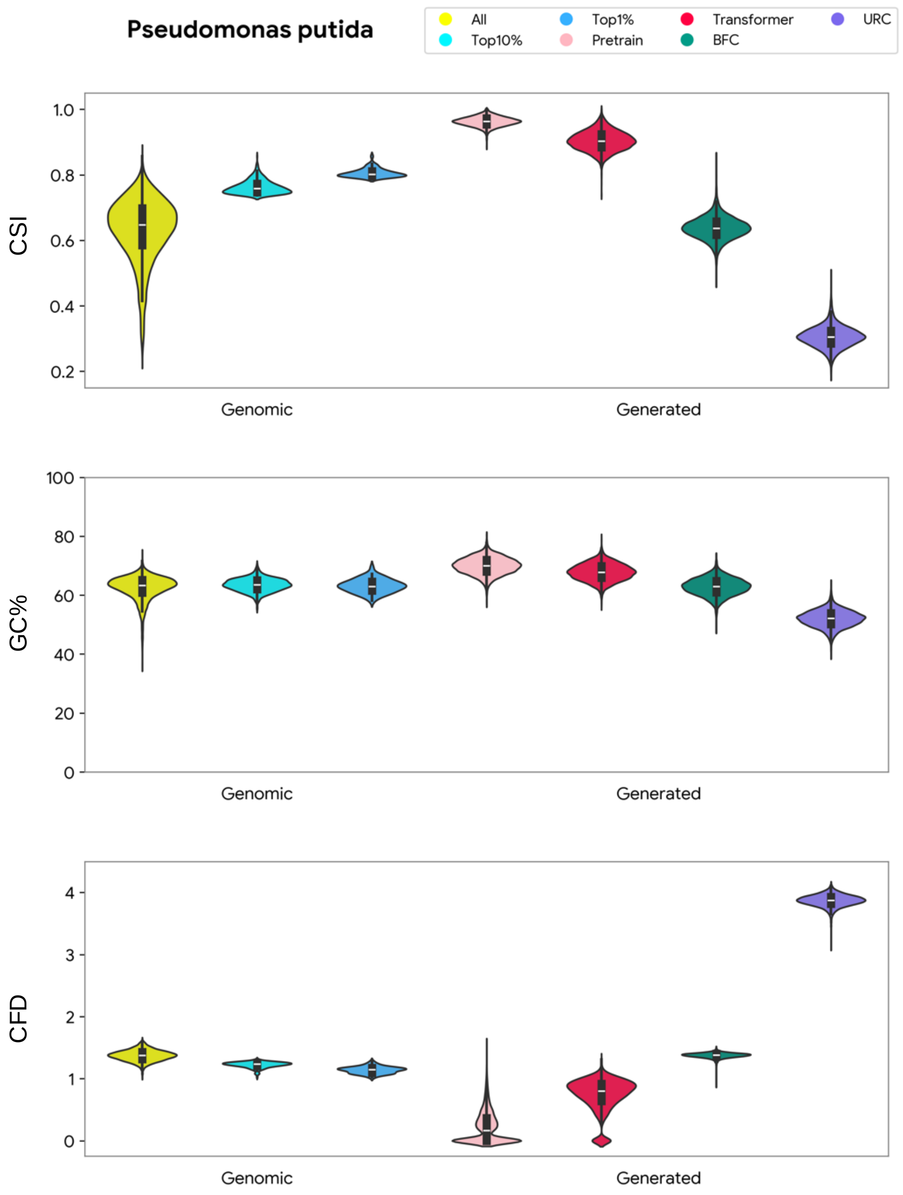

Supplementary Fig. 4: Codon similarity index (CSI), GC content, and codon frequency distribution (CFD) for genomic DNA sequences of *P. putida* and their generated counterparts by CodonTransformer base (Pretrain) and fine-tuned (Transformer), BFC (Background Frequency Choice) and URC (Unified Random Choice). Source data for this figure is available at https://zenodo.org/records/13262517.

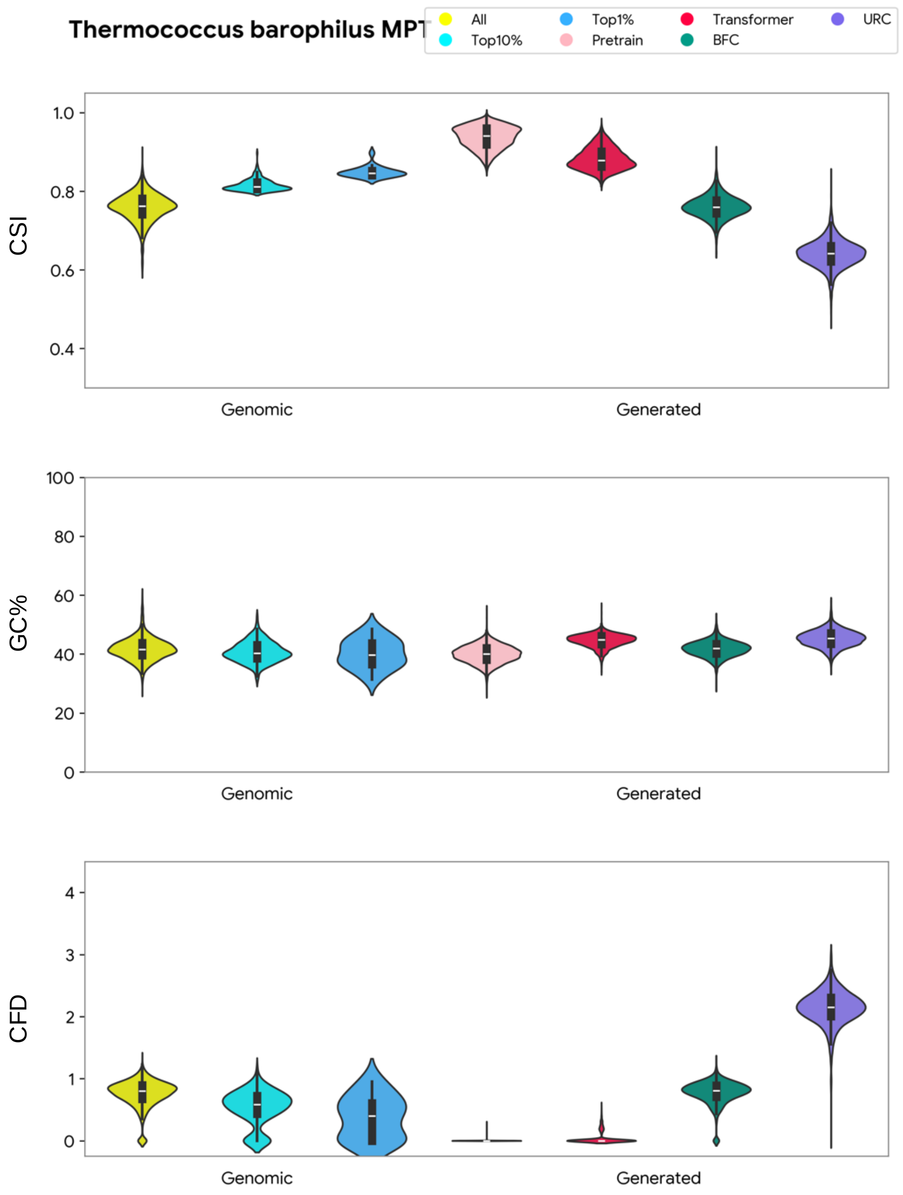

Supplementary Fig. 5: Codon similarity index (CSI), GC content, and codon frequency distribution (CFD) for genomic DNA sequences of *T. barophilus* and their generated counterparts by CodonTransformer base (Pretrain) and fine-tuned (Transformer), BFC (Background Frequency Choice) and URC (Unified Random Choice). Source data for this figure is available at https://zenodo.org/records/13262517.

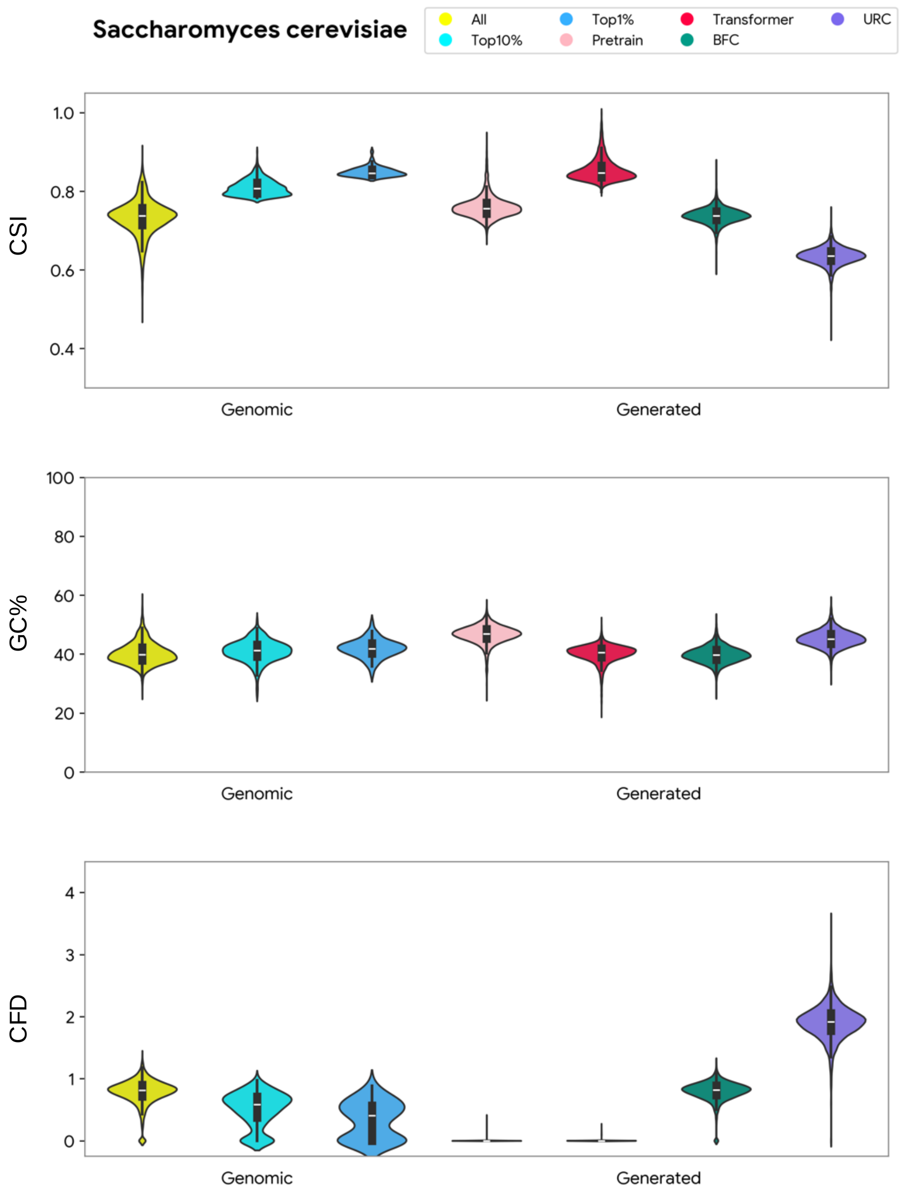

Supplementary Fig. 6: Codon similarity index (CSI), GC content, and codon frequency distribution (CFD) for genomic DNA sequences of *S. cerevisiae* and their generated counterparts by CodonTransformer base (Pretrain) and fine-tuned (Transformer), BFC (Background Frequency Choice) and URC (Unified Random Choice). Source data for this figure is available at https://zenodo.org/records/13262517.

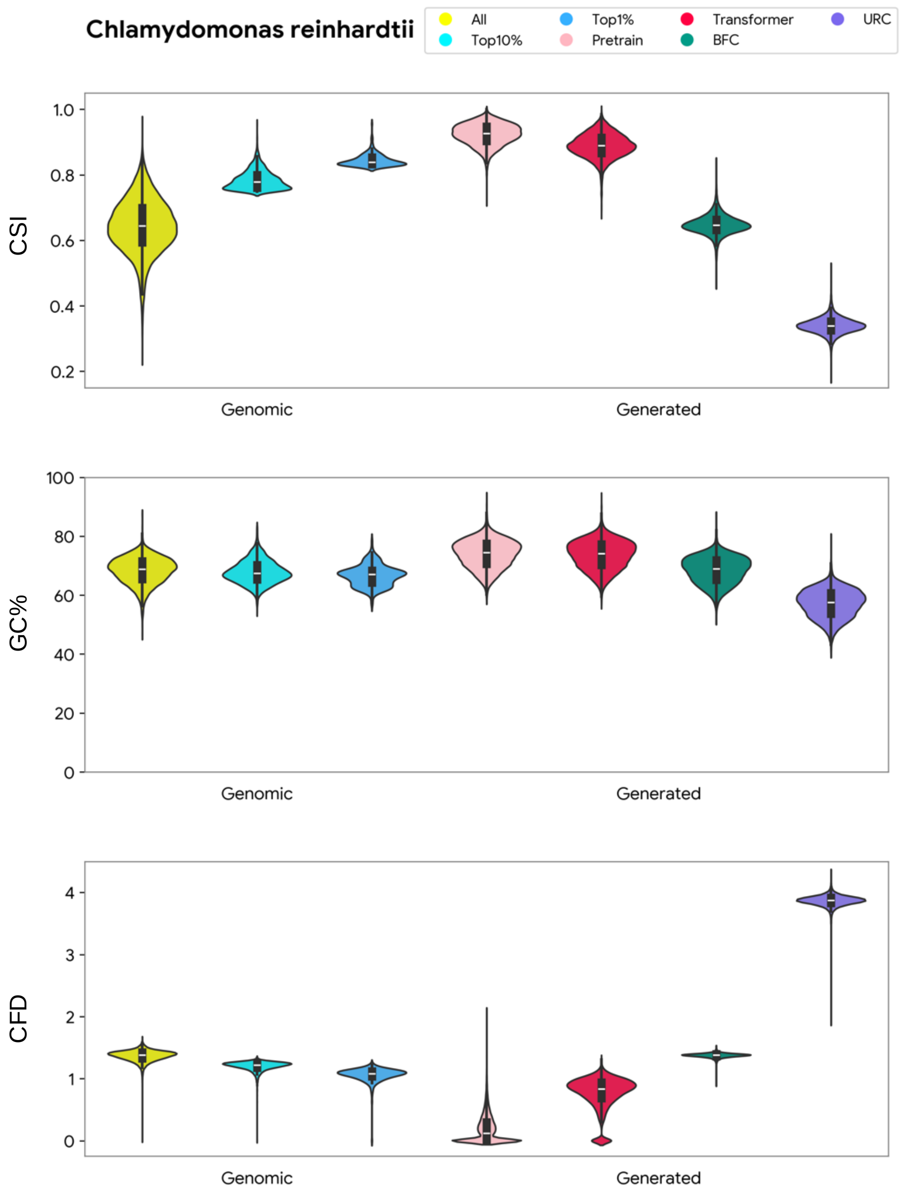

Supplementary Fig. 7: Codon similarity index (CSI), GC content, and codon frequency distribution (CFD) for genomic DNA sequences of *C. reinhardtii* and their generated counterparts by CodonTransformer base (Pretrain) and fine-tuned (Transformer), BFC (Background Frequency Choice) and URC (Unified Random Choice). Source data for this figure is available at https://zenodo.org/records/13262517.

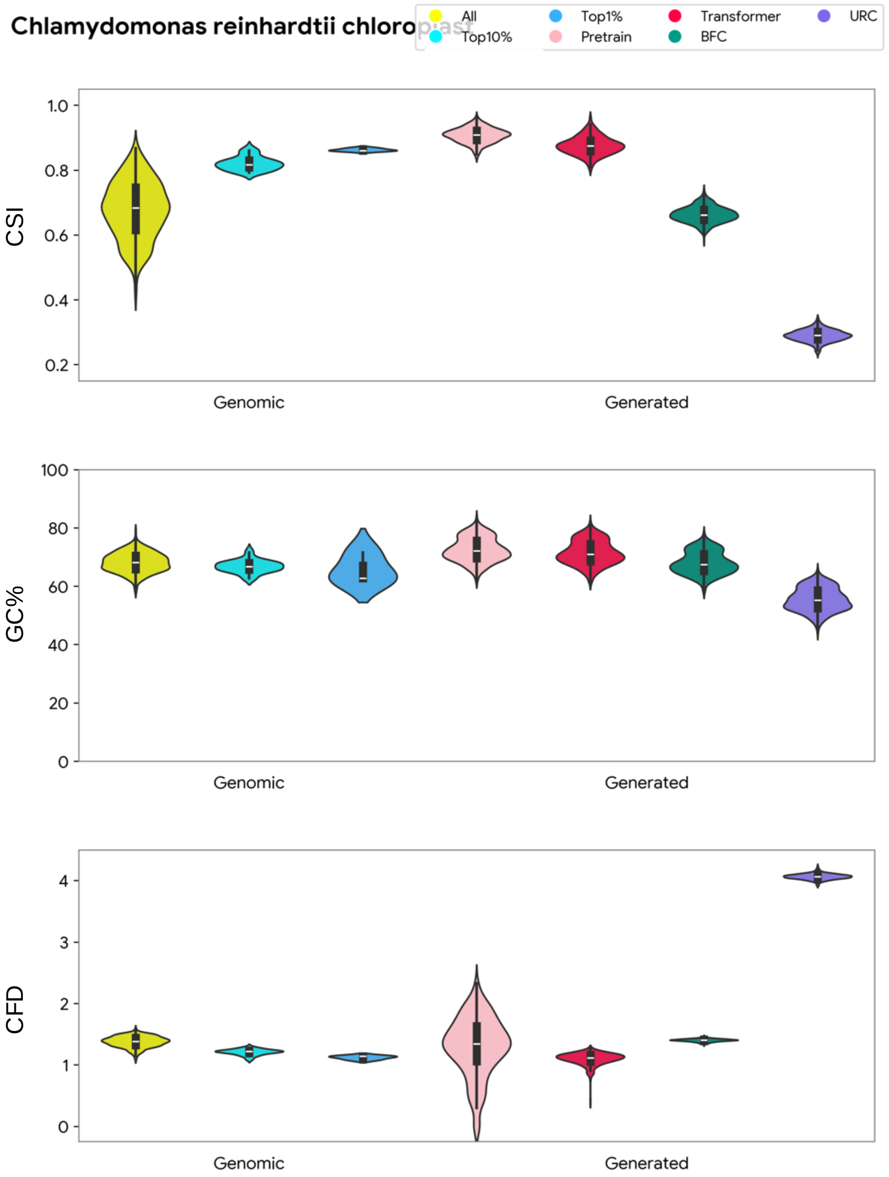

Supplementary Fig. 8: Codon similarity index (CSI), GC content, and codon frequency distribution (CFD) for genomic DNA sequences of *C. reinhardtii* chloroplast and their generated counterparts by CodonTransformer base (Pretrain) and fine-tuned (Transformer), BFC (Background Frequency Choice) and URC (Unified Random Choice). Source data for this figure is available at https://zenodo.org/records/13262517.

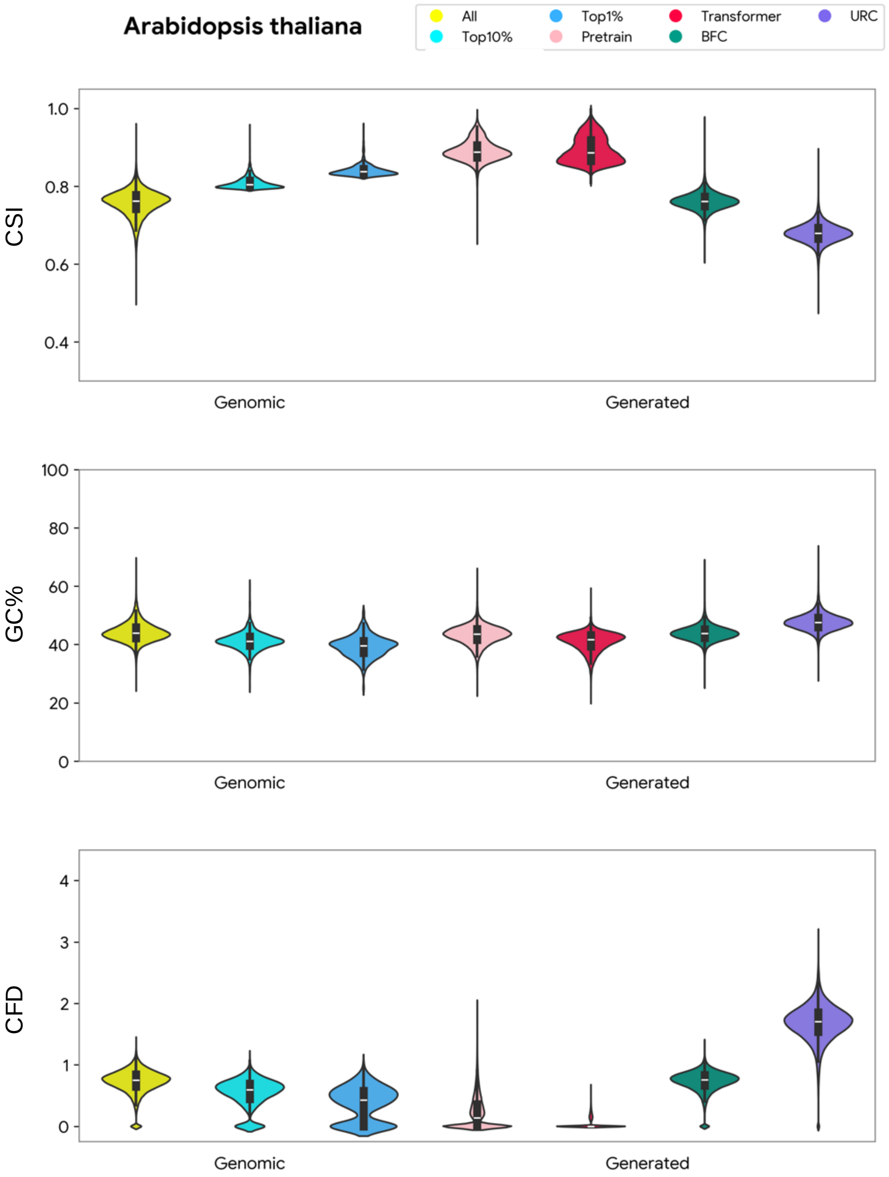

Supplementary Fig. 9: Codon similarity index (CSI), GC content, and codon frequency distribution (CFD) for genomic DNA sequences of *A. thaliana* and their generated counterparts by CodonTransformer base (Pretrain) and fine-tuned (Transformer), BFC (Background Frequency Choice) and URC (Unified Random Choice). Source data for this figure is available at https://zenodo.org/records/13262517.

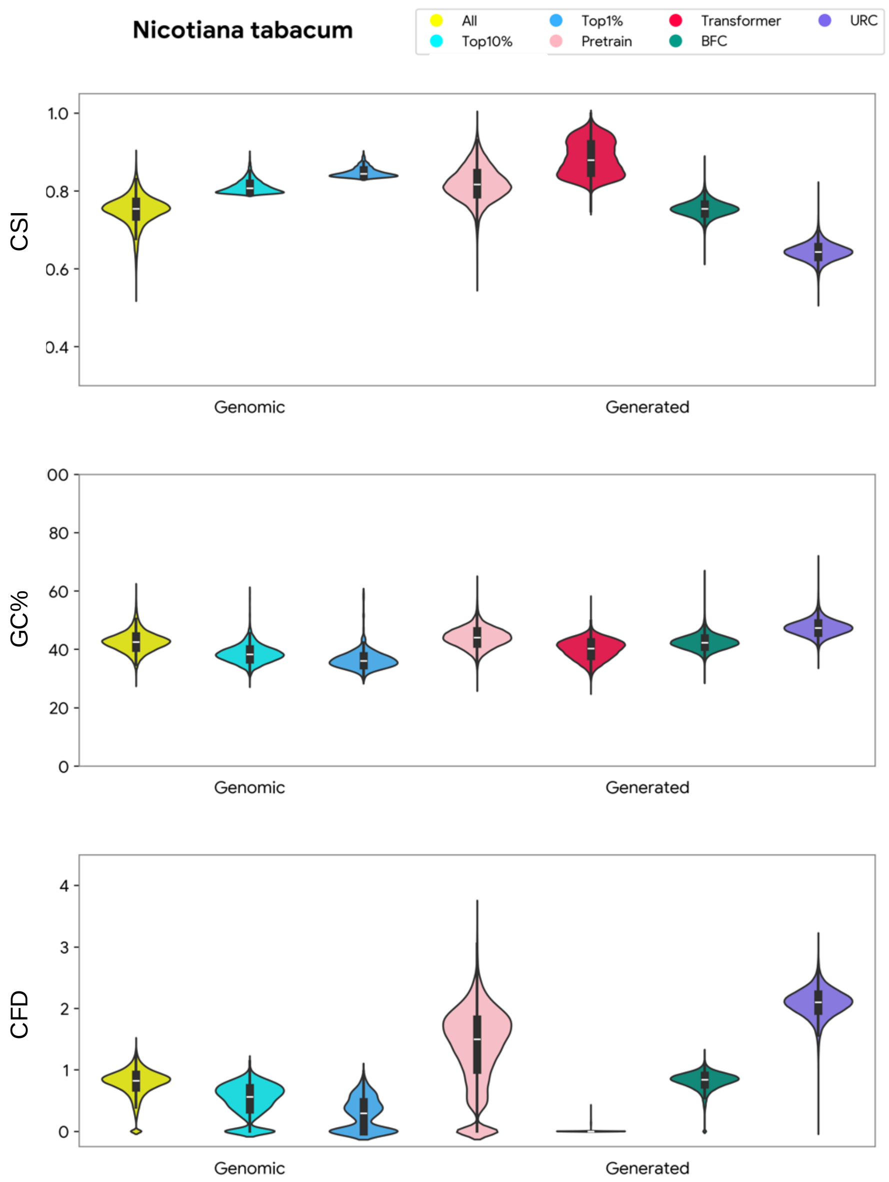

Supplementary Fig. 10: Codon similarity index (CSI), GC content, and codon frequency distribution (CFD) for genomic DNA sequences of *N. tabacum* and their generated counterparts by CodonTransformer base (Pretrain) and fine-tuned (Transformer), BFC (Background Frequency Choice) and URC (Unified Random Choice). Source data for this figure is available at https://zenodo.org/records/13262517.

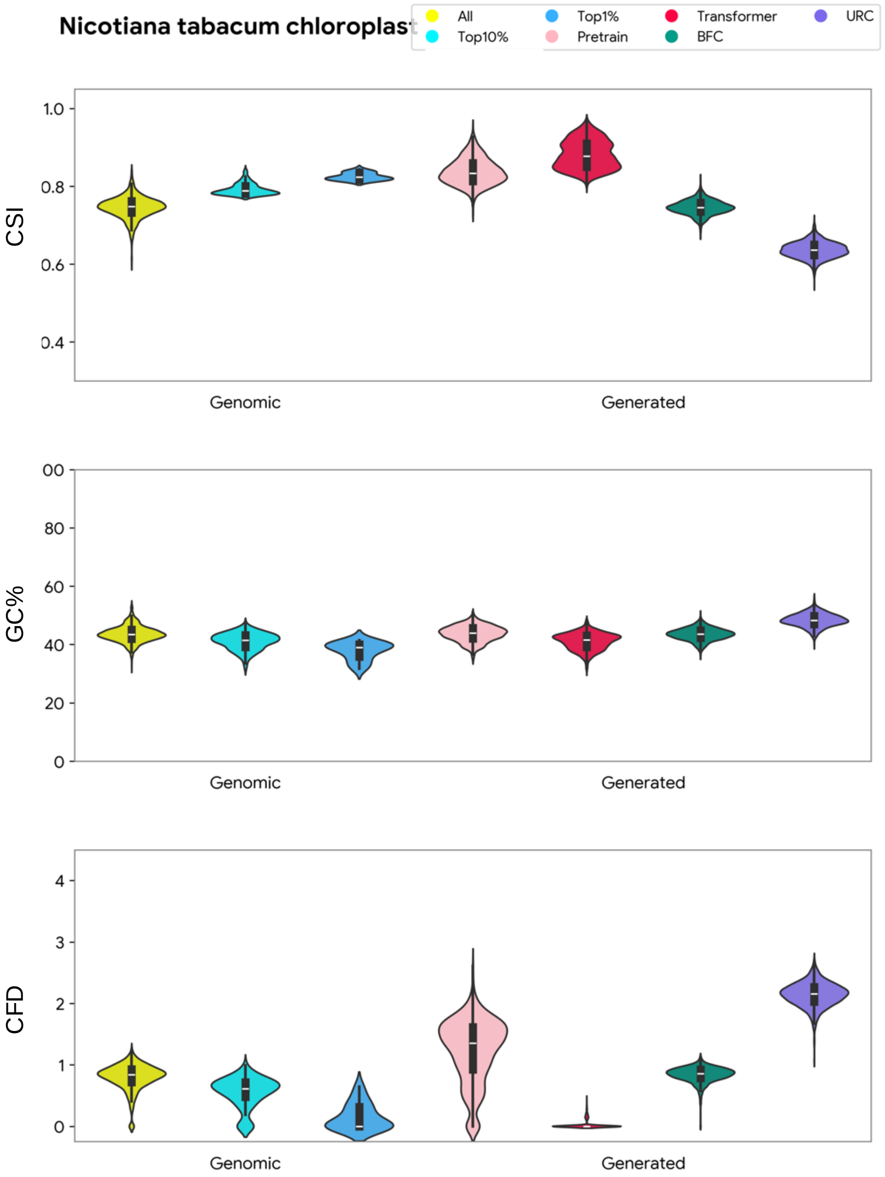

Supplementary Fig. 11: Codon similarity index (CSI), GC content, and codon frequency distribution (CFD) for genomic DNA sequences of *N. tabacum* chloroplast and their generated counterparts by CodonTransformer base (Pretrain) and fine-tuned (Transformer), BFC (Background Frequency Choice) and URC (Unified Random Choice). Source data for this figure is available at https://zenodo.org/records/13262517.

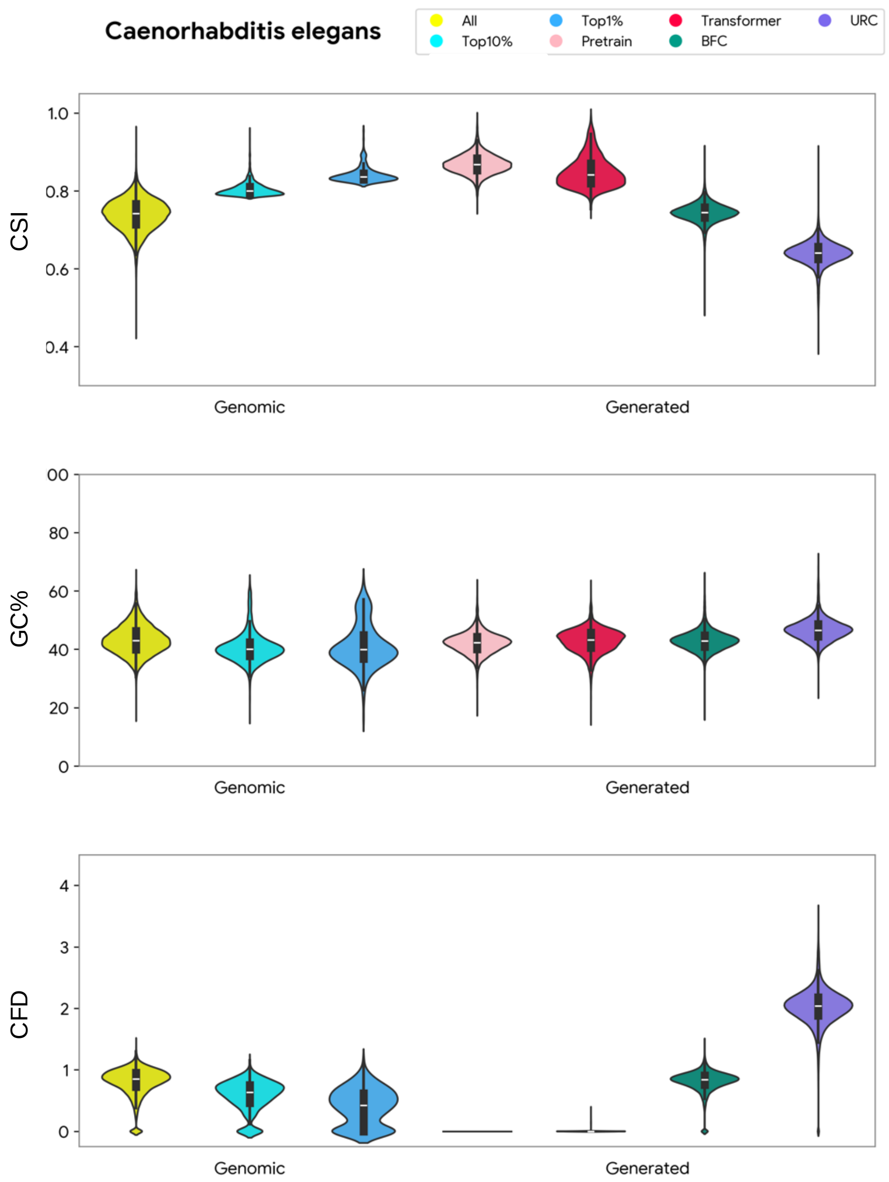

Supplementary Fig. 12: Codon similarity index (CSI), GC content, and codon frequency distribution (CFD) for genomic DNA sequences of *C. elegans* and their generated counterparts by CodonTransformer base (Pretrain) and fine-tuned (Transformer), BFC (Background Frequency Choice) and URC (Unified Random Choice). Source data for this figure is available at https://zenodo.org/records/13262517.

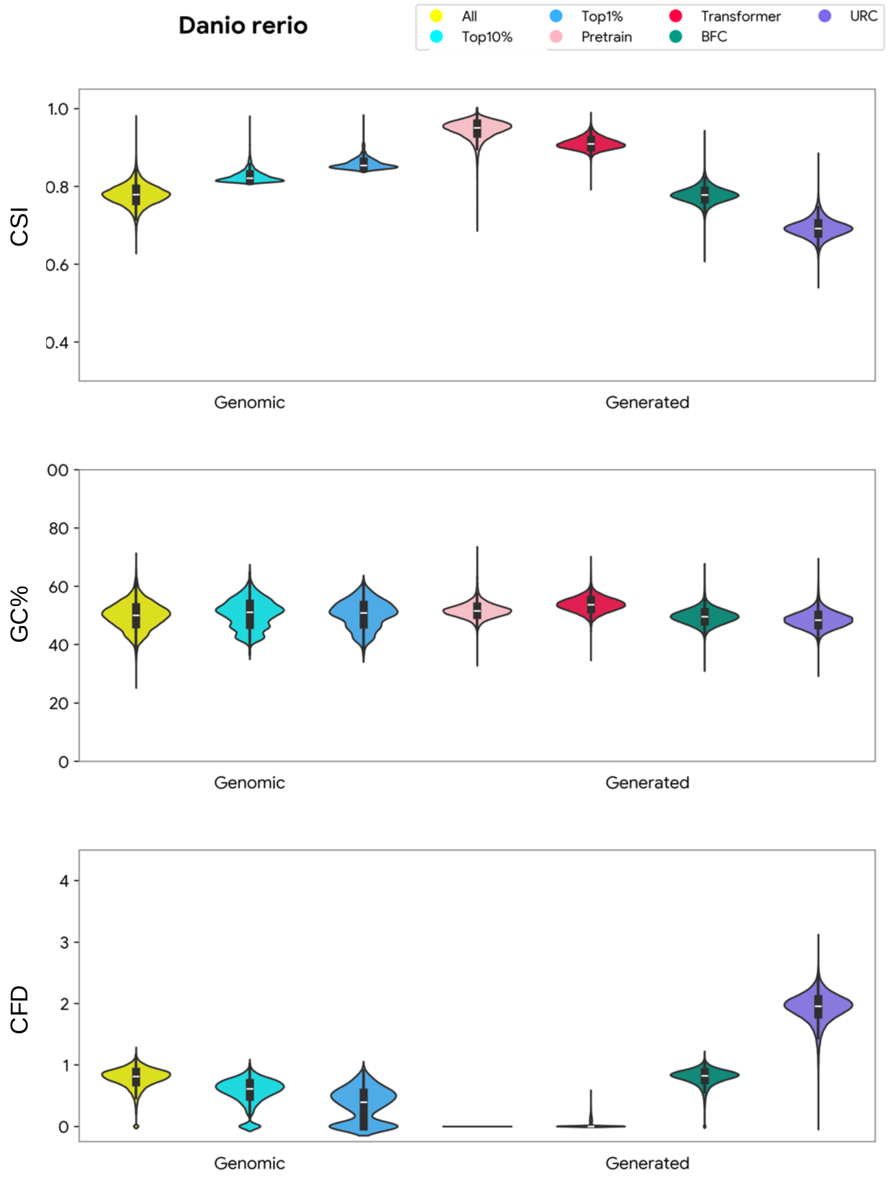

Supplementary Fig. 13: Codon similarity index (CSI), GC content, and codon frequency distribution (CFD) for genomic DNA sequences of *D. rerio* and their generated counterparts by CodonTransformer base (Pretrain) and fine-tuned (Transformer), BFC (Background Frequency Choice) and URC (Unified Random Choice). Source data for this figure is available at https://zenodo.org/records/13262517.

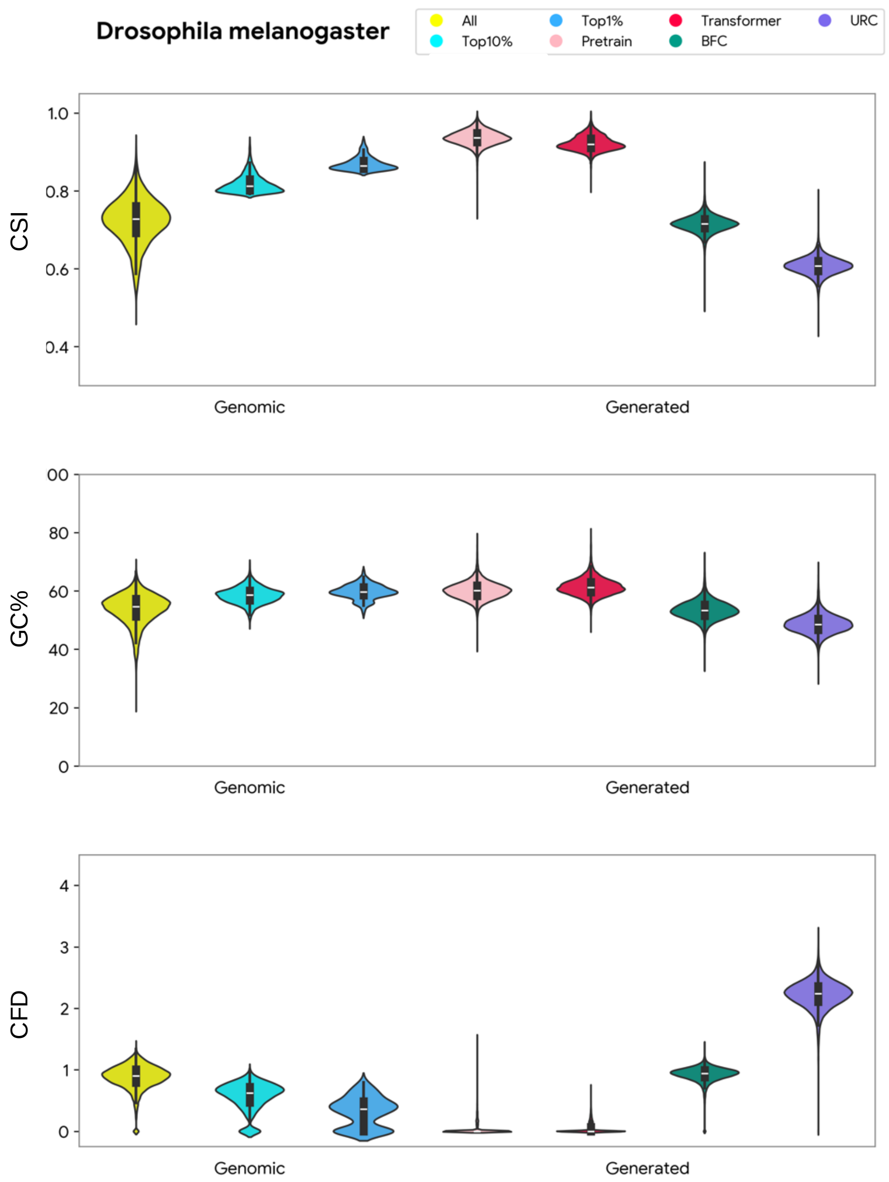

Supplementary Fig. 14: Codon similarity index (CSI), GC content, and codon frequency distribution (CFD) for genomic DNA sequences of *D. melanogaster* and their generated counterparts by CodonTransformer base (Pretrain) and fine-tuned (Transformer), BFC (Background Frequency Choice) and URC (Unified Random Choice). Source data for this figure is available at https://zenodo.org/records/13262517.

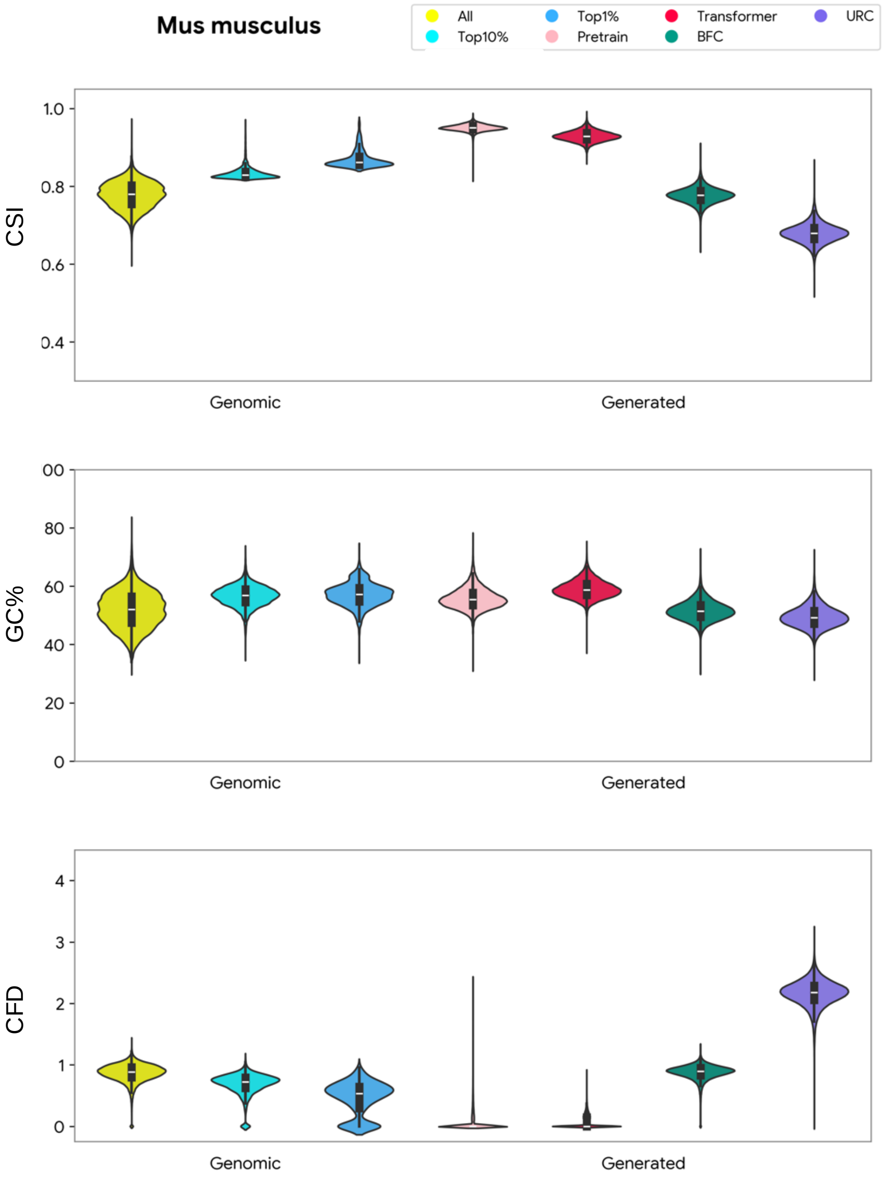

Supplementary Fig. 15: Codon similarity index (CSI), GC content, and codon frequency distribution (CFD) for genomic DNA sequences of *M. musculus* and their generated counterparts by CodonTransformer base (Pretrain) and fine-tuned (Transformer), BFC (Background Frequency Choice) and URC (Unified Random Choice). Source data for this figure is available at https://zenodo.org/records/13262517.

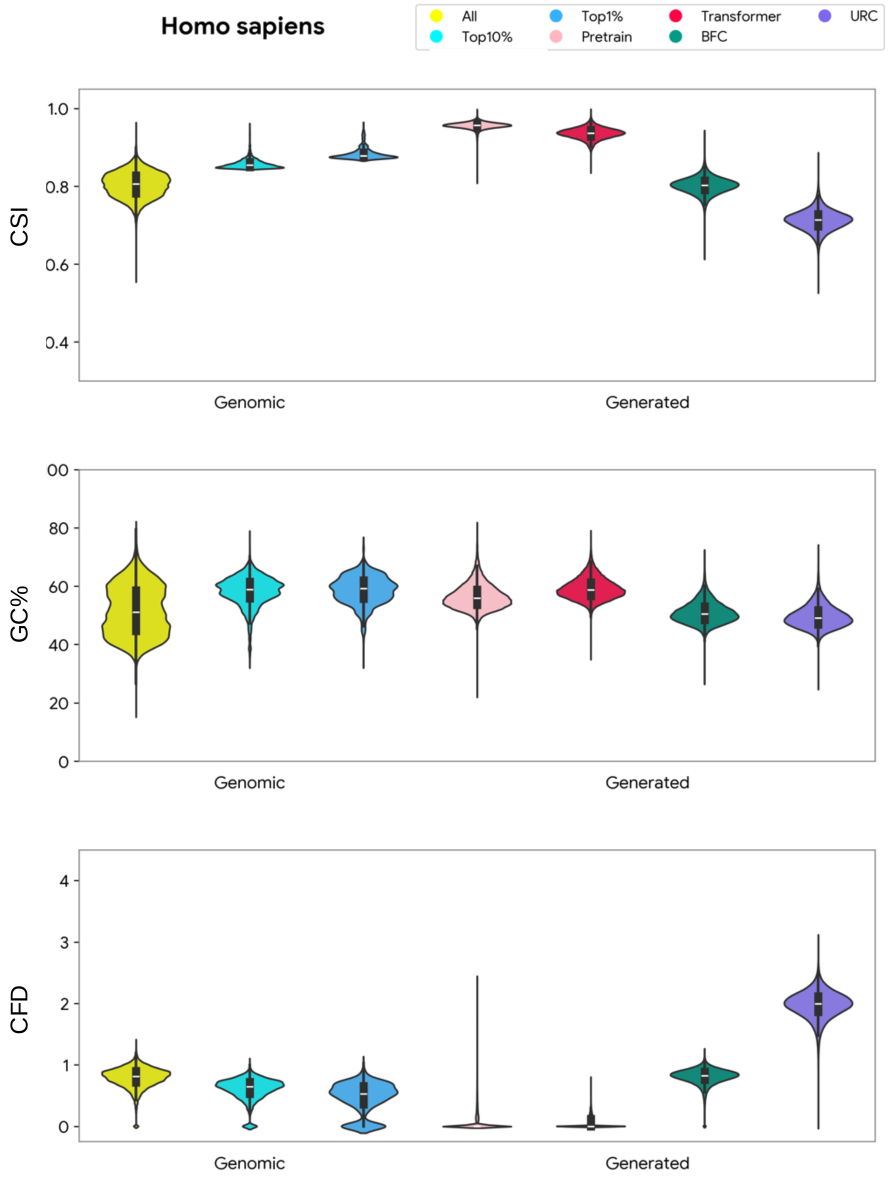

Supplementary Fig. 16: Codon similarity index (CSI), GC content, and codon frequency distribution (CFD) for genomic DNA sequences of *H. sapiens* and their generated counterparts by CodonTransformer base (Pretrain) and fine-tuned (Transformer), BFC (Background Frequency Choice) and URC (Unified Random Choice). Source data for this figure is available at https://zenodo.org/records/13262517.

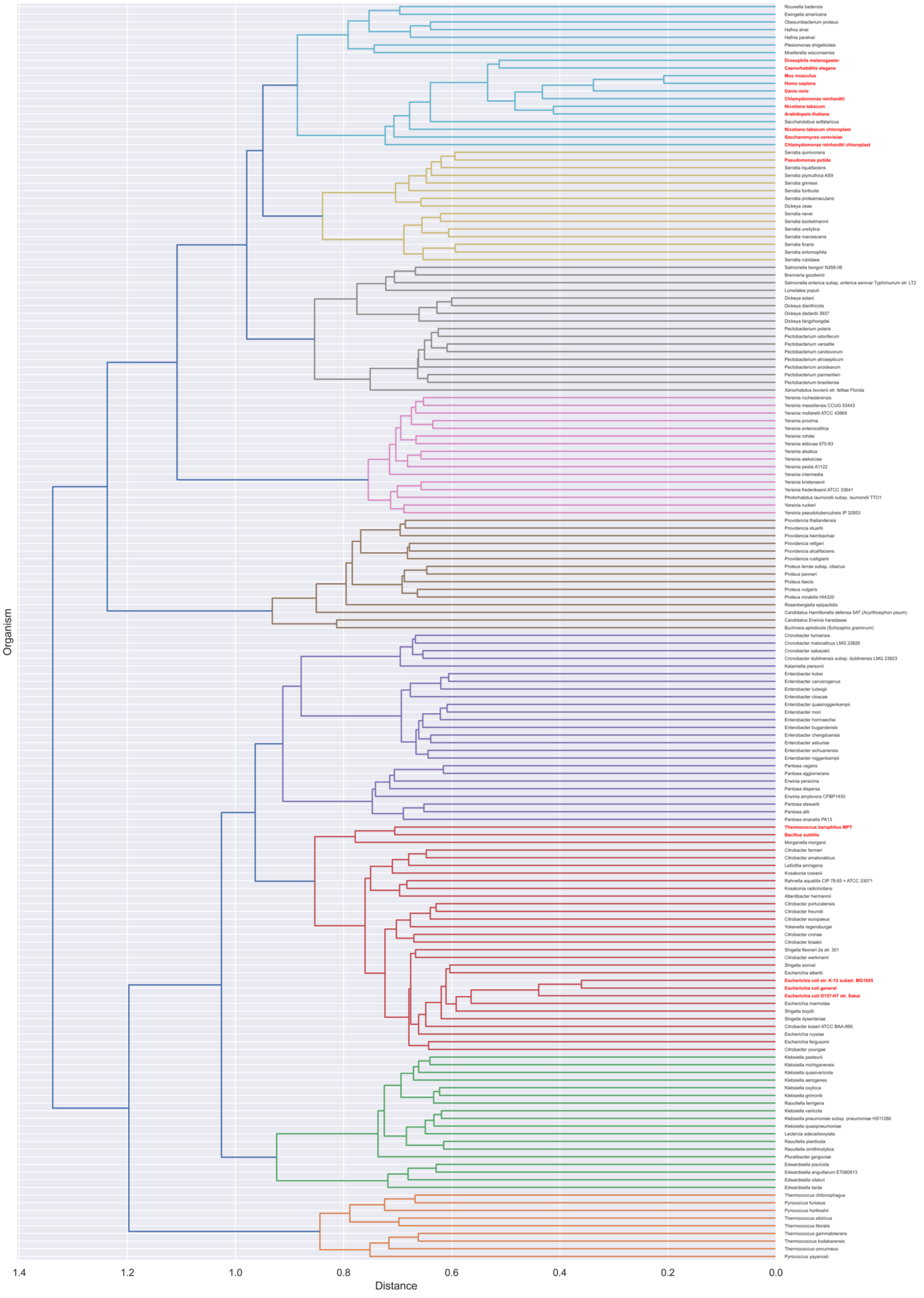

Supplementary Fig. 17: CodonTransformer embedding for organisms used for training (all, 164 species) and fine-tuning (red). These embeddings are plotted using hierarchical clustering which groups similar embeddings into clusters based on their variance, creating a hierarchical tree structure.

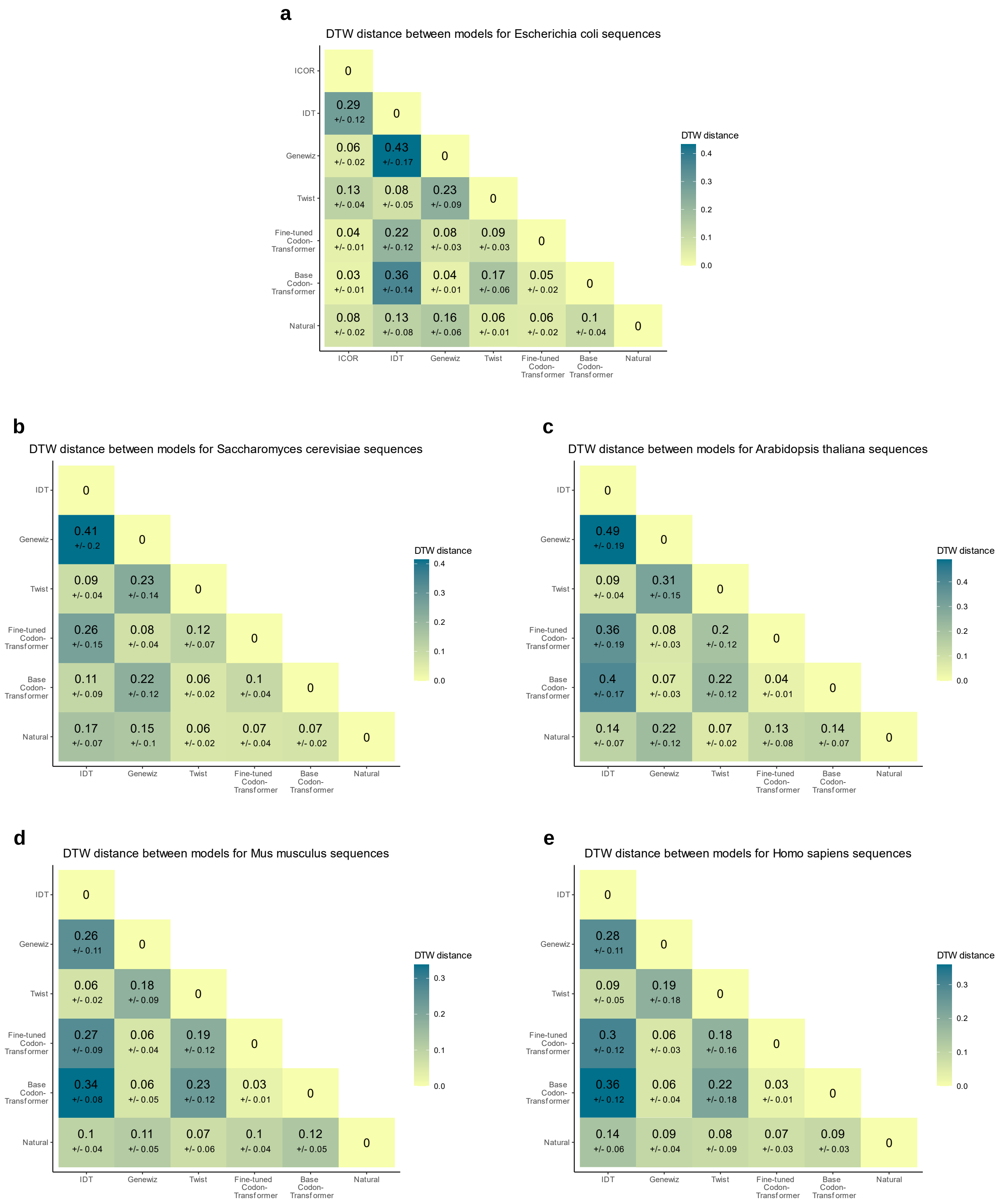

Supplementary Fig. 18: Model comparison based on normalized DTW distances between sequences generated for 50 random genes selected among top 10% CSI. Mean and standard deviation of normalized DTW distance between corresponding genes for *E. coli* (**a**), *S. cerevisiae* (**b**), *A. thaliana* (**c**), *M. musculus* (**d**), *H. sapiens* (**e**). Data underlying this figure is provided in the Source Data file.

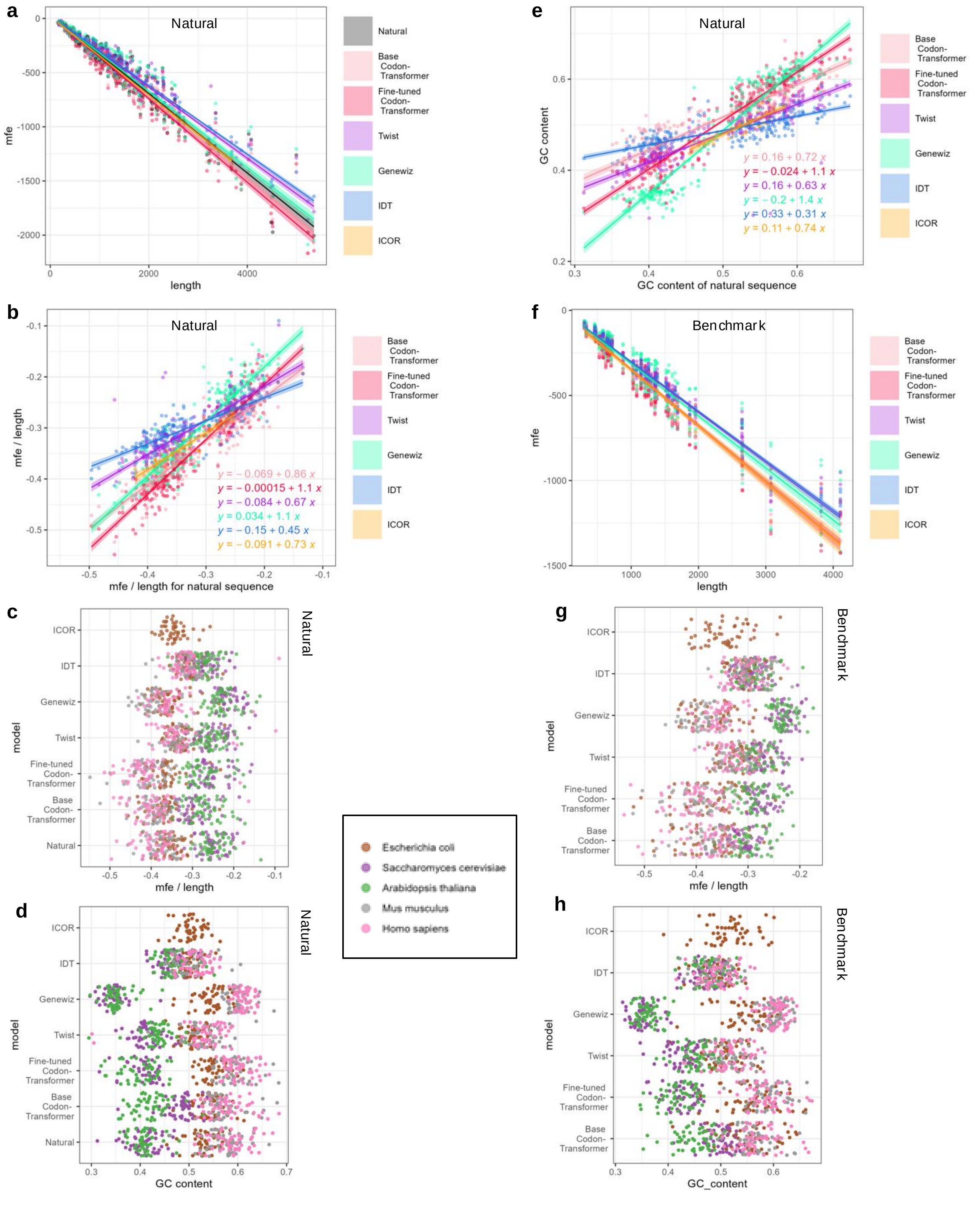

Supplementary Fig. 19: Model comparison based on minimum free energy (mfe) of RNA folding. Relationship between minimum folding energy and length for sequences generated by different models for 50 random genes among the top 10% CSI of each organism (**a**) and 52 benchmark proteins (**f**) for *E. coli, S. cerevisiae, A. thaliana, M. musculus, and H. sapiens*. **b**, Relationship between minimum folding energy normalized by protein length for generated and natural RNA. Minimum energy of RNA normalized by length for natural and benchmark proteins (**c** and **g**, respectively) and their GC content (**d** and **h**, respectively). **e**, Relationship between GC content of generated and natural. Data for this figure is provided in the Source Data file.

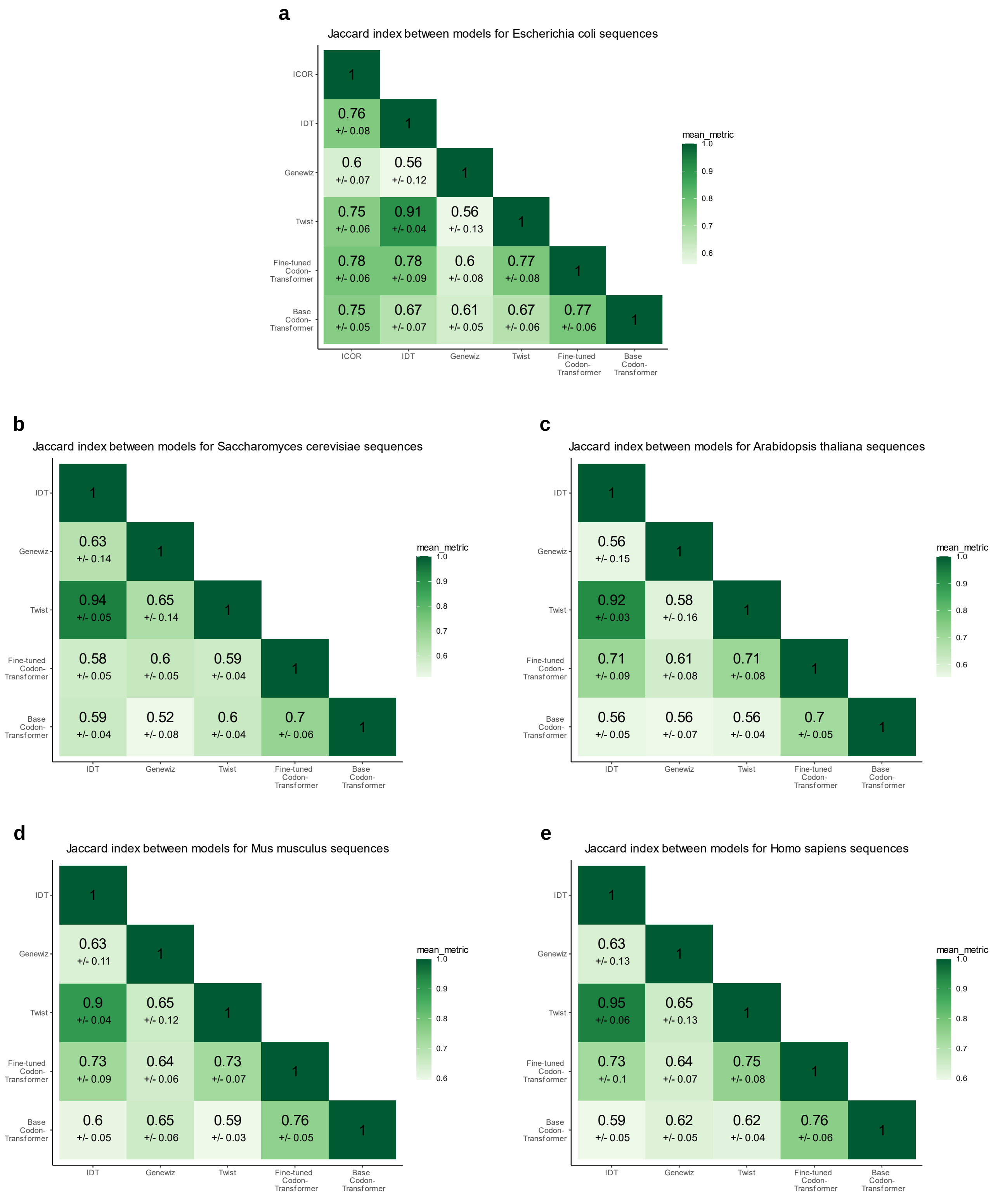

Supplementary Fig. 20: Model comparison based on Jaccard index between sequences generated for 52 benchmark proteins. Mean and standard deviation of Jaccard index between corresponding proteins for *E. coli* (**a**), *S. cerevisiae* (**b**), *A. thaliana* (**c**), *M. musculus* (**d**), *H. sapiens* (**e**). Data underlying this figure is provided in the Source Data file.

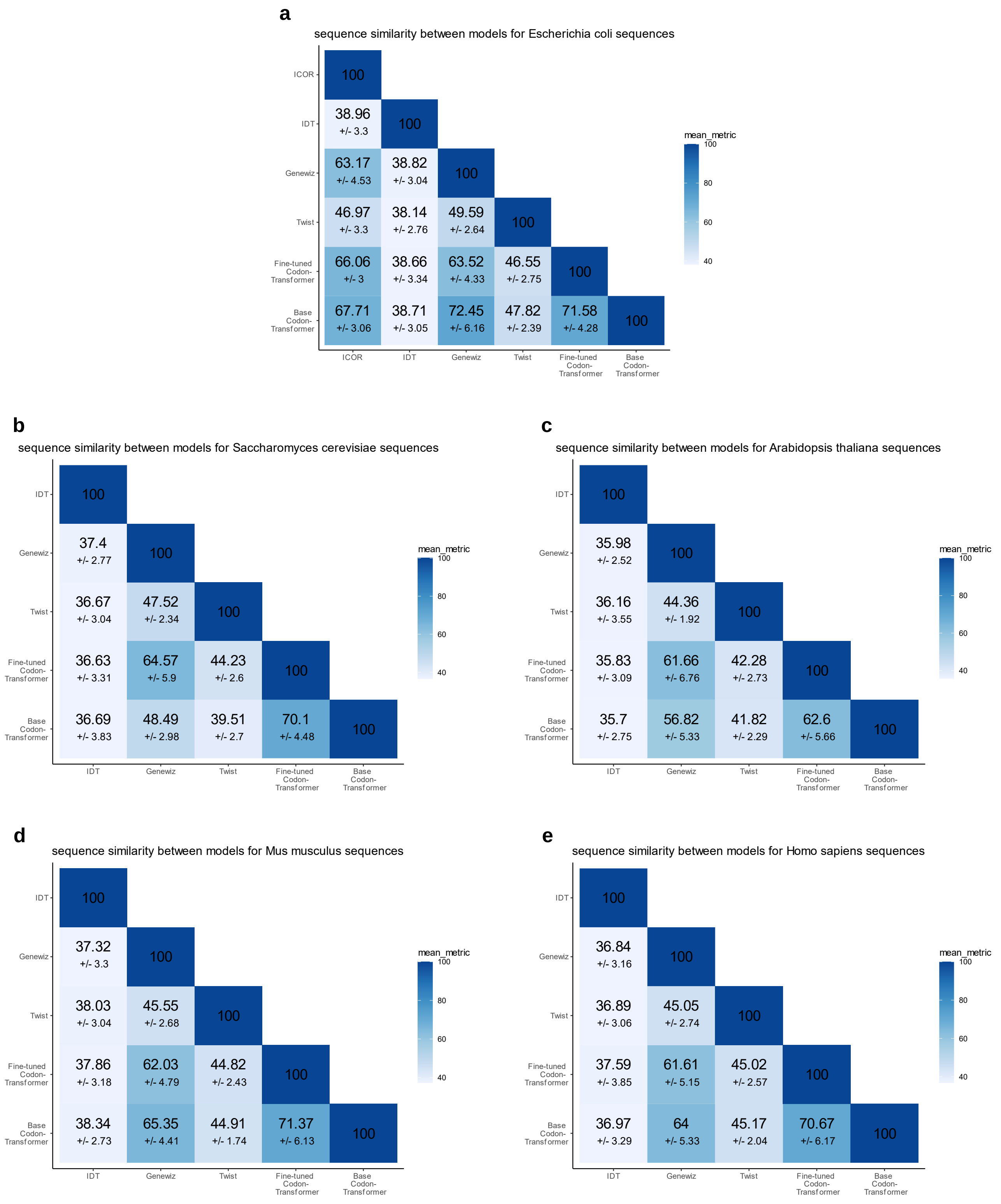

Supplementary Fig. 21: Model comparison based on sequence similarity between sequences generated for 52 benchmark proteins. Mean and standard deviation of sequence similarity between corresponding proteins for *E. coli* (**a**), *S. cerevisiae* (**b**), *A. thaliana* (**c**), *M. musculus* (**d**), *H. sapiens* (**e**). Data underlying this figure is provided in the Source Data file.

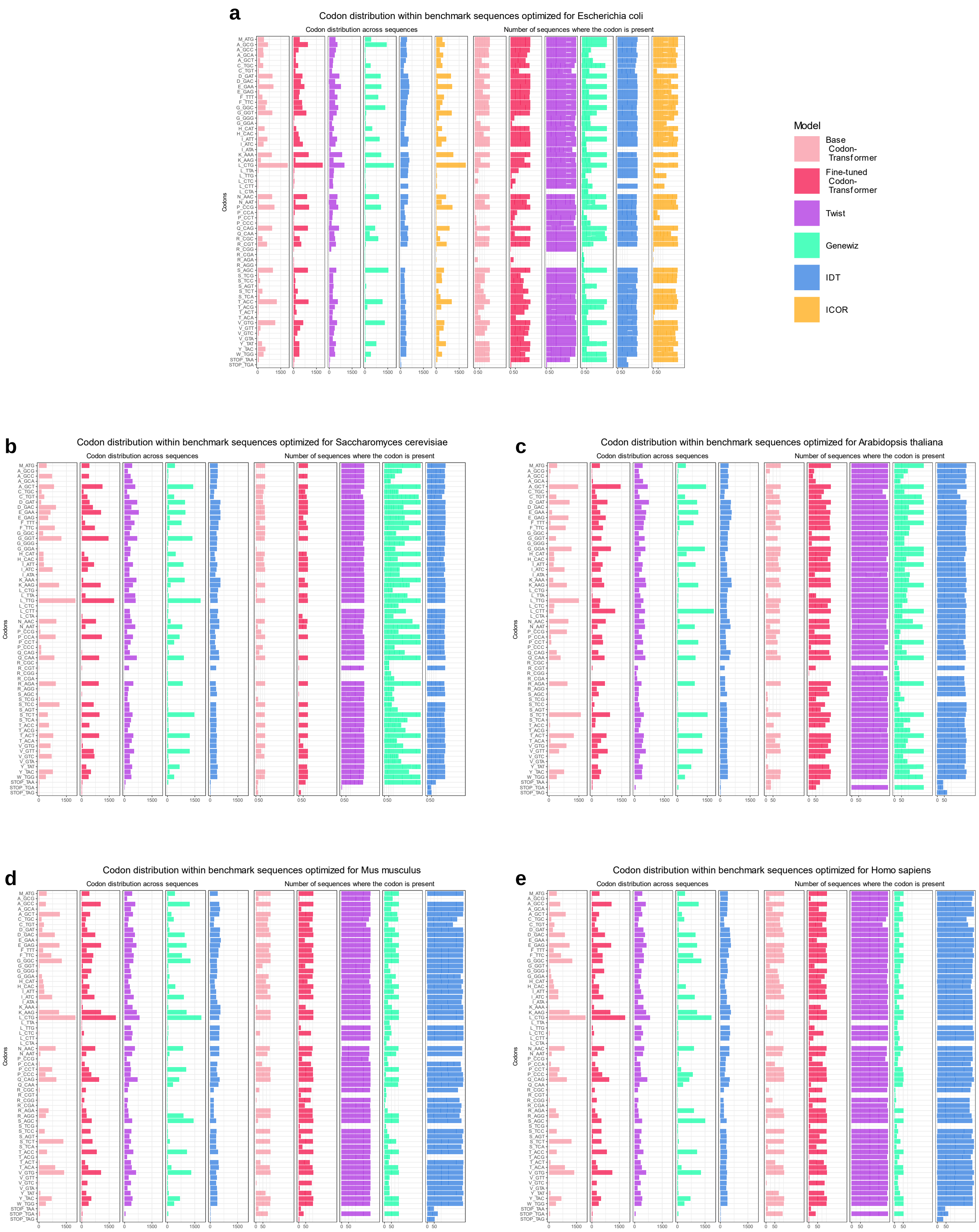

Supplementary Fig. 22: Distribution of 64 codons among 52 benchmark proteins codon-optimized by different models for *E. coli* (**a**), *S. cerevisiae* (**b**), *A. thaliana* (**c**), *M. musculus* (**d**), *H. sapiens* (**e**). Data underlying this figure is provided in the Source Data file.

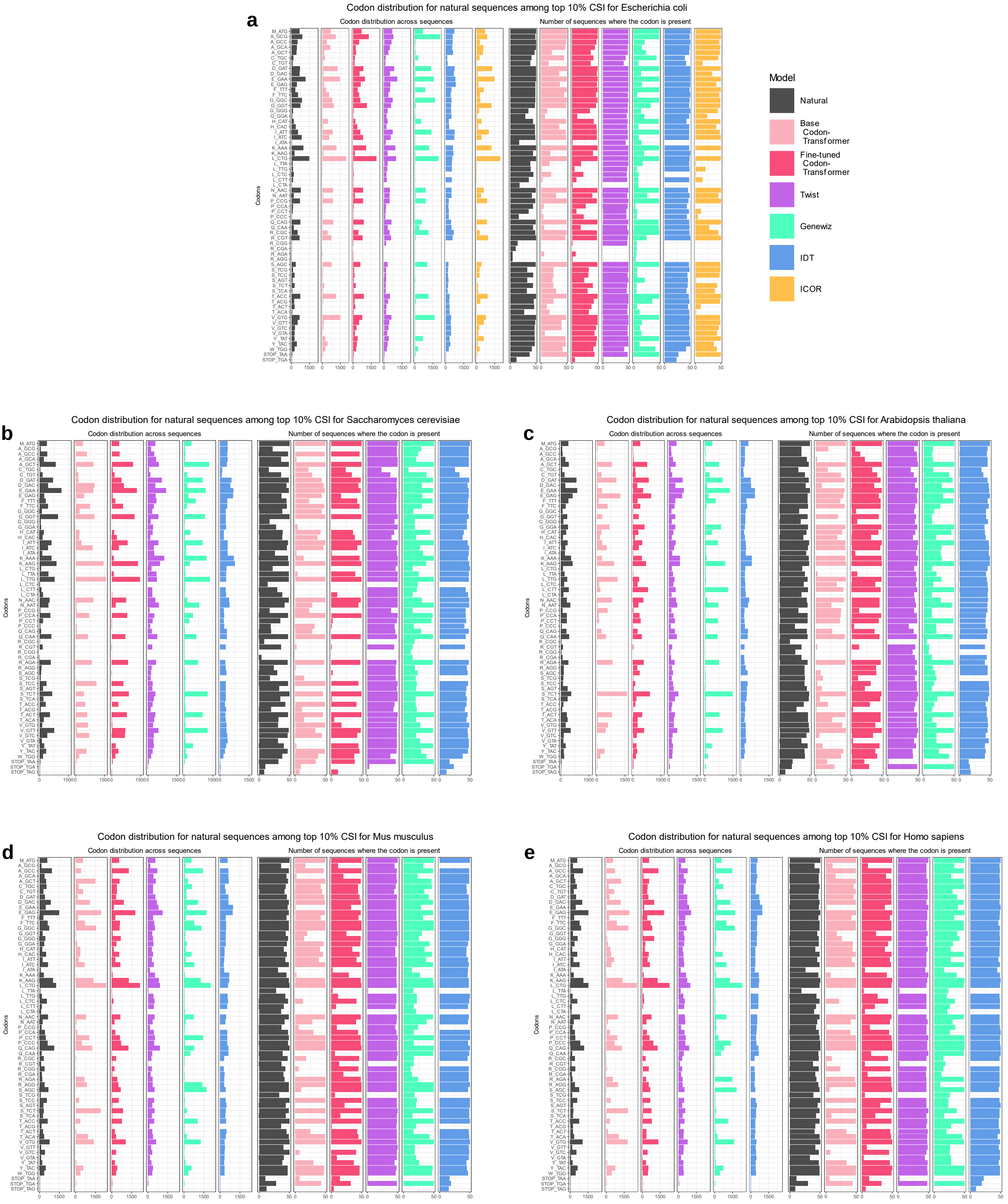

Supplementary Fig. 23: Distribution of 64 codons among 50 random genes selected from the top 10% CSI and their generated counterpart by different models for *E. coli* (**a**), *S. cerevisiae* (**b**), *A. thaliana* (**c**), *M. musculus* (**d**), *H. sapiens* (**e**). Data underlying this figure is provided in the Source Data file.

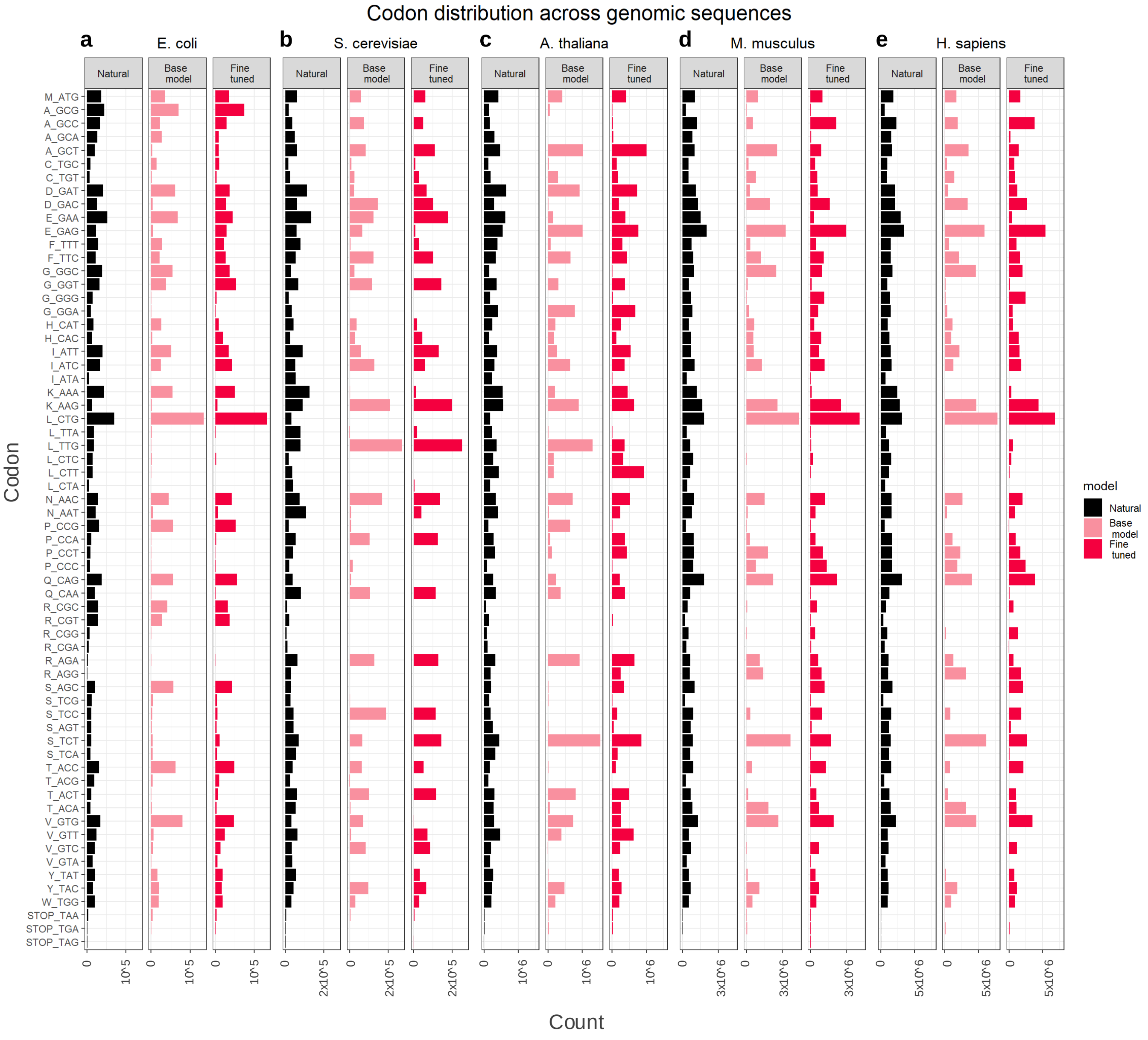

Supplementary Fig. 24 Distribution of 64 codons among genomes and their generated counterpart by base and fine-tuned CodonTransformer for *E. coli* (**a**), *S. cerevisiae* (**b**), *A. thaliana* (**c**), *M. musculus* (**d**), *H. sapiens* (**e**). Data underlying this figure is provided in the Source Data file.

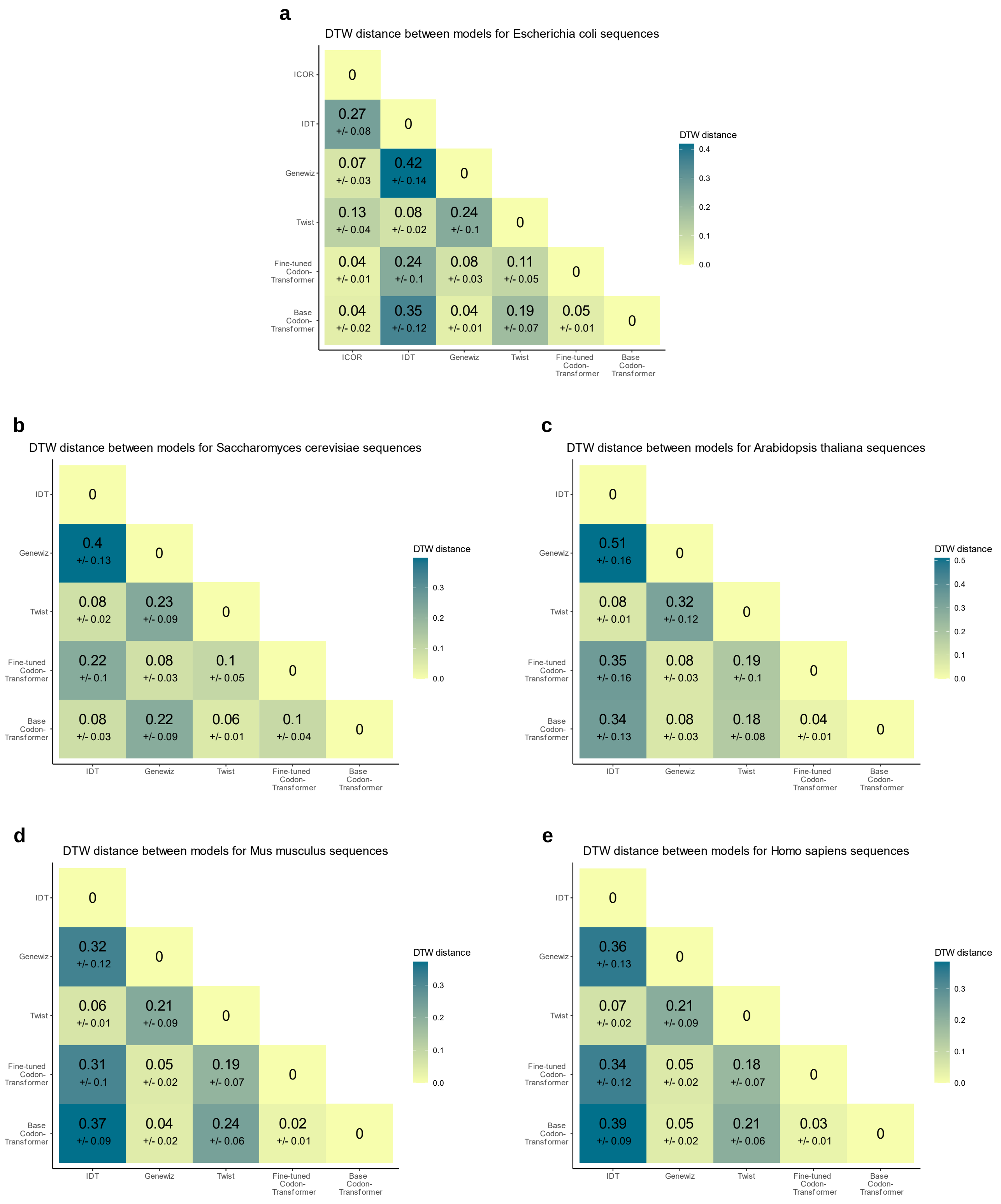

Supplementary Fig. 25: Model comparison based on normalized DTW distances between sequences generated for 52 benchmark proteins. Mean and standard deviation of normalized DTW distances between corresponding proteins for *E. coli* (**a**), *S. cerevisiae* (**b**), *A. thaliana* (**c**), *M. musculus* (**d**), *H. sapiens* (**e**). Data underlying this figure is provided in the Source Data file.

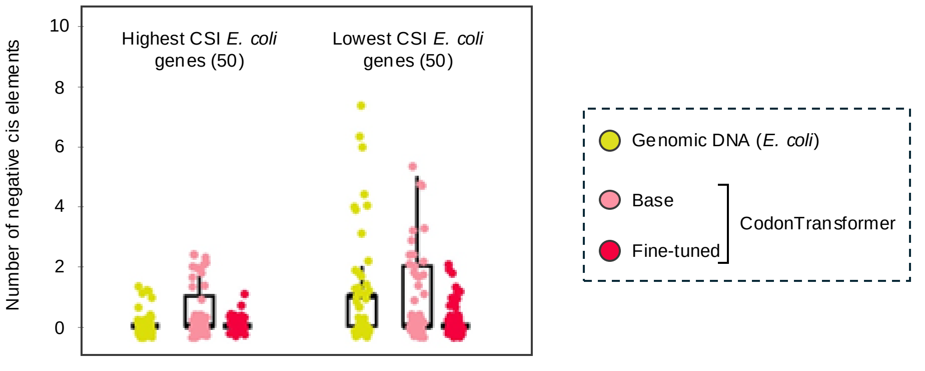

Supplementary Fig. 26: The average number of negative cis-regulatory elements of *E. coli* genes (from the general set), 50 lowest and 50 highest CSI, and for their codon-optimized sequences by the base and fine-tuned CodonTransformer**.**
