# Supplementary material for: CodonTransformer: a multispecies codon optimizer using context-aware neural networks
Source: Nat Commun. 2025 Apr 3;16:3205. doi: 10.1038/s41467-025-58588-7 (PMC11968976; doi:10.1038/s41467-025-58588-7)
Supplement: Supplementary file 2 — Reporting Summary [file 41467_2025_58588_MOESM2_ESM.pdf]

nature portfolio

Corresponding author(s): Guillaume J. Filion, Ariel B. Lindner, Amir Pandi

Last updated by author(s): Feb 19, 2025

Reporting Summary

Nature Portfolio wishes to improve the reproducibility of the work that we publish. This form provides structure for consistency and transparency in reporting. For further information on Nature Portfolio policies, see our Editorial Policies and the Editorial Policy Checklist.

Statistics

For all statistical analyses, confirm that the following items are present in the figure legend, table legend, main text, or Methods section.

n/a	Confirmed	
☐	☒	The exact sample size (*n*) for each experimental group/condition, given as a discrete number and unit of measurement
☒	☐	A statement on whether measurements were taken from distinct samples or whether the same sample was measured repeatedly
☐	☒	The statistical test(s) used AND whether they are one- or two-sided<br>*Only common tests should be described solely by name; describe more complex techniques in the Methods section.*
☒	☐	A description of all covariates tested
☒	☐	A description of any assumptions or corrections, such as tests of normality and adjustment for multiple comparisons
☐	☒	A full description of the statistical parameters including central tendency (e.g. means) or other basic estimates (e.g. regression coefficient) AND variation (e.g. standard deviation) or associated estimates of uncertainty (e.g. confidence intervals)
☐	☒	For null hypothesis testing, the test statistic (e.g. *F*, *t*, *r*) with confidence intervals, effect sizes, degrees of freedom and *P* value noted<br>*Give P values as exact values whenever suitable.*
☒	☐	For Bayesian analysis, information on the choice of priors and Markov chain Monte Carlo settings
☒	☐	For hierarchical and complex designs, identification of the appropriate level for tests and full reporting of outcomes
☒	☐	Estimates of effect sizes (e.g. Cohen's *d*, Pearson's *r*), indicating how they were calculated

Our web collection on statistics for biologists contains articles on many of the points above.

Software and code

Policy information about availability of computer code

Data collection	All data in this study have been collected by custom code that we provided through the Github repository (https://github.com/Adibvafa/CodonTransformer).

Data analysis	Data analysis in this work has been done via a custom code provided in the Github repository (https://github.com/Adibvafa/CodonTransformer).<br>Package versions:<br>biopython>=1.83,<2.0<br>CAI-PyPI>=2.0.1,<3.0<br>ipywidgets>=7.0.0,<10.0<br>numpy>=1.26.4,<3.0<br>onnxruntime>=1.17.3,<3.0<br>pandas>=2.0.0,<3.0<br>python_codon_tables>=0.1.12,<1.0<br>pytorch_lightning>=2.2.1,<3.0<br>scikit-learn>=1.2.2,<2.0<br>scipy>=1.13.1,<3.0<br>setuptools>=70.0.0<br>torch>=2.0.0,<3.0<br>tqdm>=4.66.2,<5.0<br>transformers>=4.40.0,<5.0

For manuscripts utilizing custom algorithms or software that are central to the research but not yet described in published literature, software must be made available to editors and reviewers. We strongly encourage code deposition in a community repository (e.g. GitHub). See the Nature Portfolio guidelines for submitting code & software for further information.

Data

Policy information about availability of data

All manuscripts must include a data availability statement. This statement should provide the following information, where applicable:

- Accession codes, unique identifiers, or web links for publicly available datasets
- A description of any restrictions on data availability
- For clinical datasets or third party data, please ensure that the statement adheres to our policy

All genomic data (164 organisms) used to train the model are available at both https://zenodo.org/records/12509224 and https://huggingface.co/datasets/adibvafa/CodonTransformer. Data for Fig. 2a, Supplementary Figs. 2-16 are available at https://zenodo.org/records/13262517, for Fig. 3 and Supplementary Fig. 18, 19a-e, 23, 24 in Supplementary Data 1, for Fig. 4, Supplementary Fig. 19f-h, 20, 21, 22 and 25, in Supplementary Data 2, and for Fig. 2b in Supplementary Data 3. Supplementary Data 1-3 and the custom code used to produce results in this work are available within the Source Data file. Source Data are provided with this paper.

Research involving human participants, their data, or biological material

Policy information about studies with human participants or human data. See also policy information about sex, gender (identity/presentation), and sexual orientation and race, ethnicity and racism.

Reporting on sex and gender	n/a
Reporting on race, ethnicity, or other socially relevant groupings	n/a
Population characteristics	n/a
Recruitment	n/a
Ethics oversight	n/a

Note that full information on the approval of the study protocol must also be provided in the manuscript.

Field-specific reporting

Please select the one below that is the best fit for your research. If you are not sure, read the appropriate sections before making your selection.

☒ Life sciences ☐ Behavioural & social sciences ☐ Ecological, evolutionary & environmental sciences

For a reference copy of the document with all sections, see nature.com/documents/nr-reporting-summary-flat.pdf

Life sciences study design

All studies must disclose on these points even when the disclosure is negative.

Sample size	No sample size calculation was performed in this study. The data were selected for training to have a diverse set of organisms that include model organisms. And for analysis of natural genomes we randomly selected 50 genes among genes with top 10% CSI (codon similarity index) because of limiting capacity of other tools that we used in this study. However, wherever the aim was not comparing different models, we used the entire genomes of model organisms.
Data exclusions	No data was excluded from the analyses.
Replication	This work includes only computational analyses for which we did not take into account replication. The robustness of the model was demonstrated across organisms (Fig. 2) and sequences (Figs. 3 and 4).
Randomization	We described a codon optimization framework with computational simulation for which randomization is not relevant as such. We selected the organisms for model training based on previous studies that we extensively completed based on prior knowledge and investigation in order to create a diverse training data that includes model and non-model organisms of interest for a broad scientific community.
Blinding	This study focuses on building a tool for efficient codon optimization that we demonstrated its performance across organisms. Therefor, blinding was not applicable to this work that did not involved external individuals/subjective assessments.

Reporting for specific materials, systems and methods

We require information from authors about some types of materials, experimental systems and methods used in many studies. Here, indicate whether each material, system or method listed is relevant to your study. If you are not sure if a list item applies to your research, read the appropriate section before selecting a response.

Materials & experimental systems

n/a	Involved in the study	
☒	☐	Antibodies
☒	☐	Eukaryotic cell lines
☒	☐	Palaeontology and archaeology
☒	☐	Animals and other organisms
☒	☐	Clinical data
☒	☐	Dual use research of concern
☒	☐	Plants

Methods

n/a	Involved in the study	
☒	☐	ChIP-seq
☒	☐	Flow cytometry
☒	☐	MRI-based neuroimaging

Plants

Seed stocks	n/a
Novel plant genotypes	n/a
Authentication	n/a
