# Supplementary material for: CodonTransformer: a multispecies codon optimizer using context-aware neural networks
Source: Nat Commun. 2025 Apr 3;16:3205. doi: 10.1038/s41467-025-58588-7 (PMC11968976; doi:10.1038/s41467-025-58588-7)
Supplement: Supplementary file 4 — Source Data [file 41467_2025_58588_MOESM4_ESM.zip › Source Data/Reproduce_figures.nb.html]

CodonTransformer: a multispecies codon optimizer using context-aware neural networks


Code 

- Show All Code
- Hide All Code
- Download Rmd

# CodonTransformer: a multispecies codon optimizer using context-aware neural networks

- generic variables and
  plotting functions
- natural
  sequences
  - codon
    distribution
  - %minmax
  - selected
    genes
  - plot species
  - DTW
  - RNA fold
- benchmark sequences
  - Codon
    distribution
  - Jaccard
    index
  - Seq
    similarity
  - %minmax
  - DTW
  - Negative cis-elements
  - RNA fold
- genome
  sequences
  - codon
    distribution
- Session info


This code reproduces ggplot figures in “CodonTransformer: a
multispecies codon optimizer using context-aware neural networks”.

It reads natural and benchmark DNA sequences from supplementary files
1 and 2 (which must be placed in the same folder) and create
figures.

Genomic sequences, DTW distances and RNA folding results are saved as
RDS objects and the corresponding chunks of code are flagged as “eval =
FALSE”.

# generic variables and plotting functions


```
# model names matching in different files
model_patterns <- c(
  "atural",
  "base|pretrain",
  "fine",
  "Twist|twist",
  "Genewiz|genewiz",
  "IDT|idt",
  "ICOR|icor"
)

# model naming on figures
model_levels <- c(
  "Natural",
  "Base \n Codon- \n Transformer",
  "Fine-tuned \n Codon- \n Transformer",
  "Twist",
  "Genewiz",
  "IDT",
  "ICOR"
)

# model colors
model_colors <- c(
  "black",
  "#F9909F",
  "#F4003F",
  "#A821DF",
  "#02FFA2",
  "#2074DF",
  "#FFA400"
)

# species color
species_color <- brewer.pal(9, "Set1")[c(7, 4, 3, 9, 8)]

# compute minmax
compute_minmax <- function(df) {
  seq <- kodonz::load.fasta(x = df$sequence)
  res <- kodonz::pminmax(seq[[1]],
    # window length
    z = 18,
    # standard codon table
    y = 0,
    # species for which cut is overided
    spp = df$species
  )
}

# compute Dynamic time warping distances
compute_DTW <- function(df) {
  minmax1 <- df |>
    pull(minmax1) |>
    unlist()
  minmax2 <- df |>
    pull(minmax2) |>
    unlist()
  dtw(minmax1, minmax2)
}

# compute RNA structures
compute_RNA_fold <- function(sequence) {
  # Predict folding and DrG
  XNAString::predictMfeStructure(
    # Convert to XNA object
    XNAString::XNAString(
      base =
      # Store in RNA object
        Biostrings::RNAString(
          # convert DNA to RNA
          x = sequence |> str_replace_all("T", "U")
        )
    )
  )
}

# plot %minmax profiles
plot_line_minmax <- function(.data_structure = NA,
                             .organism = NA,
                             .limits = NA,
                             .ncol = NA) {
  .data_structure |>
    mutate(
      model = factor(model, levels = model_levels),
      name = factor(ifelse("description" %in% colnames(.data_structure),
        description,
        protein
      ))
    ) |>
    filter(str_detect(organism, .organism)) |>
    ggplot(aes(
      y = minmax,
      x = index,
      color = model
    )) +
    geom_line(linewidth = 0.5) +
    lims(y = .limits) +
    # use colors for models present in data
    scale_color_manual(values = model_colors[model_levels %in% .data_structure$model]) +
    facet_wrap(~ name + model, ncol = .ncol, scales = "free_x") +
    ggtitle(paste0("minmax sequences - ", .organism)) +
    theme(plot.title = element_text(hjust = 0.5)) +
    guides(color = "none") +
    theme_bw()
}

# for similarity plots
similarity_plot_height <- 6
similarity_plot_width <- 8

# plot similarity metrics
plot_tile_similarity <- function(.data_struc = NA,
                                 .metric = NA,
                                 .organism = NA) {
  # adapt text size to the number of models
  nb_categories <- length(.data_struc$model2 |> unique())
  data <- .data_struc |>
    filter(str_detect(organism, .organism)) |>
    mutate(
      model1 = factor(model1, levels = rev(model_levels)),
      model2 = factor(model2, levels = rev(levels(model1)))
    ) |>
    group_by(model1, model2) |>
    summarise(
      mean_metric = mean(as.numeric(value)),
      sd_metric = sd(as.numeric(value)),
      .groups = "drop"
    ) |>
    mutate(diagonal = factor(ifelse(model1 == model2, "yes", "no"))) |>
    # filter only the bottom triangle of the heatmap
    mutate(model1_nb = as.numeric(model1)) |>
    mutate(model2_nb = as.numeric(model2)) |>
    filter((model2_nb + model1_nb) <= length(model_levels) + 1) |>
    ggplot(aes(x = model1, y = model2, fill = mean_metric)) +
    geom_tile() +
    # for diagonal, text = centered mean
    geom_text(
      data = ~ subset(., diagonal == "yes"),
      aes(label = paste0(
        round(mean_metric, digits = 2)
      )),
      color = "black",
      size = 35 / nb_categories
    ) +
    # for others, upper text = mean
    geom_text(
      data = ~ subset(., diagonal == "no"),
      aes(label = paste0(
        round(mean_metric, digits = 2),
        "\n"
      )),
      color = "black",
      size = 35 / nb_categories
    ) +
    # for others, lower text = sd
    geom_text(
      data = ~ subset(., diagonal == "no"),
      aes(label = paste0(
        "\n +/- ",
        round(sd_metric, digits = 2)
      )),
      color = "black",
      size = 25 / nb_categories
    ) +
    labs(
      x = "",
      y = "",
      title = paste0(.metric, " between models for ", .organism, " sequences"),
      fill = "Mean"
    ) +
    theme_classic() +
    theme(
      plot.title = element_text(hjust = 0.5, size = 14),
      legend.key.height = unit(1, "cm")
    )
}

plot_jitter_species <- function(.data) {
  .data |>
    ggplot(aes(x = value, y = model, color = organism)) +
    geom_jitter(size = 1, alpha = 0.7, width = 0) +
    scale_color_manual(values = species_color) +
    theme_bw() +
    theme(legend.title = element_blank()) +
    guides(colour = guide_legend(override.aes = list(size = 3)))
}

plot_smooth_model <- function(.data) {
  .data |>
    ggplot(aes(y = y_plot, x = x_plot, color = model)) +
    geom_point(size = 1, alpha = 0.4) +
    geom_smooth(aes(color = model, fill = model), linewidth = 0.5, alpha = 0.3, method = "lm", se = TRUE, formula = "y ~ x") +
    theme_bw() +
    theme(
      strip.text = element_blank(),
      legend.key.spacing.y = unit(0.2, "cm"),
      legend.key.width = unit(1, "cm"),
      legend.key.height = unit(1, "cm"),
      strip.background = element_blank(),
      legend.title = element_blank()
    ) +
    guides(color = "none") +
    guides(fill = guide_legend(override.aes = list(shape = 21, colour = NA)))
}

# 61 codons (20 amino acids) + 3 STOP codons
codon_aa_table <- dplyr::tibble(
  token = c(
    "M_ATG",
    "A_GCG",
    "A_GCC",
    "A_GCA",
    "A_GCT",
    "C_TGC",
    "C_TGT",
    "D_GAT",
    "D_GAC",
    "E_GAA",
    "E_GAG",
    "F_TTT",
    "F_TTC",
    "G_GGC",
    "G_GGT",
    "G_GGG",
    "G_GGA",
    "H_CAT",
    "H_CAC",
    "I_ATT",
    "I_ATC",
    "I_ATA",
    "K_AAA",
    "K_AAG",
    "L_CTG",
    "L_TTA",
    "L_TTG",
    "L_CTC",
    "L_CTT",
    "L_CTA",
    "N_AAC",
    "N_AAT",
    "P_CCG",
    "P_CCA",
    "P_CCT",
    "P_CCC",
    "Q_CAG",
    "Q_CAA",
    "R_CGC",
    "R_CGT",
    "R_CGG",
    "R_CGA",
    "R_AGA",
    "R_AGG",
    "S_AGC",
    "S_TCG",
    "S_TCC",
    "S_AGT",
    "S_TCT",
    "S_TCA",
    "T_ACC",
    "T_ACG",
    "T_ACT",
    "T_ACA",
    "V_GTG",
    "V_GTT",
    "V_GTC",
    "V_GTA",
    "Y_TAT",
    "Y_TAC",
    "W_TGG",
    "STOP_TAA",
    "STOP_TGA",
    "STOP_TAG"
  )
) |>
  mutate(
    codon = str_extract(token, "[A-Z]{3}$"),
    aa = str_extract(token, "^[A-Z]+"),
    token = factor(token, levels = token |> unique() |> rev())
  )


plot_bar_codons_natural <- function(.species = NA,
                                    .save = FALSE) {
  plot_1 <- natural_sequence_data |>
    filter(str_detect(organism, .species)) |>
    mutate(codons = sequence |>
      str_extract_all(pattern = "[:alpha:]{3}")) |>
    select(-sequence) |>
    unnest(codons) |>
    inner_join(codon_aa_table,
      by = c("codons" = "codon")
    ) |>
    ggplot(aes(y = token, fill = model)) +
    geom_bar(alpha = 0.7) +
    scale_fill_manual(values = model_colors) +
    scale_x_continuous(breaks = c(0, 1500)) +
    facet_wrap(~model, nrow = 1) +
    theme_bw() +
    ylab("Codons") +
    xlab("") +
    ggtitle("Codon distribution across sequences") +
    theme(
      plot.title = element_text(hjust = 0.5),
      legend.key.spacing.y = unit(0.2, "cm"),
      legend.key.width = unit(1, "cm"),
      legend.key.height = unit(1, "cm"),
      strip.background = element_blank(),
      strip.text.x = element_blank()
    ) +
    guides(fill = guide_legend(title = "Model"))

  plot_2 <- natural_sequence_data |>
    filter(str_detect(organism, .species)) |>
    mutate(codons = sequence |>
      str_extract_all(pattern = "[:alpha:]{3}")) |>
    select(-sequence) |>
    unnest(codons) |>
    inner_join(codon_aa_table,
      by = c("codons" = "codon")
    ) |>
    group_by(codons, organism, model) |>
    nest(data = description) |>
    mutate(nb_of_sequences = length(unlist(data) |> unique())) |>
    ggplot(aes(y = token, x = nb_of_sequences, fill = model)) +
    geom_col(alpha = 0.7) +
    scale_fill_manual(values = model_colors) +
    scale_x_continuous(breaks = c(0, 50)) +
    facet_wrap(~model, nrow = 1) +
    theme_bw() +
    ylab("Codons") +
    xlab("") +
    ggtitle("Number of sequences where the codon is present") +
    theme(
      plot.title = element_text(hjust = 0.5),
      legend.key.spacing.y = unit(0.2, "cm"),
      legend.key.width = unit(1, "cm"),
      legend.key.height = unit(1, "cm"),
      strip.background = element_blank(),
      strip.text.x = element_blank()
    ) +
    guides(fill = guide_legend(title = "Model"))

  plot <- plot_1 + plot_2 +
    plot_layout(
      widths = c(0.5, 0.5),
      guides = "collect",
      axes = "collect"
    ) +
    plot_annotation(
      title = paste0("Codon distribution for natural sequences among top 10% CSI for ", .species),
      theme = theme(plot.title = element_text(hjust = 0.5)) &
        theme(plot.title = element_text(size = 18))
    )

  if (.save == TRUE) {
    ggsave(plot,
      filename = paste0(
        "codon_distribution_natural_sequences_",
        .species,
        ".svg"
      ),
      height = 10,
      width = 14
    )
  }
  plot
}

plot_bar_codons_benchmark <- function(.species = NA,
                                      .save = FALSE) {
  plot_1 <- benchmark_sequence_data |>
    filter(str_detect(organism, .species)) |>
    mutate(codons = sequence |>
      str_extract_all(pattern = "[:alpha:]{3}")) |>
    select(-sequence) |>
    unnest(codons) |>
    inner_join(codon_aa_table,
      by = c("codons" = "codon")
    ) |>
    ggplot(aes(y = token, fill = model)) +
    geom_bar(alpha = 0.7) +
    scale_fill_manual(values = model_colors[-1]) +
    scale_x_continuous(breaks = c(0, 1500)) +
    facet_wrap(~model, nrow = 1) +
    theme_bw() +
    ylab("Codons") +
    xlab("") +
    ggtitle("Codon distribution across sequences") +
    theme(
      plot.title = element_text(hjust = 0.5),
      legend.key.spacing.y = unit(0.2, "cm"),
      legend.key.width = unit(1, "cm"),
      legend.key.height = unit(1, "cm"),
      strip.background = element_blank(),
      strip.text.x = element_blank()
    ) +
    guides(fill = guide_legend(title = "Model"))

  plot_2 <- benchmark_sequence_data |>
    filter(str_detect(organism, .species)) |>
    mutate(codons = sequence |>
      str_extract_all(pattern = "[:alpha:]{3}")) |>
    select(-sequence) |>
    unnest(codons) |>
    inner_join(codon_aa_table,
      by = c("codons" = "codon")
    ) |>
    group_by(codons, organism, model) |>
    nest(data = protein) |>
    mutate(nb_of_sequences = length(unlist(data) |> unique())) |>
    ggplot(aes(y = token, x = nb_of_sequences, fill = model)) +
    geom_col(alpha = 0.7) +
    scale_fill_manual(values = model_colors[-1]) +
    scale_x_continuous(breaks = c(0, 50)) +
    facet_wrap(~model, nrow = 1) +
    theme_bw() +
    ylab("Codons") +
    xlab("") +
    ggtitle("Number of sequences where the codon is present") +
    theme(
      plot.title = element_text(hjust = 0.5),
      legend.key.spacing.y = unit(0.2, "cm"),
      legend.key.width = unit(1, "cm"),
      legend.key.height = unit(1, "cm"),
      strip.background = element_blank(),
      strip.text.x = element_blank()
    ) +
    guides(fill = guide_legend(title = "Model"))

  plot <- plot_1 + plot_2 +
    plot_layout(
      widths = c(0.5, 0.5),
      guides = "collect",
      axes = "collect"
    ) +
    plot_annotation(
      title = paste0("Codon distribution within benchmark sequences optimized for ", .species),
      theme = theme(plot.title = element_text(hjust = 0.5)) &
        theme(plot.title = element_text(size = 18))
    )


  if (.save == TRUE) {
    ggsave(plot,
      filename = paste0(
        "codon_distribution_benchmark_sequences_",
        .species,
        ".svg"
      ),
      height = 10,
      width = 14
    )
  }
  plot
}
```

# natural sequences


```
natural_sequence_data <- read_xlsx("Supplementary Data 1.xlsx", sheet = "DNA sequences") |>
  mutate(organism = organism |> str_remove(" str. K-12 substr. MG1655")) |>
  pivot_longer(
    cols = matches("DNA"),
    names_to = "model",
    values_to = "sequence"
  ) |>
  mutate(model = model |>
    str_remove("_.*$")) |>
  # ICOR are NA for other organisms
  drop_na() |>
  mutate(model = factor(
    ifelse(
      str_detect(model, model_patterns[1]),
      model_levels[1],
      ifelse(
        str_detect(model, model_patterns[2]),
        model_levels[2],
        ifelse(
          str_detect(model, model_patterns[3]),
          model_levels[3],
          ifelse(
            str_detect(model, model_patterns[4]),
            model_levels[4],
            ifelse(
              str_detect(model, model_patterns[5]),
              model_levels[5],
              ifelse(
                str_detect(model, model_patterns[6]),
                model_levels[6],
                ifelse(
                  str_detect(model, model_patterns[7]),
                  model_levels[7],
                  NA
                )
              )
            )
          )
        )
      )
    ),
    levels = model_levels
  )) |>
  select(organism, description, model, sequence)

organism_levels <- natural_sequence_data |>
  pull(organism) |>
  unique()
```


```
natural_sequence_data |>
  group_by(organism, model) |>
  summarise(
    nb_of_sequences = n(),
    .groups = "drop"
  )
```


## codon distribution


```
natural_sequence_data |> 
  mutate(codon=sequence |> str_extract_all("[A-Z]{3}")) |> 
  unnest_longer(codon) |> 
  inner_join(codon_aa_table, by=join_by(codon==codon)) |> 
  mutate(model=model |> str_remove_all("\n")) |> 
  group_by(organism, model, token) |> 
  summarise(codon_count=n()) |> 
  write_tsv("natural_sequences_codon_counts.tsv")
```


```
`summarise()` has grouped output by 'organism', 'model'. You can override
using the `.groups` argument.
```


```
plot_bar_codons_natural(
  .species = "Escherichia coli",
  .save = FALSE
)
```


```
plot_bar_codons_natural(
  .species = "Saccharomyces cerevisiae",
  .save = FALSE
)
```


```
plot_bar_codons_natural(
  .species = "Arabidopsis thaliana",
  .save = FALSE
)
```


```
plot_bar_codons_natural(
  .species = "Mus musculus",
  .save = FALSE
)
```


```
plot_bar_codons_natural(
  .species = "Homo sapiens",
  .save = FALSE
)
```

## %minmax


```
natural_minmax_results <- natural_sequence_data |>
  select(organism, description, model, sequence) |>
  mutate(species = ifelse(str_detect(organism, "coli"), "ecoli",
    ifelse(str_detect(organism, "cerevisiae"), "saccharomyces",
      ifelse(str_detect(organism, "thaliana"), "arabidopsis",
        ifelse(str_detect(organism, "musculus"), "mouse",
          ifelse(str_detect(organism, "sapiens"), "human", NA)
        )
      )
    )
  )) |>
  nest(data = c(sequence, species)) |>
  mutate(minmax = map(data, compute_minmax)) |>
  unnest(data) |>
  unnest(minmax) |>
  group_by(organism, model, description) |>
  mutate(index = row_number())
```

## selected genes

Ec: NC\_000913.3:338325-339743 yahG

Sc: NM\_001179424.1:1-1410 SER33

At: NM\_001340786.1:277-1587 AT4G12540

Mm: NM\_001416453.1:324-1805 Csad

Hs: XM\_054318276.1:337-2223 ZBTB7C


```
plot <- natural_minmax_results |>
  filter(str_detect(
    description,
    "NC_000913.3:338325-339743 yahG|NM_001179424.1:1-1410 SER33|NM_001340786.1:277-1587 AT4G12540|NM_001416453.1:324-1805 Csad|XM_054318276.1:337-2223 ZBTB7C"
  )) |>
  mutate(description = description |>
    str_extract(" .*$") |>
    str_remove(" ")) |>
  mutate(description = factor(description,
    levels = c(
      "yahG",
      "SER33",
      "AT4G12540",
      "Csad",
      "ZBTB7C"
    )
  )) |>
  mutate(value = minmax * 100) |>
  ggplot(aes(
    y = value,
    x = index,
    color = model
  )) +
  geom_line(linewidth = 0.5) +
  scale_color_manual(values = model_colors) +
  facet_grid(model ~ description,
    scales = "free_x"
  ) +
  labs(x = "", y = "") +
  theme_bw() +
  theme(
    legend.position = "none",
    theme(
      legend.position = "none",
      strip.background = element_blank(),
      panel.border = element_blank()
    )
  ) +
  theme(strip.background = element_blank())
plot
```


```
ggsave(
  plot = plot,
  filename = "selected_minmax.svg",
  height = 8,
  width = 10
)
```

## plot species


```
plot <- plot_line_minmax(
  .data_structure = natural_minmax_results,
  .organism = organism_levels[1],
  .limits = c(-1, 1.2),
  .ncol = 7
)

# ggsave(plot,
#   filename = "minmax_natural_Ecoli_w=18.svg",
#   height = 120,
#   width = 20,
#   limitsize = FALSE
# )
```


```
plot <- plot_line_minmax(
  .data_structure = natural_minmax_results,
  .organism = organism_levels[2],
  .limits = c(-1, 1.2),
  .ncol = 6
)

# ggsave(plot,
#   filename = "minmax_natural_Scerevisiae_w=18.svg",
#   height = 120,
#   width = 16,
#   limitsize = FALSE
# )
```


```
plot <- plot_line_minmax(
  .data_structure = natural_minmax_results,
  .organism = organism_levels[3],
  .limits = c(-1, 1.2),
  .ncol = 6
)

# ggsave(plot,
#   filename = "minmax_natural_Athaliana_w=18.svg",
#   height = 120,
#   width = 16,
#   limitsize = FALSE
# )
```


```
plot <- plot_line_minmax(
  .data_structure = natural_minmax_results,
  .organism = organism_levels[4],
  .limits = c(-1, 1.2),
  .ncol = 6
)

# ggsave(plot,
#   filename = "minmax_natural_Mmusculus_w=18.svg",
#   height = 120,
#   width = 16,
#   limitsize = FALSE
# )
```


```
plot <- plot_line_minmax(
  .data_structure = natural_minmax_results,
  .organism = organism_levels[5],
  .limits = c(-1, 1.2),
  .ncol = 6
)

# ggsave(plot,
#   filename = "minmax_natural_Hsapiens_w=18.svg",
#   height = 120,
#   width = 16,
#   limitsize = FALSE
# )
```


```
natural_minmax_results |>
  select(-species, -sequence) |>
  mutate(
    model = model |> str_remove_all("\n"),
    minmax = minmax |> round(digits = 2)
  ) |>
  pivot_wider(names_from = index, values_from = minmax) |>
  unite(col = "minmax", matches("\\d"), sep = ",") |>
  mutate(minmax = minmax |> str_remove_all(",NA")) |>
  write_tsv("natural_minmax_profiles.tsv")
```

## DTW


```
natural_DTW_results <- natural_minmax_results |>
  select(-species, -index, -sequence) |>
  group_by(organism, description, model) |>
  # minmax results are in long format
  nest(minmax = minmax) |>
  # duplicate models
  mutate(
    model1 = model, minmax1 = minmax,
    model2 = model, minmax2 = minmax
  ) |>
  # link model and minmax profiles
  nest(
    comparison1 = c(model1, minmax1),
    comparison2 = c(model2, minmax2)
  ) |>
  ungroup() |>
  select(organism, description, comparison1, comparison2) |>
  group_by(organism, description) |>
  # make all comparison between c(model,profiles)
  complete(comparison1, comparison2) |>
  unnest(c(comparison1, comparison2)) |>
  nest(dtw_data = c(minmax1, minmax2)) |>
  # compute DTW
  mutate(dtw_object = map(dtw_data, compute_DTW)) |>
  mutate(
    distance = map(dtw_object, pluck("distance")),
    normalized_distance = map(dtw_object, pluck("normalizedDistance"))
  ) |>
  mutate(
    distance = parse_number(distance),
    normalized_distance = parse_number(normalized_distance)
  ) |>
  select(organism, description, model1, model2, distance, normalized_distance)

saveRDS(natural_DTW_results, "natural_DTW_results.rds")
```


```
natural_DTW_results <- readRDS(file = "natural_DTW_results.rds") |>
  mutate(
    distance = as.numeric(distance),
    normalized_distance = as.numeric(normalized_distance)
  )
```


```
dtw_gene_ecoli <- dtw::dtw(
  natural_minmax_results |>
    dplyr::filter(
      organism == "Escherichia coli",
      description == "NC_000913.3:338325-339743 yahG"
    ) |>
    dplyr::filter(model == "Natural") |>
    dplyr::pull(minmax) * 100,
  natural_minmax_results |>
    dplyr::filter(
      organism == "Escherichia coli",
      description == "NC_000913.3:338325-339743 yahG"
    ) |>
    dplyr::filter(model == model_levels[3]) |>
    dplyr::pull(minmax) * 100,
  keep = TRUE
)

dtw::dtwPlotTwoWay(dtw_gene_ecoli,
  ylab = "Natural sequence",
  xlab = "Model sequence",
  ts.type = "l",
  lty = 1,
  lwd = 3,
  offset = 80,
  match.col = "grey"
)
```


#### Ec


```
plot <- plot_tile_similarity(
  .data_struc = natural_DTW_results |>
    mutate(value = normalized_distance),
  .organism = organism_levels[1],
  .metric = "DTW distance"
) +
  scale_fill_gradient(
    low = "#F7FEAD",
    high = "#01708B"
  )

plot
```


```
# ggsave(plot,
#   filename = "DTW_ecoli_natural.svg",
#   height = similarity_plot_height,
#   width = similarity_plot_width
# )
```

#### Sc


```
plot <- plot_tile_similarity(
  .data_struc = natural_DTW_results |>
    mutate(value = normalized_distance),
  .organism = organism_levels[2],
  .metric = "DTW distance"
) +
  scale_fill_gradient(
    low = "#F7FEAD",
    high = "#01708B"
  )

plot
```


```
# ggsave(plot,
#   filename = "DTW_scerevisiae_natural.svg",
#   height = similarity_plot_height,
#   width = similarity_plot_width
# )
```

#### At


```
plot <- plot_tile_similarity(
  .data_struc = natural_DTW_results |>
    mutate(value = normalized_distance),
  .organism = organism_levels[3],
  .metric = "DTW distance"
) +
  scale_fill_gradient(
    low = "#F7FEAD",
    high = "#01708B"
  )

plot
```


```
# ggsave(plot,
#   filename = "DTW_Athaliana_natural.svg",
#   height = similarity_plot_height,
#   width = similarity_plot_width
# )
```

#### Mm


```
plot <- plot_tile_similarity(
  .data_struc = natural_DTW_results |>
    mutate(value = normalized_distance),
  .organism = organism_levels[4],
  .metric = "DTW distance"
) +
  scale_fill_gradient(
    low = "#F7FEAD",
    high = "#01708B"
  )

plot
```


```
# ggsave(plot,
#   filename = "DTW_mmusculus_natural.svg",
#   height = similarity_plot_height,
#   width = similarity_plot_width
# )
```

#### Hs


```
plot <- plot_tile_similarity(
  .data_struc = natural_DTW_results |>
    mutate(value = normalized_distance),
  .organism = organism_levels[5],
  .metric = "DTW distance"
) +
  scale_fill_gradient(
    low = "#F7FEAD",
    high = "#01708B"
  )

plot
```


```
# ggsave(plot,
#   filename = "DTW_hsapiens_natural.svg",
#   height = similarity_plot_height,
#   width = similarity_plot_width
# )
```

#### all


```
plot <- plot_tile_similarity(
  .data_struc = natural_DTW_results |>
    mutate(value = normalized_distance),
  .organism = ".*",
  .metric = "DTW distance"
) +
  scale_fill_gradient(
    low = "#F7FEAD",
    high = "#01708B"
  )

plot
```


```
# ggsave(plot,
#   filename = "DTW_distances_between_models_for_natural_genes.svg",
#   height = similarity_plot_height,
#   width = similarity_plot_width
# )
```

### natural DTW distances


```
dtw_between_natural_and_model <- natural_DTW_results |>
  filter(model1 == "Natural") |>
  filter(model2 != "Natural") |>
  mutate(model = model2) |>
  mutate(organism = factor(organism, levels = organism_levels)) |>
  drop_na()
```


```
stat.test_distance <- dtw_between_natural_and_model |>
  group_by(organism) |>
  t_test(distance ~ model, ref.group = model_levels[3])  
stat.test_distance
```


```
stat.test_distance <- stat.test_distance |>
  add_y_position(
    scales = "free_y",
    step.increase = 0.2
  ) |>
  mutate(p.adj.signif = ifelse(p.adj.signif == "ns", "", p.adj.signif))

plot <- dtw_between_natural_and_model |>
  ggplot(aes(x = model, y = distance, color = model)) +
  geom_boxplot(color = "black", outlier.shape = NA) +
  geom_jitter(
    data = ~ subset(.x, description != "NC_000913.3:338325-339743 yahG" &
      description != "NM_001179424.1:1-1410 SER33" &
      description != "NM_001340786.1:277-1587 AT4G12540" &
      description != "NM_001416453.1:324-1805 Csad" &
      description != "XM_054318276.1:337-2223 ZBTB7C"),
    width = 0.2, height = 0, size = 1, alpha = 0.5
  ) +
  scale_color_manual(values = model_colors[c(-1)]) +
  geom_jitter(
    data = ~ subset(.x, description == "NC_000913.3:338325-339743 yahG" |
      description == "NM_001179424.1:1-1410 SER33" |
      description == "NM_001340786.1:277-1587 AT4G12540" |
      description == "NM_001416453.1:324-1805 Csad" |
      description == "XM_054318276.1:337-2223 ZBTB7C"),
    color = "grey30", height = 0, size = 2, alpha = 0.7
  ) +
  stat_pvalue_manual(
    stat.test_distance,
    bracket.nudge.y = -2, hide.ns = FALSE,
    label = "{scales::scientific(p.adj)} {p.adj.signif}",
    tip.length = 0
  ) +
  scale_y_continuous(expand = expansion(mult = c(0, 0.1))) +
  facet_wrap(~organism, ncol = 5, scales = "free") +
  labs(y = "", x = "") +
  theme_bw() +
  theme(
    axis.title.x = element_blank(),
    axis.text.x = element_blank(),
    legend.title = element_blank(),
    legend.position = "none",
    strip.text = element_text(face = "italic")
  )
plot
```


```
# ggsave(plot,
#   filename = "DTW_to_natural_all_species_with_statistics.svg", height = 3, width = 15
# )
```


```
natural_DTW_results |>
  mutate(
    model1 = model1 |> str_remove_all("\n"),
    model2 = model2 |> str_remove_all("\n")
  ) |>
  select(organism, description, model1, model2, distance, normalized_distance) |>
  mutate(
    distance = unlist(distance),
    normalized_distance = unlist(normalized_distance)
  ) |>
  write_tsv("natural_DTW_minmax.tsv")
```

## RNA fold


```
natural_RNA_folding_results <- natural_sequence_data |>
  mutate(RNA_fold_object = map(sequence, compute_RNA_fold)) |>
  select(organism, description, model, sequence, RNA_fold_object)

saveRDS(natural_RNA_folding_results, "natural_RNA_folding.rds")
```


```
natural_RNA_folding_results <- readRDS("natural_RNA_folding.rds") |> mutate(
  organism = factor(organism, levels = organism_levels),
  mfe = map(RNA_fold_object, pluck("mfe")),
  mfe = as.numeric(mfe),
  length = str_count(sequence),
  mfe_normalized_by_length = mfe / length,
  GC_content = str_count(sequence, "G|C") / length
)
```


#### mfe # length


```
plot <- natural_RNA_folding_results |>
  mutate(y_plot = mfe, x_plot = length) |>
  plot_smooth_model() +
  scale_color_manual(values = model_colors) +
  scale_fill_manual(values = model_colors)
plot
```


```
# ggsave(plot,
#   filename = "RNA_folding_1_linear_relation_to_length_natural.svg",
#   height = 4,
#   width = 6
# )
```

#### normalized # natural


```
plot <- inner_join(
  natural_RNA_folding_results |>
    filter(model != "Natural"),
  natural_RNA_folding_results |>
    filter(model == "Natural") |>
    mutate(ref_norm = mfe_normalized_by_length) |>
    select(-mfe_normalized_by_length, -model),
  by = c("organism", "description", "length")
) |>
  mutate(organism = factor(organism, levels = organism_levels)) |>
  mutate(x_plot = ref_norm, y_plot = mfe_normalized_by_length) |>
  plot_smooth_model() +
  stat_regline_equation(
    label.x.npc = 0.6,
    label.y.npc = 0.35,
  ) +
  ylab("mfe / length") +
  xlab("mfe / length for natural sequence") +
  xlim(c(-0.55, -0.1)) +
  scale_color_manual(values = model_colors[-1]) +
  scale_fill_manual(values = model_colors[-1])
plot
```


```
# ggsave(plot,
#   filename = "RNA_folding_2_linear_fit_plotnormalized_natural.svg",
#   height = 4,
#   width = 6
# )
```

#### normalized by species


```
plot <- natural_RNA_folding_results |>
  mutate(value = mfe_normalized_by_length) |>
  plot_jitter_species() +
  xlab("mfe / length")
plot
```


```
# ggsave(plot,
#   filename = "RNA_folding_3_species_separation_normalized_natural.svg",
#   height = 4,
#   width = 6
# )
```

#### GC by species


```
plot <- natural_RNA_folding_results |>
  mutate(value = GC_content) |>
  plot_jitter_species() +
  xlab("GC content")
plot
```


```
# ggsave(plot,
#   filename = "RNA_folding_4_species_separation_GC_content.svg",
#   height = 4,
#   width = 6
# )
```

#### GC # natural


```
plot <- inner_join(
  natural_RNA_folding_results |>
    filter(model != "Natural"),
  natural_RNA_folding_results |>
    filter(model == "Natural") |>
    mutate(ref_GC = GC_content) |>
    select(-GC_content, -model),
  by = c("organism", "description", "length")
) |>
  mutate(organism = factor(organism, levels = organism_levels)) |>
  mutate(x_plot = ref_GC, y_plot = GC_content) |>
  plot_smooth_model() +
  stat_regline_equation(
    label.x.npc = 0.6,
    label.y.npc = 0.35,
  ) +
  ylab("GC content") +
  xlab("GC content of natural sequence") +
  scale_color_manual(values = model_colors[-1]) +
  scale_fill_manual(values = model_colors[-1])

plot
```


```
# ggsave(plot,
#   filename = "RNA_folding_5_linear_fit_GCcontent_natural.svg",
#   height = 4,
#   width = 6
# )
```


```
natural_RNA_folding_results |>
  mutate(model = model |> str_remove_all("\n")) |>
  select(organism, description, mfe, length, mfe_normalized_by_length, GC_content) |>
  write_tsv("natural_RNA_fold.tsv")
```

# benchmark sequences


```
# import sequences generated by different tools
benchmark_sequence_data <- read_xlsx("Supplementary Data 2.xlsx", sheet = "DNA sequences, CSI, CIS-element") |>
  select(-matches("CSI")) |>
  # make columns containing dna sequence as rows
  pivot_longer(
    cols = matches("DNA"),
    names_to = "model",
    values_to = "sequence"
  ) |>
  # make columns containing CSI as rows
  pivot_longer(
    cols = matches("CIS"),
    names_to = "model_bis",
    values_to = "nb_cis_element"
  ) |>
  mutate(
    model = model |> str_remove("_dna$|_DNA$"),
    model_bis = model_bis |> str_remove("_CIS$")
  ) |>
  filter(model == model_bis) |>
  # ICOR are NA for other organisms
  drop_na() |>
  mutate(model = factor(
    ifelse(
      str_detect(model, model_patterns[1]),
      model_levels[1],
      ifelse(
        str_detect(model, model_patterns[2]),
        model_levels[2],
        ifelse(
          str_detect(model, model_patterns[3]),
          model_levels[3],
          ifelse(
            str_detect(model, model_patterns[4]),
            model_levels[4],
            ifelse(
              str_detect(model, model_patterns[5]),
              model_levels[5],
              ifelse(
                str_detect(model, model_patterns[6]),
                model_levels[6],
                ifelse(
                  str_detect(model, model_patterns[7]),
                  model_levels[7],
                  NA
                )
              )
            )
          )
        )
      )
    ),
    levels = model_levels
  )) |>
  select(organism, protein, model, sequence, nb_cis_element)

organism_levels <- benchmark_sequence_data |>
  pull(organism) |>
  unique()
```


```
benchmark_sequence_data |>
  group_by(organism, model) |>
  summarise(nb_of_sequences = n(), .groups = "drop")
```


## Codon distribution


```
benchmark_sequence_data |> 
  mutate(codon=sequence |> str_extract_all("[A-Z]{3}")) |> 
  unnest_longer(codon) |> 
  inner_join(codon_aa_table, by=join_by(codon==codon)) |> 
  mutate(model=model |> str_remove_all("\n")) |> 
  group_by(organism, model, token) |> 
  summarise(codon_count=n()) |> 
  write_tsv("benchmark_sequences_codon_counts.tsv")
```


```
`summarise()` has grouped output by 'organism', 'model'. You can override
using the `.groups` argument.
```


```
plot_bar_codons_benchmark(
  .species = "Escherichia coli",
  .save = FALSE
)
```


```
plot_bar_codons_benchmark(
  .species = "Saccharomyces cerevisiae",
  .save = FALSE
)
```


```
plot_bar_codons_benchmark(
  .species = "Arabidopsis thaliana",
  .save = FALSE
)
```


```
plot_bar_codons_benchmark(
  .species = "Mus musculus",
  .save = FALSE
)
```


```
plot_bar_codons_benchmark(
  .species = "Homo sapiens",
  .save = FALSE
)
```

## Jaccard index


```
# function to compute Jaccard index for two input sequences
jaccard_similarity <- function(df) {
  A <- df$codons1 |> unlist()
  B <- df$codons2 |> unlist()
  if (length(A) != length(B)) {
    return("different length")
  } else if (is.null(A) | is.null(B)) {
    return(NA)
  } else {
    intersection <- length(base::intersect(A, B))
    union <- length(base::union(A, B))
    return(intersection / union)
  }
}

benchmark_jaccard_results <- benchmark_sequence_data |>
  mutate(codons = sequence |>
    str_extract_all(pattern = "[:alpha:]{3}")) |>
  mutate(
    model1 = model, codons1 = codons,
    model2 = model, codons2 = codons
  ) |>
  nest(
    comparison1 = c(model1, codons1),
    comparison2 = c(model2, codons2)
  ) |>
  select(organism, protein, comparison1, comparison2) |>
  group_by(organism, protein) |>
  complete(comparison1, comparison2) |>
  unnest(c(comparison1, comparison2)) |>
  nest(jaccard_data = c(codons1, codons2)) |>
  mutate(value = map(jaccard_data, jaccard_similarity))
```


#### Ec


```
plot <- plot_tile_similarity(
  .data_struc = benchmark_jaccard_results,
  .organism = organism_levels[1],
  .metric = "Jaccard index"
) +
  scale_fill_distiller(
    palette = "Greens",
    direction = 1
  )
plot
```


```
# ggsave(plot,
#   filename = "jaccard_ecoli.svg",
#   height = similarity_plot_height,
#   width = similarity_plot_width
# )
```

#### Sc


```
plot <- plot_tile_similarity(
  .data_struc = benchmark_jaccard_results,
  .organism = organism_levels[2],
  .metric = "Jaccard index"
) +
  scale_fill_distiller(
    palette = "Greens",
    direction = 1
  )
plot
```


```
# ggsave(plot,
#   filename = "jaccard_scerevisiae.svg",
#   height = similarity_plot_height,
#   width = similarity_plot_width
# )
```

#### At


```
plot <- plot_tile_similarity(
  .data_struc = benchmark_jaccard_results,
  .organism = organism_levels[3],
  .metric = "Jaccard index"
) +
  scale_fill_distiller(
    palette = "Greens",
    direction = 1
  )
plot
```


```
# ggsave(plot,
#   filename = "jaccard_athaliana.svg",
#   height = similarity_plot_height,
#   width = similarity_plot_width
# )
```

#### Mm


```
plot <- plot_tile_similarity(
  .data_struc = benchmark_jaccard_results,
  .organism = organism_levels[4],
  .metric = "Jaccard index"
) +
  scale_fill_distiller(
    palette = "Greens",
    direction = 1
  )
plot
```


```
# ggsave(plot,
#   filename = "jaccard_mmusculus.svg",
#   height = similarity_plot_height,
#   width = similarity_plot_width
# )
```

#### Hs


```
plot <- plot_tile_similarity(
  .data_struc = benchmark_jaccard_results,
  .organism = organism_levels[5],
  .metric = "Jaccard index"
) +
  scale_fill_distiller(
    palette = "Greens",
    direction = 1
  )
plot
```


```
# ggsave(plot,
#   filename = "jaccard_hsapiens.svg",
#   height = similarity_plot_height,
#   width = similarity_plot_width
# )
```

### all


```
plot <- plot_tile_similarity(
  .data_struc = benchmark_jaccard_results,
  .organism = ".*",
  .metric = "Jaccard index"
) +
  scale_fill_distiller(
    palette = "Greens",
    direction = 1
  )
plot
```


```
# ggsave(plot,
#   filename = "jaccard_all_species_mean.svg",
#   height = similarity_plot_height,
#   width = similarity_plot_width
# )
```


```
benchmark_jaccard_results |>
  mutate(
    model1 = model1 |> str_remove_all("\n"),
    model2 = model2 |> str_remove_all("\n")
  ) |>
  mutate(jaccard = unlist(value)) |>
  select(-jaccard_data, value) |>
  write_tsv("benchmark_jaccard_values.tsv")
```

## Seq similarity


```
sequence_similarity <- function(df) {
  A <- df$codons1 |> unlist()
  B <- df$codons2 |> unlist()
  if (length(A) != length(B)) {
    return("different length")
  } else if (is.null(A) | is.null(B)) {
    return(NA)
  } else {
    sum <- 0
    for (i in seq(1, length(A))) {
      if (A[i] == B[i]) {
        sum <- sum + 1
      }
    }
    sum <- (sum * 100) / length(A)
    return(sum)
  }
}

benchmark_sequence_similarity_results <- benchmark_sequence_data |>
  mutate(codons = sequence |>
    str_extract_all(pattern = "[:alpha:]{3}")) |>
  mutate(
    model1 = model, codons1 = codons,
    model2 = model, codons2 = codons
  ) |>
  nest(
    comparison1 = c(model1, codons1),
    comparison2 = c(model2, codons2)
  ) |>
  select(organism, protein, comparison1, comparison2) |>
  group_by(organism, protein) |>
  complete(comparison1, comparison2) |>
  unnest(c(comparison1, comparison2)) |>
  nest(seq_similarity_data = c(codons1, codons2)) |>
  mutate(value = map(seq_similarity_data, sequence_similarity))
```


#### Ec


```
plot <- plot_tile_similarity(
  .data_struc = benchmark_sequence_similarity_results,
  .organism = organism_levels[1],
  .metric = "sequence similarity"
) +
  scale_fill_distiller(
    palette = "Blues",
    direction = 1
  )
plot
```


```
# ggsave(plot,
#   filename = "seq_similarity_ecoli.svg",
#   height = similarity_plot_height,
#   width = similarity_plot_width
# )
```

#### Sc


```
plot <- plot_tile_similarity(
  .data_struc = benchmark_sequence_similarity_results,
  .organism = organism_levels[2],
  .metric = "sequence similarity"
) +
  scale_fill_distiller(
    palette = "Blues",
    direction = 1
  )
plot
```


```
# ggsave(plot,
#   filename = "seq_similarity_scerevisiae.svg",
#   height = similarity_plot_height,
#   width = similarity_plot_width
# )
```

#### At


```
plot <- plot_tile_similarity(
  .data_struc = benchmark_sequence_similarity_results,
  .organism = organism_levels[3],
  .metric = "sequence similarity"
) +
  scale_fill_distiller(
    palette = "Blues",
    direction = 1
  )
plot
```


```
# ggsave(plot,
#   filename = "seq_similarity_Athaliana.svg",
#   height = similarity_plot_height,
#   width = similarity_plot_width
# )
```

#### Mm


```
plot <- plot_tile_similarity(
  .data_struc = benchmark_sequence_similarity_results,
  .organism = organism_levels[4],
  .metric = "sequence similarity"
) +
  scale_fill_distiller(
    palette = "Blues",
    direction = 1
  )
plot
```


```
# ggsave(plot,
#   filename = "seq_similarity_mmusculus.svg",
#   height = similarity_plot_height,
#   width = similarity_plot_width
# )
```

#### Hs


```
plot <- plot_tile_similarity(
  .data_struc = benchmark_sequence_similarity_results,
  .organism = organism_levels[5],
  .metric = "sequence similarity"
) +
  scale_fill_distiller(
    palette = "Blues",
    direction = 1
  )
plot
```


```
# ggsave(plot,
#   filename = "seq_similarity_hsapiens.svg",
#   height = similarity_plot_height,
#   width = similarity_plot_width
# )
```

### all


```
plot <- plot_tile_similarity(
  .data_struc = benchmark_sequence_similarity_results,
  .organism = ".*",
  .metric = "sequence similarity"
) +
  scale_fill_distiller(
    palette = "Blues",
    direction = 1
  )
plot
```


```
# ggsave(plot,
#   filename = "seq_similarity_all_species_mean.svg",
#   height = similarity_plot_height,
#   width = similarity_plot_width
# )
```


```
benchmark_sequence_similarity_results |>
  mutate(
    model1 = model1 |> str_remove_all("\n"),
    model2 = model2 |> str_remove_all("\n")
  ) |>
  mutate(seq_similarity = unlist(value)) |>
  select(-seq_similarity_data, -value) |>
  write_tsv("benchmark_sequence_similarity.tsv")
```

## %minmax


```
benchmark_minmax_results <- benchmark_sequence_data |>
  select(organism, protein, model, sequence) |>
  mutate(species = ifelse(str_detect(organism, "coli"), "ecoli",
    ifelse(str_detect(organism, "cerevisiae"), "saccharomyces",
      ifelse(str_detect(organism, "thaliana"), "arabidopsis",
        ifelse(str_detect(organism, "musculus"), "mouse",
          ifelse(str_detect(organism, "sapiens"), "human", NA)
        )
      )
    )
  )) |>
  nest(data = c(sequence, species)) |>
  mutate(minmax = map(data, compute_minmax)) |>
  unnest(data) |>
  unnest(minmax) |>
  group_by(organism, model, protein) |>
  mutate(index = row_number())
```


### plot species


```
plot <- plot_line_minmax(
  .data_structure = benchmark_minmax_results,
  .organism = organism_levels[1],
  .limits = c(-1, 1.2),
  .ncol = 6
)

# ggsave(plot,
#   filename = "minmax_benchmark_Ecoli_w=18.svg",
#   height = 120,
#   width = 20,
#   limitsize = FALSE
# )
```


```
plot <- plot_line_minmax(
  .data_structure = benchmark_minmax_results,
  .organism = organism_levels[2],
  .limits = c(-1, 1.2),
  .ncol = 5
)

# ggsave(plot,
#   filename = "minmax_benchmark_Scerevisiae_w=18.svg",
#   height = 120,
#   width = 16,
#   limitsize = FALSE
# )
```


```
plot <- plot_line_minmax(
  .data_structure = benchmark_minmax_results,
  .organism = organism_levels[3],
  .limits = c(-1, 1.2),
  .ncol = 5
)

# ggsave(plot,
#   filename = "minmax_benchmark_Athaliana_w=18.svg",
#   height = 120,
#   width = 16,
#   limitsize = FALSE
# )
```


```
plot <- plot_line_minmax(
  .data_structure = benchmark_minmax_results,
  .organism = organism_levels[4],
  .limits = c(-1, 1.2),
  .ncol = 5
)

# ggsave(plot,
#   filename = "minmax_benchmark_Mmusculus_w=18.svg",
#   height = 120,
#   width = 16,
#   limitsize = FALSE
# )
```


```
plot <- plot_line_minmax(
  .data_structure = benchmark_minmax_results,
  .organism = organism_levels[5],
  .limits = c(-1, 1.2),
  .ncol = 5
)

# ggsave(plot,
#   filename = "minmax_benchmark_Hsapiens_w=18.svg",
#   height = 120,
#   width = 16,
#   limitsize = FALSE
# )
```


```
benchmark_minmax_results |>
  select(-species, -sequence) |>
  mutate(
    model = model |> str_remove_all("\n"),
    minmax = minmax |> round(digits = 2)
  ) |>
  pivot_wider(names_from = index, values_from = minmax) |>
  unite(col = "minmax", matches("\\d"), sep = ",") |>
  mutate(minmax = minmax |> str_remove_all(",NA")) |>
  write_tsv("benchmark_minmax_profiles.tsv")
```

## DTW


```
benchmark_DTW_results <- benchmark_minmax_results |>
  select(-species, -index, -sequence) |>
  group_by(organism, protein, model) |>
  nest(minmax = minmax) |>
  mutate(
    model1 = model, minmax1 = minmax,
    model2 = model, minmax2 = minmax
  ) |>
  nest(
    comparison1 = c(model1, minmax1),
    comparison2 = c(model2, minmax2)
  ) |>
  ungroup() |>
  select(organism, protein, comparison1, comparison2) |>
  group_by(organism, protein) |>
  complete(comparison1, comparison2) |>
  unnest(c(comparison1, comparison2)) |>
  nest(dtw_data = c(minmax1, minmax2)) |>
  mutate(dtw_object = map(dtw_data, compute_DTW)) |>
  mutate(
    distance = map(dtw_object, pluck("distance")),
    normalized_distance = map(dtw_object, pluck("normalizedDistance"))
  ) |>
  select(organism, protein, model1, model2, distance, normalized_distance) |>
  select(organism, description, model1, model2, dtw_object)

saveRDS(benchmark_DTW_results, "benchmark_DTW_results.rds")
```


```
benchmark_DTW_results <- readRDS("benchmark_DTW_results.rds")
```


#### Ec


```
plot <- plot_tile_similarity(
  .data_struc = benchmark_DTW_results |>
    mutate(value = normalized_distance),
  .organism = organism_levels[1],
  .metric = "DTW distance"
) +
  scale_fill_gradient(
    low = "#F7FEAD",
    high = "#01708B"
  )

plot
```


```
# ggsave(plot,
#   filename = "DTW_ecoli_benchmark.svg",
#   height = similarity_plot_height,
#   width = similarity_plot_width
# )
```

#### Sc


```
plot <- plot_tile_similarity(
  .data_struc = benchmark_DTW_results |> mutate(value = normalized_distance),
  .organism = organism_levels[2],
  .metric = "DTW distance"
) +
  scale_fill_gradient(
    low = "#F7FEAD",
    high = "#01708B"
  )

plot
```


```
# ggsave(plot,
#   filename = "DTW_scerevisiae_benchmark.svg",
#   height = similarity_plot_height,
#   width = similarity_plot_width
# )
```

#### At


```
plot <- plot_tile_similarity(
  .data_struc = benchmark_DTW_results |> mutate(value = normalized_distance),
  .organism = organism_levels[3],
  .metric = "DTW distance"
) +
  scale_fill_gradient(
    low = "#F7FEAD",
    high = "#01708B"
  )

plot
```


```
# ggsave(plot,
#   filename = "DTW_Athaliana_benchmark.svg",
#   height = similarity_plot_height,
#   width = similarity_plot_width
# )
```

#### Mm


```
plot <- plot_tile_similarity(
  .data_struc = benchmark_DTW_results |> mutate(value = normalized_distance),
  .organism = organism_levels[4],
  .metric = "DTW distance"
) +
  scale_fill_gradient(
    low = "#F7FEAD",
    high = "#01708B"
  )

plot
```


```
# ggsave(plot,
#   filename = "DTW_mmusculus_benchmark.svg",
#   height = similarity_plot_height,
#   width = similarity_plot_width
# )
```

#### Hs


```
plot <- plot_tile_similarity(
  .data_struc = benchmark_DTW_results |> mutate(value = normalized_distance),
  .organism = organism_levels[5],
  .metric = "DTW distance"
) +
  scale_fill_gradient(
    low = "#F7FEAD",
    high = "#01708B"
  )

plot
```


```
# ggsave(plot,
#   filename = "DTW_hsapiens_benchmark.svg",
#   height = similarity_plot_height,
#   width = similarity_plot_width
# )
```

#### all


```
plot <- plot_tile_similarity(
  .data_struc = benchmark_DTW_results |> mutate(value = normalized_distance),
  .organism = ".*",
  .metric = "DTW distance"
) +
  scale_fill_gradient(
    low = "#F7FEAD",
    high = "#01708B"
  )

plot
```


```
# ggsave(plot,
#   filename = "DTW_distances_between_models_for_benchmark_genes.svg",
#   height = similarity_plot_height,
#   width = similarity_plot_width
# )
```


```
benchmark_DTW_results |>
  mutate(
    model1 = model1 |> str_remove_all("\n"),
    model2 = model2 |> str_remove_all("\n")
  ) |>
  select(organism, protein, model1, model2, distance, normalized_distance) |>
  mutate(
    distance = unlist(distance),
    normalized_distance = unlist(normalized_distance)
  ) |>
  write_tsv("benchmark_DTW_minmax.tsv")
```

## Negative cis-elements


```
plot <- benchmark_sequence_data |>
  mutate(organism = factor(organism, levels = organism_levels)) |>
  ggplot(aes(x = model, y = as.numeric(nb_cis_element), color = model)) +
  geom_boxplot(color = "black", outlier.shape = NA) +
  geom_jitter(width = 0.2, height = 0.2, size = 1, alpha = 0.5) +
  scale_color_manual(values = model_colors[-1]) +
  stat_summary(
    fun.y = mean, fun.min = mean, fun.max = mean,
    geom = "point", color = "black", shape = 4, size = 4
  ) +
  scale_y_continuous(breaks = c(0, 2, 4, 6, 8, 10)) +
  facet_wrap(~organism, ncol = 5, scales = "free") +
  ylab("Number of cis regulatory element") +
  theme_bw() +
  theme(
    axis.title.x = element_blank(),
    axis.text.x = element_blank(),
    legend.title = element_blank(),
    plot.title = element_text(hjust = 0.5),
    strip.text = element_text(face = "italic"),
    legend.position = "none"
  )

plot
```


```
# ggsave(plot,
#   filename = "Cis_regulatory_elements_without_legend.svg",
#   height = 3,
#   width = 10
# )
```

## RNA fold


```
benchmark_RNA_folding_results <- benchmark_sequence_data |>
  mutate(RNA_fold_object = map(sequence, compute_RNA_fold)) |>
  select(organism, protein, model, sequence, RNA_fold_object)

saveRDS(benchmark_RNA_folding_results, "benchmark_RNA_folding.rds")
```


```
benchmark_RNA_folding_results <- readRDS("benchmark_RNA_folding.rds") |>
  mutate(
    organism = factor(organism, levels = organism_levels),
    mfe = map(RNA_fold_object, pluck("mfe")),
    mfe = as.numeric(mfe),
    length = str_count(sequence),
    mfe_normalized_by_length = mfe / length,
    GC_content = str_count(sequence, "G|C") / length
  )
```


#### mfe # length


```
plot <- benchmark_RNA_folding_results |>
  mutate(y_plot = mfe, x_plot = length) |>
  plot_smooth_model() +
  scale_color_manual(values = model_colors[-1]) +
  scale_fill_manual(values = model_colors[-1])
plot
```


```
# ggsave(plot,
#   filename = "RNA_folding_1_linear_relation_to_length_benchmark.svg",
#   height = 4,
#   width = 6
# )
```

#### normalized by species


```
plot <- benchmark_RNA_folding_results |>
  mutate(value = mfe_normalized_by_length) |>
  plot_jitter_species() +
  xlab("mfe / length")
plot
```


```
# ggsave(plot,
#   filename = "RNA_folding_3_species_separation_normalized_benchmark.svg",
#   height = 4,
#   width = 6
# )
```

#### GC by species


```
plot <- benchmark_RNA_folding_results |>
  mutate(value = GC_content) |>
  plot_jitter_species() +
  xlab("GC content")
plot
```


```
# ggsave(plot,
#   filename = "RNA_folding_4_species_separation_GC_content.svg",
#   height = 4,
#   width = 6
# )
```


```
benchmark_RNA_folding_results |>
  mutate(model = model |> str_remove_all("\n")) |>
  select(organism, protein, mfe, length, mfe_normalized_by_length, GC_content) |>
  write_tsv("benchmark_RNA_fold.tsv")
```


Genomic and model generated sequences should be recovered on
Zenodo.

# genome sequences


```
# import natural and optimized sequences
codon_across_genomes <- genomic_sequences |> 
  mutate("Natural"=str_extract_all(original_sequence, "[A-Z]{3}"),
         "Base \n model"=str_extract_all(base_model, "[A-Z]{3}"),
         "Fine \n tuned"=str_extract_all(fine_tuned, "[A-Z]{3}")) |> 
  pivot_longer(cols=c("Natural", "Base \n model", "Fine \n tuned"),
               names_to="model",
               values_to = "codon") |> 
  unnest_longer(codon) |> 
  inner_join(codon_aa_table,
             by=c("codon"="codon")) |> 
  mutate(model=factor(model,
                      levels=c("Natural", "Base \n model", "Fine \n tuned")))
rm(genomic_sequences)
```


## codon distribution


```
codon_across_genomes |>
  mutate(model=model |> str_remove_all("\n")) |> 
  group_by(organism, model, token) |> 
  summarise(codon_count=n()) |> 
  write_tsv("genome_sequences_codon_counts.tsv")
```


```
plot1 <- codon_across_genomes |> 
  filter(organism=="Escherichia coli") +
  ggplot(aes(y = token, fill=model)) +
    geom_bar() +
  scale_fill_manual(values=model_colors[1:3])+
      scale_x_continuous(breaks = c(0, 100000))+
    facet_wrap(~model, ncol=3) +
    theme_bw() +
    ylab("Codons") +
    xlab("") +
    ggtitle("E. coli") +
    theme(plot.title = element_text(hjust = 0.5),
          axis.title.y=element_text(face="italic"))
plot2 <- codon_across_genomes |> 
  filter(organism=="Saccharomyces cerevisiae") |> 
  ggplot(aes(y = token, fill=model)) +
    geom_bar() +
  scale_fill_manual(values=model_colors[1:3])+
      scale_x_continuous(breaks = c(0, 200000))+
    facet_wrap(~model, ncol=3) +
    theme_bw() +
    ylab("Codons") +
    xlab("") +
    ggtitle("S. cerevisiae") +
    theme(plot.title = element_text(hjust = 0.5),
          axis.title.y=element_text(face="italic"))
plot3 <- codon_across_genomes |> 
  filter(organism=="Arabidopsis thaliana") |> 
  ggplot(aes(y = token, fill=model)) +
    geom_bar() +
  scale_fill_manual(values=model_colors[1:3])+
      scale_x_continuous(breaks = c(0, 1000000))+
    facet_wrap(~model, ncol=3) +
    theme_bw() +
    ylab("Codons") +
    xlab("") +
    ggtitle("A. thaliana") +
    theme(plot.title = element_text(hjust = 0.5),
          axis.title.y=element_text(face="italic"))
plot4 <- codon_across_genomes |> 
  filter(organism=="Mus musculus") |> 
  ggplot(aes(y = token, fill=model)) +
    geom_bar() +
  scale_fill_manual(values=model_colors[1:3])+
      scale_x_continuous(breaks = c(0,3000000))+
    facet_wrap(~model, ncol=3) +
    theme_bw() +
    ylab("Codons") +
    xlab("") +
    ggtitle("M. musculus") +
    theme(plot.title = element_text(hjust = 0.5),
          axis.title.y=element_text(face="italic"))
plot5 <- codon_across_genomes |> 
  filter(organism=="Homo sapiens") |> 
  ggplot(aes(y = token, fill=model)) +
    geom_bar() +
  scale_fill_manual(values=model_colors[1:3])+
      scale_x_continuous(breaks = c(0, 5000000))+
    facet_wrap(~model, ncol=3) +
    theme_bw() +
    ylab("Codons") +
    xlab("") +
    ggtitle("H. sapiens") +
    theme(plot.title = element_text(hjust = 0.5),
          axis.title.y=element_text(face="italic"))
```


```
plot=plot1+plot2+plot3+plot4+plot5+
  plot_layout(
      guides = "collect",
      axes = "collect",
      ncol=5
    ) +
    plot_annotation(
      title = paste0("Codon distribution across genomic sequences"),
      theme = theme(plot.title = element_text(hjust = 0.5)) &
        theme(plot.title = element_text(size = 18))
    )
```


```
plot
```

# Session info


```
print(sessionInfo(), locale = FALSE)
```


```
R version 4.4.1 (2024-06-14 ucrt)
Platform: x86_64-w64-mingw32/x64
Running under: Windows 11 x64 (build 22631)

Matrix products: default


attached base packages:
[1] stats     graphics  grDevices utils     datasets  methods   base     

other attached packages:
 [1] XNAString_1.12.0    BiocManager_1.30.25 dtw_1.23-1         
 [4] proxy_0.4-27        kodonz_0.1.1        devtools_2.4.5     
 [7] usethis_3.0.0       svglite_2.1.3       readxl_1.4.3       
[10] rstatix_0.7.2       patchwork_1.3.0     RColorBrewer_1.1-3 
[13] ggpubr_0.6.0        ggnewscale_0.5.0    lubridate_1.9.3    
[16] forcats_1.0.0       stringr_1.5.1       dplyr_1.1.4        
[19] purrr_1.0.2         readr_2.1.5         tidyr_1.3.1        
[22] tibble_3.2.1        ggplot2_3.5.1       tidyverse_2.0.0    

loaded via a namespace (and not attached):
  [1] rstudioapi_0.16.0           jsonlite_1.8.8             
  [3] magrittr_2.0.3              farver_2.1.2               
  [5] rmarkdown_2.28              ragg_1.3.3                 
  [7] fs_1.6.4                    BiocIO_1.14.0              
  [9] zlibbioc_1.50.0             vctrs_0.6.5                
 [11] memoise_2.0.1               Rsamtools_2.20.0           
 [13] RCurl_1.98-1.16             polynom_1.4-1              
 [15] htmltools_0.5.8.1           S4Arrays_1.4.1             
 [17] curl_5.2.3                  broom_1.0.7                
 [19] cellranger_1.1.0            SparseArray_1.4.8          
 [21] Formula_1.2-5               sass_0.4.9                 
 [23] parallelly_1.38.0           bslib_0.8.0                
 [25] htmlwidgets_1.6.4           cachem_1.1.0               
 [27] GenomicAlignments_1.40.0    mime_0.12                  
 [29] lifecycle_1.0.4             pkgconfig_2.0.3            
 [31] Matrix_1.7-0                R6_2.5.1                   
 [33] fastmap_1.2.0               GenomeInfoDbData_1.2.12    
 [35] MatrixGenerics_1.16.0       future_1.34.0              
 [37] shiny_1.9.1                 digest_0.6.37              
 [39] colorspace_2.1-1            S4Vectors_0.42.1           
 [41] pkgload_1.4.0               textshaping_0.4.0          
 [43] GenomicRanges_1.56.1        labeling_0.4.3             
 [45] fansi_1.0.6                 timechange_0.3.0           
 [47] mgcv_1.9-1                  httr_1.4.7                 
 [49] abind_1.4-8                 compiler_4.4.1             
 [51] remotes_2.5.0               bit64_4.0.5                
 [53] withr_3.0.1                 backports_1.5.0            
 [55] BiocParallel_1.38.0         carData_3.0-5              
 [57] pkgbuild_1.4.4              ggsignif_0.6.4             
 [59] DelayedArray_0.30.1         sessioninfo_1.2.2          
 [61] rjson_0.2.23                tools_4.4.1                
 [63] formattable_0.2.1           httpuv_1.6.15              
 [65] future.apply_1.11.2         glue_1.7.0                 
 [67] restfulr_0.0.15             nlme_3.1-164               
 [69] promises_1.3.0              grid_4.4.1                 
 [71] generics_0.1.3              gtable_0.3.5               
 [73] BSgenome_1.72.0             tzdb_0.4.0                 
 [75] data.table_1.16.0           hms_1.1.3                  
 [77] car_3.1-3                   utf8_1.2.4                 
 [79] XVector_0.44.0              BiocGenerics_0.50.0        
 [81] pillar_1.9.0                vroom_1.6.5                
 [83] later_1.3.2                 splines_4.4.1              
 [85] lattice_0.22-6              bit_4.0.5                  
 [87] rtracklayer_1.64.0          tidyselect_1.2.1           
 [89] Biostrings_2.72.1           miniUI_0.1.1.1             
 [91] knitr_1.48                  IRanges_2.38.1             
 [93] SummarizedExperiment_1.34.0 stats4_4.4.1               
 [95] xfun_0.47                   Biobase_2.64.0             
 [97] matrixStats_1.4.0           stringi_1.8.4              
 [99] UCSC.utils_1.0.0            yaml_2.3.10                
[101] evaluate_1.0.0              codetools_0.2-20           
[103] cli_3.6.3                   systemfonts_1.1.0          
[105] xtable_1.8-4                jquerylib_0.1.4            
[107] munsell_0.5.1               Rcpp_1.0.13                
[109] GenomeInfoDb_1.40.1         globals_0.16.3             
[111] XML_3.99-0.17               parallel_4.4.1             
[113] ellipsis_0.3.2              profvis_0.4.0              
[115] urlchecker_1.0.1            bitops_1.0-8               
[117] listenv_0.9.1               pwalign_1.0.0              
[119] scales_1.3.0                crayon_1.5.3               
[121] rlang_1.1.4
```

LS0tDQp0aXRsZTogIkNvZG9uVHJhbnNmb3JtZXI6IGEgbXVsdGlzcGVjaWVzIGNvZG9uIG9wdGltaXplciB1c2luZyBjb250ZXh0LWF3YXJlIG5ldXJhbCBuZXR3b3JrcyINCm91dHB1dDogDQogIGh0bWxfbm90ZWJvb2s6DQogICAgdG9jOiB0cnVlDQogICAgdG9jX2RlcHRoOiAyDQogICAgY29kZV9mb2xkaW5nOiBoaWRlDQotLS0NCg0KYGBge3Igc2V0dXAsIGluY2x1ZGU9RkFMU0V9DQppZiAoIXJlcXVpcmUoInRpZHl2ZXJzZSIpKSB7DQogIGluc3RhbGwucGFja2FnZXMoInRpZHl2ZXJzZSIpDQp9DQpsaWJyYXJ5KHRpZHl2ZXJzZSkNCg0KIyBnZ3Bsb3QgcmVsYXRlZCBwYWNrYWdlcw0KaWYgKCFyZXF1aXJlKCJnZ25ld3NjYWxlIikpIHsNCiAgaW5zdGFsbC5wYWNrYWdlcygiZ2duZXdzY2FsZSIpDQp9DQpsaWJyYXJ5KGdnbmV3c2NhbGUpDQoNCmlmICghcmVxdWlyZSgiZ2dwdWJyIikpIHsNCiAgaW5zdGFsbC5wYWNrYWdlcygiZ2dwdWJyIikNCn0NCmxpYnJhcnkoZ2dwdWJyKQ0KDQppZiAoIXJlcXVpcmUoIlJDb2xvckJyZXdlciIpKSB7DQogIGluc3RhbGwucGFja2FnZXMoIlJDb2xvckJyZXdlciIpDQp9DQpsaWJyYXJ5KFJDb2xvckJyZXdlcikNCg0KaWYgKCFyZXF1aXJlKCJwYXRjaHdvcmsiKSkgew0KICBpbnN0YWxsLnBhY2thZ2VzKCJwYXRjaHdvcmsiKQ0KfQ0KbGlicmFyeShwYXRjaHdvcmspDQoNCiMgc3RhdGlzdGljYWwgdGVzdGluZw0KaWYgKCFyZXF1aXJlKCJyc3RhdGl4IikpIHsNCiAgaW5zdGFsbC5wYWNrYWdlcygicnN0YXRpeCIpDQp9DQpsaWJyYXJ5KHJzdGF0aXgpDQoNCiMgcmVhZCBleGNlbCBmaWxlcw0KaWYgKCFyZXF1aXJlKCJyZWFkeGwiKSkgew0KICBpbnN0YWxsLnBhY2thZ2VzKCJyZWFkeGwiKQ0KfQ0KbGlicmFyeShyZWFkeGwpDQoNCiMgaGFuZGxlIHN2ZyBmb3JtYXQNCmlmICghcmVxdWlyZSgic3ZnbGl0ZSIpKSB7DQogIGluc3RhbGwucGFja2FnZXMoInN2Z2xpdGUiKQ0KfQ0KbGlicmFyeShzdmdsaXRlKQ0KDQojIGluc3RhbGwgZnJvbSBnaXRodWINCmlmICghcmVxdWlyZSgiZGV2dG9vbHMiKSkgew0KICBpbnN0YWxsLnBhY2thZ2VzKCJkZXZ0b29scyIpDQp9DQoNCiMgY29tcHV0ZSAlbWlubWF4DQppZiAoIXJlcXVpcmUoImtvZG9ueiIpKSB7DQogIGRldnRvb2xzOjppbnN0YWxsX2dpdGh1YigiSFZvbHRCYi9rb2RvbnoiKQ0KfQ0KbGlicmFyeShrb2RvbnopDQoNCiMgY29tcHV0ZSBEVFcNCmlmICghcmVxdWlyZSgiZHR3IikpIHsNCiAgaW5zdGFsbC5wYWNrYWdlcygiZHR3IikNCn0NCmxpYnJhcnkoZHR3KQ0KDQojIGluc3RhbGwgZnJvbSBiaW9jb25kdWN0b3INCmlmICghcmVxdWlyZSgiQmlvY01hbmFnZXIiKSkgew0KICBpbnN0YWxsLnBhY2thZ2VzKCJCaW9jTWFuYWdlciIpDQp9DQoNCiMgY29tcHV0ZSBSTkFmb2xkDQppZiAoIXJlcXVpcmUoIlhOQVN0cmluZyIpKSB7DQogIEJpb2NNYW5hZ2VyOjppbnN0YWxsKCJYTkFTdHJpbmciKQ0KfQ0KbGlicmFyeShYTkFTdHJpbmcpDQpgYGANCg0KVGhpcyBjb2RlIHJlcHJvZHVjZXMgZ2dwbG90IGZpZ3VyZXMgaW4gIkNvZG9uVHJhbnNmb3JtZXI6IGEgbXVsdGlzcGVjaWVzIGNvZG9uIG9wdGltaXplciB1c2luZyBjb250ZXh0LWF3YXJlIG5ldXJhbCBuZXR3b3JrcyIuDQoNCkl0IHJlYWRzIG5hdHVyYWwgYW5kIGJlbmNobWFyayBETkEgc2VxdWVuY2VzIGZyb20gc3VwcGxlbWVudGFyeSBmaWxlcyAxIGFuZCAyICh3aGljaCBtdXN0IGJlIHBsYWNlZCBpbiB0aGUgc2FtZSBmb2xkZXIpIGFuZCBjcmVhdGUgZmlndXJlcy4gDQoNCkdlbm9taWMgc2VxdWVuY2VzLCBEVFcgZGlzdGFuY2VzIGFuZCBSTkEgZm9sZGluZyByZXN1bHRzIGFyZSBzYXZlZCBhcyBSRFMgb2JqZWN0cyBhbmQgdGhlIGNvcnJlc3BvbmRpbmcgY2h1bmtzIG9mIGNvZGUgYXJlIGZsYWdnZWQgYXMgImV2YWwgPSBGQUxTRSIuDQoNCiMgZ2VuZXJpYyB2YXJpYWJsZXMgYW5kIHBsb3R0aW5nIGZ1bmN0aW9ucw0KDQpgYGB7cn0NCiMgbW9kZWwgbmFtZXMgbWF0Y2hpbmcgaW4gZGlmZmVyZW50IGZpbGVzDQptb2RlbF9wYXR0ZXJucyA8LSBjKA0KICAiYXR1cmFsIiwNCiAgImJhc2V8cHJldHJhaW4iLA0KICAiZmluZSIsDQogICJUd2lzdHx0d2lzdCIsDQogICJHZW5ld2l6fGdlbmV3aXoiLA0KICAiSURUfGlkdCIsDQogICJJQ09SfGljb3IiDQopDQoNCiMgbW9kZWwgbmFtaW5nIG9uIGZpZ3VyZXMNCm1vZGVsX2xldmVscyA8LSBjKA0KICAiTmF0dXJhbCIsDQogICJCYXNlIFxuIENvZG9uLSBcbiBUcmFuc2Zvcm1lciIsDQogICJGaW5lLXR1bmVkIFxuIENvZG9uLSBcbiBUcmFuc2Zvcm1lciIsDQogICJUd2lzdCIsDQogICJHZW5ld2l6IiwNCiAgIklEVCIsDQogICJJQ09SIg0KKQ0KDQojIG1vZGVsIGNvbG9ycw0KbW9kZWxfY29sb3JzIDwtIGMoDQogICJibGFjayIsDQogICIjRjk5MDlGIiwNCiAgIiNGNDAwM0YiLA0KICAiI0E4MjFERiIsDQogICIjMDJGRkEyIiwNCiAgIiMyMDc0REYiLA0KICAiI0ZGQTQwMCINCikNCg0KIyBzcGVjaWVzIGNvbG9yDQpzcGVjaWVzX2NvbG9yIDwtIGJyZXdlci5wYWwoOSwgIlNldDEiKVtjKDcsIDQsIDMsIDksIDgpXQ0KDQojIGNvbXB1dGUgbWlubWF4DQpjb21wdXRlX21pbm1heCA8LSBmdW5jdGlvbihkZikgew0KICBzZXEgPC0ga29kb256Ojpsb2FkLmZhc3RhKHggPSBkZiRzZXF1ZW5jZSkNCiAgcmVzIDwtIGtvZG9uejo6cG1pbm1heChzZXFbWzFdXSwNCiAgICAjIHdpbmRvdyBsZW5ndGgNCiAgICB6ID0gMTgsDQogICAgIyBzdGFuZGFyZCBjb2RvbiB0YWJsZQ0KICAgIHkgPSAwLA0KICAgICMgc3BlY2llcyBmb3Igd2hpY2ggY3V0IGlzIG92ZXJpZGVkDQogICAgc3BwID0gZGYkc3BlY2llcw0KICApDQp9DQoNCiMgY29tcHV0ZSBEeW5hbWljIHRpbWUgd2FycGluZyBkaXN0YW5jZXMNCmNvbXB1dGVfRFRXIDwtIGZ1bmN0aW9uKGRmKSB7DQogIG1pbm1heDEgPC0gZGYgfD4NCiAgICBwdWxsKG1pbm1heDEpIHw+DQogICAgdW5saXN0KCkNCiAgbWlubWF4MiA8LSBkZiB8Pg0KICAgIHB1bGwobWlubWF4MikgfD4NCiAgICB1bmxpc3QoKQ0KICBkdHcobWlubWF4MSwgbWlubWF4MikNCn0NCg0KIyBjb21wdXRlIFJOQSBzdHJ1Y3R1cmVzDQpjb21wdXRlX1JOQV9mb2xkIDwtIGZ1bmN0aW9uKHNlcXVlbmNlKSB7DQogICMgUHJlZGljdCBmb2xkaW5nIGFuZCBEckcNCiAgWE5BU3RyaW5nOjpwcmVkaWN0TWZlU3RydWN0dXJlKA0KICAgICMgQ29udmVydCB0byBYTkEgb2JqZWN0DQogICAgWE5BU3RyaW5nOjpYTkFTdHJpbmcoDQogICAgICBiYXNlID0NCiAgICAgICMgU3RvcmUgaW4gUk5BIG9iamVjdA0KICAgICAgICBCaW9zdHJpbmdzOjpSTkFTdHJpbmcoDQogICAgICAgICAgIyBjb252ZXJ0IEROQSB0byBSTkENCiAgICAgICAgICB4ID0gc2VxdWVuY2UgfD4gc3RyX3JlcGxhY2VfYWxsKCJUIiwgIlUiKQ0KICAgICAgICApDQogICAgKQ0KICApDQp9DQoNCiMgcGxvdCAlbWlubWF4IHByb2ZpbGVzDQpwbG90X2xpbmVfbWlubWF4IDwtIGZ1bmN0aW9uKC5kYXRhX3N0cnVjdHVyZSA9IE5BLA0KICAgICAgICAgICAgICAgICAgICAgICAgICAgICAub3JnYW5pc20gPSBOQSwNCiAgICAgICAgICAgICAgICAgICAgICAgICAgICAgLmxpbWl0cyA9IE5BLA0KICAgICAgICAgICAgICAgICAgICAgICAgICAgICAubmNvbCA9IE5BKSB7DQogIC5kYXRhX3N0cnVjdHVyZSB8Pg0KICAgIG11dGF0ZSgNCiAgICAgIG1vZGVsID0gZmFjdG9yKG1vZGVsLCBsZXZlbHMgPSBtb2RlbF9sZXZlbHMpLA0KICAgICAgbmFtZSA9IGZhY3RvcihpZmVsc2UoImRlc2NyaXB0aW9uIiAlaW4lIGNvbG5hbWVzKC5kYXRhX3N0cnVjdHVyZSksDQogICAgICAgIGRlc2NyaXB0aW9uLA0KICAgICAgICBwcm90ZWluDQogICAgICApKQ0KICAgICkgfD4NCiAgICBmaWx0ZXIoc3RyX2RldGVjdChvcmdhbmlzbSwgLm9yZ2FuaXNtKSkgfD4NCiAgICBnZ3Bsb3QoYWVzKA0KICAgICAgeSA9IG1pbm1heCwNCiAgICAgIHggPSBpbmRleCwNCiAgICAgIGNvbG9yID0gbW9kZWwNCiAgICApKSArDQogICAgZ2VvbV9saW5lKGxpbmV3aWR0aCA9IDAuNSkgKw0KICAgIGxpbXMoeSA9IC5saW1pdHMpICsNCiAgICAjIHVzZSBjb2xvcnMgZm9yIG1vZGVscyBwcmVzZW50IGluIGRhdGENCiAgICBzY2FsZV9jb2xvcl9tYW51YWwodmFsdWVzID0gbW9kZWxfY29sb3JzW21vZGVsX2xldmVscyAlaW4lIC5kYXRhX3N0cnVjdHVyZSRtb2RlbF0pICsNCiAgICBmYWNldF93cmFwKH4gbmFtZSArIG1vZGVsLCBuY29sID0gLm5jb2wsIHNjYWxlcyA9ICJmcmVlX3giKSArDQogICAgZ2d0aXRsZShwYXN0ZTAoIm1pbm1heCBzZXF1ZW5jZXMgLSAiLCAub3JnYW5pc20pKSArDQogICAgdGhlbWUocGxvdC50aXRsZSA9IGVsZW1lbnRfdGV4dChoanVzdCA9IDAuNSkpICsNCiAgICBndWlkZXMoY29sb3IgPSAibm9uZSIpICsNCiAgICB0aGVtZV9idygpDQp9DQoNCiMgZm9yIHNpbWlsYXJpdHkgcGxvdHMNCnNpbWlsYXJpdHlfcGxvdF9oZWlnaHQgPC0gNg0Kc2ltaWxhcml0eV9wbG90X3dpZHRoIDwtIDgNCg0KIyBwbG90IHNpbWlsYXJpdHkgbWV0cmljcw0KcGxvdF90aWxlX3NpbWlsYXJpdHkgPC0gZnVuY3Rpb24oLmRhdGFfc3RydWMgPSBOQSwNCiAgICAgICAgICAgICAgICAgICAgICAgICAgICAgICAgIC5tZXRyaWMgPSBOQSwNCiAgICAgICAgICAgICAgICAgICAgICAgICAgICAgICAgIC5vcmdhbmlzbSA9IE5BKSB7DQogICMgYWRhcHQgdGV4dCBzaXplIHRvIHRoZSBudW1iZXIgb2YgbW9kZWxzDQogIG5iX2NhdGVnb3JpZXMgPC0gbGVuZ3RoKC5kYXRhX3N0cnVjJG1vZGVsMiB8PiB1bmlxdWUoKSkNCiAgZGF0YSA8LSAuZGF0YV9zdHJ1YyB8Pg0KICAgIGZpbHRlcihzdHJfZGV0ZWN0KG9yZ2FuaXNtLCAub3JnYW5pc20pKSB8Pg0KICAgIG11dGF0ZSgNCiAgICAgIG1vZGVsMSA9IGZhY3Rvcihtb2RlbDEsIGxldmVscyA9IHJldihtb2RlbF9sZXZlbHMpKSwNCiAgICAgIG1vZGVsMiA9IGZhY3Rvcihtb2RlbDIsIGxldmVscyA9IHJldihsZXZlbHMobW9kZWwxKSkpDQogICAgKSB8Pg0KICAgIGdyb3VwX2J5KG1vZGVsMSwgbW9kZWwyKSB8Pg0KICAgIHN1bW1hcmlzZSgNCiAgICAgIG1lYW5fbWV0cmljID0gbWVhbihhcy5udW1lcmljKHZhbHVlKSksDQogICAgICBzZF9tZXRyaWMgPSBzZChhcy5udW1lcmljKHZhbHVlKSksDQogICAgICAuZ3JvdXBzID0gImRyb3AiDQogICAgKSB8Pg0KICAgIG11dGF0ZShkaWFnb25hbCA9IGZhY3RvcihpZmVsc2UobW9kZWwxID09IG1vZGVsMiwgInllcyIsICJubyIpKSkgfD4NCiAgICAjIGZpbHRlciBvbmx5IHRoZSBib3R0b20gdHJpYW5nbGUgb2YgdGhlIGhlYXRtYXANCiAgICBtdXRhdGUobW9kZWwxX25iID0gYXMubnVtZXJpYyhtb2RlbDEpKSB8Pg0KICAgIG11dGF0ZShtb2RlbDJfbmIgPSBhcy5udW1lcmljKG1vZGVsMikpIHw+DQogICAgZmlsdGVyKChtb2RlbDJfbmIgKyBtb2RlbDFfbmIpIDw9IGxlbmd0aChtb2RlbF9sZXZlbHMpICsgMSkgfD4NCiAgICBnZ3Bsb3QoYWVzKHggPSBtb2RlbDEsIHkgPSBtb2RlbDIsIGZpbGwgPSBtZWFuX21ldHJpYykpICsNCiAgICBnZW9tX3RpbGUoKSArDQogICAgIyBmb3IgZGlhZ29uYWwsIHRleHQgPSBjZW50ZXJlZCBtZWFuDQogICAgZ2VvbV90ZXh0KA0KICAgICAgZGF0YSA9IH4gc3Vic2V0KC4sIGRpYWdvbmFsID09ICJ5ZXMiKSwNCiAgICAgIGFlcyhsYWJlbCA9IHBhc3RlMCgNCiAgICAgICAgcm91bmQobWVhbl9tZXRyaWMsIGRpZ2l0cyA9IDIpDQogICAgICApKSwNCiAgICAgIGNvbG9yID0gImJsYWNrIiwNCiAgICAgIHNpemUgPSAzNSAvIG5iX2NhdGVnb3JpZXMNCiAgICApICsNCiAgICAjIGZvciBvdGhlcnMsIHVwcGVyIHRleHQgPSBtZWFuDQogICAgZ2VvbV90ZXh0KA0KICAgICAgZGF0YSA9IH4gc3Vic2V0KC4sIGRpYWdvbmFsID09ICJubyIpLA0KICAgICAgYWVzKGxhYmVsID0gcGFzdGUwKA0KICAgICAgICByb3VuZChtZWFuX21ldHJpYywgZGlnaXRzID0gMiksDQogICAgICAgICJcbiINCiAgICAgICkpLA0KICAgICAgY29sb3IgPSAiYmxhY2siLA0KICAgICAgc2l6ZSA9IDM1IC8gbmJfY2F0ZWdvcmllcw0KICAgICkgKw0KICAgICMgZm9yIG90aGVycywgbG93ZXIgdGV4dCA9IHNkDQogICAgZ2VvbV90ZXh0KA0KICAgICAgZGF0YSA9IH4gc3Vic2V0KC4sIGRpYWdvbmFsID09ICJubyIpLA0KICAgICAgYWVzKGxhYmVsID0gcGFzdGUwKA0KICAgICAgICAiXG4gKy8tICIsDQogICAgICAgIHJvdW5kKHNkX21ldHJpYywgZGlnaXRzID0gMikNCiAgICAgICkpLA0KICAgICAgY29sb3IgPSAiYmxhY2siLA0KICAgICAgc2l6ZSA9IDI1IC8gbmJfY2F0ZWdvcmllcw0KICAgICkgKw0KICAgIGxhYnMoDQogICAgICB4ID0gIiIsDQogICAgICB5ID0gIiIsDQogICAgICB0aXRsZSA9IHBhc3RlMCgubWV0cmljLCAiIGJldHdlZW4gbW9kZWxzIGZvciAiLCAub3JnYW5pc20sICIgc2VxdWVuY2VzIiksDQogICAgICBmaWxsID0gIk1lYW4iDQogICAgKSArDQogICAgdGhlbWVfY2xhc3NpYygpICsNCiAgICB0aGVtZSgNCiAgICAgIHBsb3QudGl0bGUgPSBlbGVtZW50X3RleHQoaGp1c3QgPSAwLjUsIHNpemUgPSAxNCksDQogICAgICBsZWdlbmQua2V5LmhlaWdodCA9IHVuaXQoMSwgImNtIikNCiAgICApDQp9DQoNCnBsb3Rfaml0dGVyX3NwZWNpZXMgPC0gZnVuY3Rpb24oLmRhdGEpIHsNCiAgLmRhdGEgfD4NCiAgICBnZ3Bsb3QoYWVzKHggPSB2YWx1ZSwgeSA9IG1vZGVsLCBjb2xvciA9IG9yZ2FuaXNtKSkgKw0KICAgIGdlb21faml0dGVyKHNpemUgPSAxLCBhbHBoYSA9IDAuNywgd2lkdGggPSAwKSArDQogICAgc2NhbGVfY29sb3JfbWFudWFsKHZhbHVlcyA9IHNwZWNpZXNfY29sb3IpICsNCiAgICB0aGVtZV9idygpICsNCiAgICB0aGVtZShsZWdlbmQudGl0bGUgPSBlbGVtZW50X2JsYW5rKCkpICsNCiAgICBndWlkZXMoY29sb3VyID0gZ3VpZGVfbGVnZW5kKG92ZXJyaWRlLmFlcyA9IGxpc3Qoc2l6ZSA9IDMpKSkNCn0NCg0KcGxvdF9zbW9vdGhfbW9kZWwgPC0gZnVuY3Rpb24oLmRhdGEpIHsNCiAgLmRhdGEgfD4NCiAgICBnZ3Bsb3QoYWVzKHkgPSB5X3Bsb3QsIHggPSB4X3Bsb3QsIGNvbG9yID0gbW9kZWwpKSArDQogICAgZ2VvbV9wb2ludChzaXplID0gMSwgYWxwaGEgPSAwLjQpICsNCiAgICBnZW9tX3Ntb290aChhZXMoY29sb3IgPSBtb2RlbCwgZmlsbCA9IG1vZGVsKSwgbGluZXdpZHRoID0gMC41LCBhbHBoYSA9IDAuMywgbWV0aG9kID0gImxtIiwgc2UgPSBUUlVFLCBmb3JtdWxhID0gInkgfiB4IikgKw0KICAgIHRoZW1lX2J3KCkgKw0KICAgIHRoZW1lKA0KICAgICAgc3RyaXAudGV4dCA9IGVsZW1lbnRfYmxhbmsoKSwNCiAgICAgIGxlZ2VuZC5rZXkuc3BhY2luZy55ID0gdW5pdCgwLjIsICJjbSIpLA0KICAgICAgbGVnZW5kLmtleS53aWR0aCA9IHVuaXQoMSwgImNtIiksDQogICAgICBsZWdlbmQua2V5LmhlaWdodCA9IHVuaXQoMSwgImNtIiksDQogICAgICBzdHJpcC5iYWNrZ3JvdW5kID0gZWxlbWVudF9ibGFuaygpLA0KICAgICAgbGVnZW5kLnRpdGxlID0gZWxlbWVudF9ibGFuaygpDQogICAgKSArDQogICAgZ3VpZGVzKGNvbG9yID0gIm5vbmUiKSArDQogICAgZ3VpZGVzKGZpbGwgPSBndWlkZV9sZWdlbmQob3ZlcnJpZGUuYWVzID0gbGlzdChzaGFwZSA9IDIxLCBjb2xvdXIgPSBOQSkpKQ0KfQ0KDQojIDYxIGNvZG9ucyAoMjAgYW1pbm8gYWNpZHMpICsgMyBTVE9QIGNvZG9ucw0KY29kb25fYWFfdGFibGUgPC0gZHBseXI6OnRpYmJsZSgNCiAgdG9rZW4gPSBjKA0KICAgICJNX0FURyIsDQogICAgIkFfR0NHIiwNCiAgICAiQV9HQ0MiLA0KICAgICJBX0dDQSIsDQogICAgIkFfR0NUIiwNCiAgICAiQ19UR0MiLA0KICAgICJDX1RHVCIsDQogICAgIkRfR0FUIiwNCiAgICAiRF9HQUMiLA0KICAgICJFX0dBQSIsDQogICAgIkVfR0FHIiwNCiAgICAiRl9UVFQiLA0KICAgICJGX1RUQyIsDQogICAgIkdfR0dDIiwNCiAgICAiR19HR1QiLA0KICAgICJHX0dHRyIsDQogICAgIkdfR0dBIiwNCiAgICAiSF9DQVQiLA0KICAgICJIX0NBQyIsDQogICAgIklfQVRUIiwNCiAgICAiSV9BVEMiLA0KICAgICJJX0FUQSIsDQogICAgIktfQUFBIiwNCiAgICAiS19BQUciLA0KICAgICJMX0NURyIsDQogICAgIkxfVFRBIiwNCiAgICAiTF9UVEciLA0KICAgICJMX0NUQyIsDQogICAgIkxfQ1RUIiwNCiAgICAiTF9DVEEiLA0KICAgICJOX0FBQyIsDQogICAgIk5fQUFUIiwNCiAgICAiUF9DQ0ciLA0KICAgICJQX0NDQSIsDQogICAgIlBfQ0NUIiwNCiAgICAiUF9DQ0MiLA0KICAgICJRX0NBRyIsDQogICAgIlFfQ0FBIiwNCiAgICAiUl9DR0MiLA0KICAgICJSX0NHVCIsDQogICAgIlJfQ0dHIiwNCiAgICAiUl9DR0EiLA0KICAgICJSX0FHQSIsDQogICAgIlJfQUdHIiwNCiAgICAiU19BR0MiLA0KICAgICJTX1RDRyIsDQogICAgIlNfVENDIiwNCiAgICAiU19BR1QiLA0KICAgICJTX1RDVCIsDQogICAgIlNfVENBIiwNCiAgICAiVF9BQ0MiLA0KICAgICJUX0FDRyIsDQogICAgIlRfQUNUIiwNCiAgICAiVF9BQ0EiLA0KICAgICJWX0dURyIsDQogICAgIlZfR1RUIiwNCiAgICAiVl9HVEMiLA0KICAgICJWX0dUQSIsDQogICAgIllfVEFUIiwNCiAgICAiWV9UQUMiLA0KICAgICJXX1RHRyIsDQogICAgIlNUT1BfVEFBIiwNCiAgICAiU1RPUF9UR0EiLA0KICAgICJTVE9QX1RBRyINCiAgKQ0KKSB8Pg0KICBtdXRhdGUoDQogICAgY29kb24gPSBzdHJfZXh0cmFjdCh0b2tlbiwgIltBLVpdezN9JCIpLA0KICAgIGFhID0gc3RyX2V4dHJhY3QodG9rZW4sICJeW0EtWl0rIiksDQogICAgdG9rZW4gPSBmYWN0b3IodG9rZW4sIGxldmVscyA9IHRva2VuIHw+IHVuaXF1ZSgpIHw+IHJldigpKQ0KICApDQoNCg0KcGxvdF9iYXJfY29kb25zX25hdHVyYWwgPC0gZnVuY3Rpb24oLnNwZWNpZXMgPSBOQSwNCiAgICAgICAgICAgICAgICAgICAgICAgICAgICAgICAgICAgIC5zYXZlID0gRkFMU0UpIHsNCiAgcGxvdF8xIDwtIG5hdHVyYWxfc2VxdWVuY2VfZGF0YSB8Pg0KICAgIGZpbHRlcihzdHJfZGV0ZWN0KG9yZ2FuaXNtLCAuc3BlY2llcykpIHw+DQogICAgbXV0YXRlKGNvZG9ucyA9IHNlcXVlbmNlIHw+DQogICAgICBzdHJfZXh0cmFjdF9hbGwocGF0dGVybiA9ICJbOmFscGhhOl17M30iKSkgfD4NCiAgICBzZWxlY3QoLXNlcXVlbmNlKSB8Pg0KICAgIHVubmVzdChjb2RvbnMpIHw+DQogICAgaW5uZXJfam9pbihjb2Rvbl9hYV90YWJsZSwNCiAgICAgIGJ5ID0gYygiY29kb25zIiA9ICJjb2RvbiIpDQogICAgKSB8Pg0KICAgIGdncGxvdChhZXMoeSA9IHRva2VuLCBmaWxsID0gbW9kZWwpKSArDQogICAgZ2VvbV9iYXIoYWxwaGEgPSAwLjcpICsNCiAgICBzY2FsZV9maWxsX21hbnVhbCh2YWx1ZXMgPSBtb2RlbF9jb2xvcnMpICsNCiAgICBzY2FsZV94X2NvbnRpbnVvdXMoYnJlYWtzID0gYygwLCAxNTAwKSkgKw0KICAgIGZhY2V0X3dyYXAofm1vZGVsLCBucm93ID0gMSkgKw0KICAgIHRoZW1lX2J3KCkgKw0KICAgIHlsYWIoIkNvZG9ucyIpICsNCiAgICB4bGFiKCIiKSArDQogICAgZ2d0aXRsZSgiQ29kb24gZGlzdHJpYnV0aW9uIGFjcm9zcyBzZXF1ZW5jZXMiKSArDQogICAgdGhlbWUoDQogICAgICBwbG90LnRpdGxlID0gZWxlbWVudF90ZXh0KGhqdXN0ID0gMC41KSwNCiAgICAgIGxlZ2VuZC5rZXkuc3BhY2luZy55ID0gdW5pdCgwLjIsICJjbSIpLA0KICAgICAgbGVnZW5kLmtleS53aWR0aCA9IHVuaXQoMSwgImNtIiksDQogICAgICBsZWdlbmQua2V5LmhlaWdodCA9IHVuaXQoMSwgImNtIiksDQogICAgICBzdHJpcC5iYWNrZ3JvdW5kID0gZWxlbWVudF9ibGFuaygpLA0KICAgICAgc3RyaXAudGV4dC54ID0gZWxlbWVudF9ibGFuaygpDQogICAgKSArDQogICAgZ3VpZGVzKGZpbGwgPSBndWlkZV9sZWdlbmQodGl0bGUgPSAiTW9kZWwiKSkNCg0KICBwbG90XzIgPC0gbmF0dXJhbF9zZXF1ZW5jZV9kYXRhIHw+DQogICAgZmlsdGVyKHN0cl9kZXRlY3Qob3JnYW5pc20sIC5zcGVjaWVzKSkgfD4NCiAgICBtdXRhdGUoY29kb25zID0gc2VxdWVuY2UgfD4NCiAgICAgIHN0cl9leHRyYWN0X2FsbChwYXR0ZXJuID0gIls6YWxwaGE6XXszfSIpKSB8Pg0KICAgIHNlbGVjdCgtc2VxdWVuY2UpIHw+DQogICAgdW5uZXN0KGNvZG9ucykgfD4NCiAgICBpbm5lcl9qb2luKGNvZG9uX2FhX3RhYmxlLA0KICAgICAgYnkgPSBjKCJjb2RvbnMiID0gImNvZG9uIikNCiAgICApIHw+DQogICAgZ3JvdXBfYnkoY29kb25zLCBvcmdhbmlzbSwgbW9kZWwpIHw+DQogICAgbmVzdChkYXRhID0gZGVzY3JpcHRpb24pIHw+DQogICAgbXV0YXRlKG5iX29mX3NlcXVlbmNlcyA9IGxlbmd0aCh1bmxpc3QoZGF0YSkgfD4gdW5pcXVlKCkpKSB8Pg0KICAgIGdncGxvdChhZXMoeSA9IHRva2VuLCB4ID0gbmJfb2Zfc2VxdWVuY2VzLCBmaWxsID0gbW9kZWwpKSArDQogICAgZ2VvbV9jb2woYWxwaGEgPSAwLjcpICsNCiAgICBzY2FsZV9maWxsX21hbnVhbCh2YWx1ZXMgPSBtb2RlbF9jb2xvcnMpICsNCiAgICBzY2FsZV94X2NvbnRpbnVvdXMoYnJlYWtzID0gYygwLCA1MCkpICsNCiAgICBmYWNldF93cmFwKH5tb2RlbCwgbnJvdyA9IDEpICsNCiAgICB0aGVtZV9idygpICsNCiAgICB5bGFiKCJDb2RvbnMiKSArDQogICAgeGxhYigiIikgKw0KICAgIGdndGl0bGUoIk51bWJlciBvZiBzZXF1ZW5jZXMgd2hlcmUgdGhlIGNvZG9uIGlzIHByZXNlbnQiKSArDQogICAgdGhlbWUoDQogICAgICBwbG90LnRpdGxlID0gZWxlbWVudF90ZXh0KGhqdXN0ID0gMC41KSwNCiAgICAgIGxlZ2VuZC5rZXkuc3BhY2luZy55ID0gdW5pdCgwLjIsICJjbSIpLA0KICAgICAgbGVnZW5kLmtleS53aWR0aCA9IHVuaXQoMSwgImNtIiksDQogICAgICBsZWdlbmQua2V5LmhlaWdodCA9IHVuaXQoMSwgImNtIiksDQogICAgICBzdHJpcC5iYWNrZ3JvdW5kID0gZWxlbWVudF9ibGFuaygpLA0KICAgICAgc3RyaXAudGV4dC54ID0gZWxlbWVudF9ibGFuaygpDQogICAgKSArDQogICAgZ3VpZGVzKGZpbGwgPSBndWlkZV9sZWdlbmQodGl0bGUgPSAiTW9kZWwiKSkNCg0KICBwbG90IDwtIHBsb3RfMSArIHBsb3RfMiArDQogICAgcGxvdF9sYXlvdXQoDQogICAgICB3aWR0aHMgPSBjKDAuNSwgMC41KSwNCiAgICAgIGd1aWRlcyA9ICJjb2xsZWN0IiwNCiAgICAgIGF4ZXMgPSAiY29sbGVjdCINCiAgICApICsNCiAgICBwbG90X2Fubm90YXRpb24oDQogICAgICB0aXRsZSA9IHBhc3RlMCgiQ29kb24gZGlzdHJpYnV0aW9uIGZvciBuYXR1cmFsIHNlcXVlbmNlcyBhbW9uZyB0b3AgMTAlIENTSSBmb3IgIiwgLnNwZWNpZXMpLA0KICAgICAgdGhlbWUgPSB0aGVtZShwbG90LnRpdGxlID0gZWxlbWVudF90ZXh0KGhqdXN0ID0gMC41KSkgJg0KICAgICAgICB0aGVtZShwbG90LnRpdGxlID0gZWxlbWVudF90ZXh0KHNpemUgPSAxOCkpDQogICAgKQ0KDQogIGlmICguc2F2ZSA9PSBUUlVFKSB7DQogICAgZ2dzYXZlKHBsb3QsDQogICAgICBmaWxlbmFtZSA9IHBhc3RlMCgNCiAgICAgICAgImNvZG9uX2Rpc3RyaWJ1dGlvbl9uYXR1cmFsX3NlcXVlbmNlc18iLA0KICAgICAgICAuc3BlY2llcywNCiAgICAgICAgIi5zdmciDQogICAgICApLA0KICAgICAgaGVpZ2h0ID0gMTAsDQogICAgICB3aWR0aCA9IDE0DQogICAgKQ0KICB9DQogIHBsb3QNCn0NCg0KcGxvdF9iYXJfY29kb25zX2JlbmNobWFyayA8LSBmdW5jdGlvbiguc3BlY2llcyA9IE5BLA0KICAgICAgICAgICAgICAgICAgICAgICAgICAgICAgICAgICAgICAuc2F2ZSA9IEZBTFNFKSB7DQogIHBsb3RfMSA8LSBiZW5jaG1hcmtfc2VxdWVuY2VfZGF0YSB8Pg0KICAgIGZpbHRlcihzdHJfZGV0ZWN0KG9yZ2FuaXNtLCAuc3BlY2llcykpIHw+DQogICAgbXV0YXRlKGNvZG9ucyA9IHNlcXVlbmNlIHw+DQogICAgICBzdHJfZXh0cmFjdF9hbGwocGF0dGVybiA9ICJbOmFscGhhOl17M30iKSkgfD4NCiAgICBzZWxlY3QoLXNlcXVlbmNlKSB8Pg0KICAgIHVubmVzdChjb2RvbnMpIHw+DQogICAgaW5uZXJfam9pbihjb2Rvbl9hYV90YWJsZSwNCiAgICAgIGJ5ID0gYygiY29kb25zIiA9ICJjb2RvbiIpDQogICAgKSB8Pg0KICAgIGdncGxvdChhZXMoeSA9IHRva2VuLCBmaWxsID0gbW9kZWwpKSArDQogICAgZ2VvbV9iYXIoYWxwaGEgPSAwLjcpICsNCiAgICBzY2FsZV9maWxsX21hbnVhbCh2YWx1ZXMgPSBtb2RlbF9jb2xvcnNbLTFdKSArDQogICAgc2NhbGVfeF9jb250aW51b3VzKGJyZWFrcyA9IGMoMCwgMTUwMCkpICsNCiAgICBmYWNldF93cmFwKH5tb2RlbCwgbnJvdyA9IDEpICsNCiAgICB0aGVtZV9idygpICsNCiAgICB5bGFiKCJDb2RvbnMiKSArDQogICAgeGxhYigiIikgKw0KICAgIGdndGl0bGUoIkNvZG9uIGRpc3RyaWJ1dGlvbiBhY3Jvc3Mgc2VxdWVuY2VzIikgKw0KICAgIHRoZW1lKA0KICAgICAgcGxvdC50aXRsZSA9IGVsZW1lbnRfdGV4dChoanVzdCA9IDAuNSksDQogICAgICBsZWdlbmQua2V5LnNwYWNpbmcueSA9IHVuaXQoMC4yLCAiY20iKSwNCiAgICAgIGxlZ2VuZC5rZXkud2lkdGggPSB1bml0KDEsICJjbSIpLA0KICAgICAgbGVnZW5kLmtleS5oZWlnaHQgPSB1bml0KDEsICJjbSIpLA0KICAgICAgc3RyaXAuYmFja2dyb3VuZCA9IGVsZW1lbnRfYmxhbmsoKSwNCiAgICAgIHN0cmlwLnRleHQueCA9IGVsZW1lbnRfYmxhbmsoKQ0KICAgICkgKw0KICAgIGd1aWRlcyhmaWxsID0gZ3VpZGVfbGVnZW5kKHRpdGxlID0gIk1vZGVsIikpDQoNCiAgcGxvdF8yIDwtIGJlbmNobWFya19zZXF1ZW5jZV9kYXRhIHw+DQogICAgZmlsdGVyKHN0cl9kZXRlY3Qob3JnYW5pc20sIC5zcGVjaWVzKSkgfD4NCiAgICBtdXRhdGUoY29kb25zID0gc2VxdWVuY2UgfD4NCiAgICAgIHN0cl9leHRyYWN0X2FsbChwYXR0ZXJuID0gIls6YWxwaGE6XXszfSIpKSB8Pg0KICAgIHNlbGVjdCgtc2VxdWVuY2UpIHw+DQogICAgdW5uZXN0KGNvZG9ucykgfD4NCiAgICBpbm5lcl9qb2luKGNvZG9uX2FhX3RhYmxlLA0KICAgICAgYnkgPSBjKCJjb2RvbnMiID0gImNvZG9uIikNCiAgICApIHw+DQogICAgZ3JvdXBfYnkoY29kb25zLCBvcmdhbmlzbSwgbW9kZWwpIHw+DQogICAgbmVzdChkYXRhID0gcHJvdGVpbikgfD4NCiAgICBtdXRhdGUobmJfb2Zfc2VxdWVuY2VzID0gbGVuZ3RoKHVubGlzdChkYXRhKSB8PiB1bmlxdWUoKSkpIHw+DQogICAgZ2dwbG90KGFlcyh5ID0gdG9rZW4sIHggPSBuYl9vZl9zZXF1ZW5jZXMsIGZpbGwgPSBtb2RlbCkpICsNCiAgICBnZW9tX2NvbChhbHBoYSA9IDAuNykgKw0KICAgIHNjYWxlX2ZpbGxfbWFudWFsKHZhbHVlcyA9IG1vZGVsX2NvbG9yc1stMV0pICsNCiAgICBzY2FsZV94X2NvbnRpbnVvdXMoYnJlYWtzID0gYygwLCA1MCkpICsNCiAgICBmYWNldF93cmFwKH5tb2RlbCwgbnJvdyA9IDEpICsNCiAgICB0aGVtZV9idygpICsNCiAgICB5bGFiKCJDb2RvbnMiKSArDQogICAgeGxhYigiIikgKw0KICAgIGdndGl0bGUoIk51bWJlciBvZiBzZXF1ZW5jZXMgd2hlcmUgdGhlIGNvZG9uIGlzIHByZXNlbnQiKSArDQogICAgdGhlbWUoDQogICAgICBwbG90LnRpdGxlID0gZWxlbWVudF90ZXh0KGhqdXN0ID0gMC41KSwNCiAgICAgIGxlZ2VuZC5rZXkuc3BhY2luZy55ID0gdW5pdCgwLjIsICJjbSIpLA0KICAgICAgbGVnZW5kLmtleS53aWR0aCA9IHVuaXQoMSwgImNtIiksDQogICAgICBsZWdlbmQua2V5LmhlaWdodCA9IHVuaXQoMSwgImNtIiksDQogICAgICBzdHJpcC5iYWNrZ3JvdW5kID0gZWxlbWVudF9ibGFuaygpLA0KICAgICAgc3RyaXAudGV4dC54ID0gZWxlbWVudF9ibGFuaygpDQogICAgKSArDQogICAgZ3VpZGVzKGZpbGwgPSBndWlkZV9sZWdlbmQodGl0bGUgPSAiTW9kZWwiKSkNCg0KICBwbG90IDwtIHBsb3RfMSArIHBsb3RfMiArDQogICAgcGxvdF9sYXlvdXQoDQogICAgICB3aWR0aHMgPSBjKDAuNSwgMC41KSwNCiAgICAgIGd1aWRlcyA9ICJjb2xsZWN0IiwNCiAgICAgIGF4ZXMgPSAiY29sbGVjdCINCiAgICApICsNCiAgICBwbG90X2Fubm90YXRpb24oDQogICAgICB0aXRsZSA9IHBhc3RlMCgiQ29kb24gZGlzdHJpYnV0aW9uIHdpdGhpbiBiZW5jaG1hcmsgc2VxdWVuY2VzIG9wdGltaXplZCBmb3IgIiwgLnNwZWNpZXMpLA0KICAgICAgdGhlbWUgPSB0aGVtZShwbG90LnRpdGxlID0gZWxlbWVudF90ZXh0KGhqdXN0ID0gMC41KSkgJg0KICAgICAgICB0aGVtZShwbG90LnRpdGxlID0gZWxlbWVudF90ZXh0KHNpemUgPSAxOCkpDQogICAgKQ0KDQoNCiAgaWYgKC5zYXZlID09IFRSVUUpIHsNCiAgICBnZ3NhdmUocGxvdCwNCiAgICAgIGZpbGVuYW1lID0gcGFzdGUwKA0KICAgICAgICAiY29kb25fZGlzdHJpYnV0aW9uX2JlbmNobWFya19zZXF1ZW5jZXNfIiwNCiAgICAgICAgLnNwZWNpZXMsDQogICAgICAgICIuc3ZnIg0KICAgICAgKSwNCiAgICAgIGhlaWdodCA9IDEwLA0KICAgICAgd2lkdGggPSAxNA0KICAgICkNCiAgfQ0KICBwbG90DQp9DQpgYGANCg0KDQoNCg0KIyBuYXR1cmFsIHNlcXVlbmNlcw0KDQpgYGB7cn0NCm5hdHVyYWxfc2VxdWVuY2VfZGF0YSA8LSByZWFkX3hsc3goIlN1cHBsZW1lbnRhcnkgRGF0YSAxLnhsc3giLCBzaGVldCA9ICJETkEgc2VxdWVuY2VzIikgfD4NCiAgbXV0YXRlKG9yZ2FuaXNtID0gb3JnYW5pc20gfD4gc3RyX3JlbW92ZSgiIHN0ci4gSy0xMiBzdWJzdHIuIE1HMTY1NSIpKSB8Pg0KICBwaXZvdF9sb25nZXIoDQogICAgY29scyA9IG1hdGNoZXMoIkROQSIpLA0KICAgIG5hbWVzX3RvID0gIm1vZGVsIiwNCiAgICB2YWx1ZXNfdG8gPSAic2VxdWVuY2UiDQogICkgfD4NCiAgbXV0YXRlKG1vZGVsID0gbW9kZWwgfD4NCiAgICBzdHJfcmVtb3ZlKCJfLiokIikpIHw+DQogICMgSUNPUiBhcmUgTkEgZm9yIG90aGVyIG9yZ2FuaXNtcw0KICBkcm9wX25hKCkgfD4NCiAgbXV0YXRlKG1vZGVsID0gZmFjdG9yKA0KICAgIGlmZWxzZSgNCiAgICAgIHN0cl9kZXRlY3QobW9kZWwsIG1vZGVsX3BhdHRlcm5zWzFdKSwNCiAgICAgIG1vZGVsX2xldmVsc1sxXSwNCiAgICAgIGlmZWxzZSgNCiAgICAgICAgc3RyX2RldGVjdChtb2RlbCwgbW9kZWxfcGF0dGVybnNbMl0pLA0KICAgICAgICBtb2RlbF9sZXZlbHNbMl0sDQogICAgICAgIGlmZWxzZSgNCiAgICAgICAgICBzdHJfZGV0ZWN0KG1vZGVsLCBtb2RlbF9wYXR0ZXJuc1szXSksDQogICAgICAgICAgbW9kZWxfbGV2ZWxzWzNdLA0KICAgICAgICAgIGlmZWxzZSgNCiAgICAgICAgICAgIHN0cl9kZXRlY3QobW9kZWwsIG1vZGVsX3BhdHRlcm5zWzRdKSwNCiAgICAgICAgICAgIG1vZGVsX2xldmVsc1s0XSwNCiAgICAgICAgICAgIGlmZWxzZSgNCiAgICAgICAgICAgICAgc3RyX2RldGVjdChtb2RlbCwgbW9kZWxfcGF0dGVybnNbNV0pLA0KICAgICAgICAgICAgICBtb2RlbF9sZXZlbHNbNV0sDQogICAgICAgICAgICAgIGlmZWxzZSgNCiAgICAgICAgICAgICAgICBzdHJfZGV0ZWN0KG1vZGVsLCBtb2RlbF9wYXR0ZXJuc1s2XSksDQogICAgICAgICAgICAgICAgbW9kZWxfbGV2ZWxzWzZdLA0KICAgICAgICAgICAgICAgIGlmZWxzZSgNCiAgICAgICAgICAgICAgICAgIHN0cl9kZXRlY3QobW9kZWwsIG1vZGVsX3BhdHRlcm5zWzddKSwNCiAgICAgICAgICAgICAgICAgIG1vZGVsX2xldmVsc1s3XSwNCiAgICAgICAgICAgICAgICAgIE5BDQogICAgICAgICAgICAgICAgKQ0KICAgICAgICAgICAgICApDQogICAgICAgICAgICApDQogICAgICAgICAgKQ0KICAgICAgICApDQogICAgICApDQogICAgKSwNCiAgICBsZXZlbHMgPSBtb2RlbF9sZXZlbHMNCiAgKSkgfD4NCiAgc2VsZWN0KG9yZ2FuaXNtLCBkZXNjcmlwdGlvbiwgbW9kZWwsIHNlcXVlbmNlKQ0KDQpvcmdhbmlzbV9sZXZlbHMgPC0gbmF0dXJhbF9zZXF1ZW5jZV9kYXRhIHw+DQogIHB1bGwob3JnYW5pc20pIHw+DQogIHVuaXF1ZSgpDQpgYGANCg0KYGBge3J9DQpuYXR1cmFsX3NlcXVlbmNlX2RhdGEgfD4NCiAgZ3JvdXBfYnkob3JnYW5pc20sIG1vZGVsKSB8Pg0KICBzdW1tYXJpc2UoDQogICAgbmJfb2Zfc2VxdWVuY2VzID0gbigpLA0KICAgIC5ncm91cHMgPSAiZHJvcCINCiAgKQ0KYGBgDQoNCiMjIGNvZG9uIGRpc3RyaWJ1dGlvbg0KDQpgYGB7cn0NCm5hdHVyYWxfc2VxdWVuY2VfZGF0YSB8PiANCiAgbXV0YXRlKGNvZG9uPXNlcXVlbmNlIHw+IHN0cl9leHRyYWN0X2FsbCgiW0EtWl17M30iKSkgfD4gDQogIHVubmVzdF9sb25nZXIoY29kb24pIHw+IA0KICBpbm5lcl9qb2luKGNvZG9uX2FhX3RhYmxlLCBieT1qb2luX2J5KGNvZG9uPT1jb2RvbikpIHw+IA0KICBtdXRhdGUobW9kZWw9bW9kZWwgfD4gc3RyX3JlbW92ZV9hbGwoIlxuIikpIHw+IA0KICBncm91cF9ieShvcmdhbmlzbSwgbW9kZWwsIHRva2VuKSB8PiANCiAgc3VtbWFyaXNlKGNvZG9uX2NvdW50PW4oKSkgfD4gDQogIHdyaXRlX3RzdigibmF0dXJhbF9zZXF1ZW5jZXNfY29kb25fY291bnRzLnRzdiIpDQpgYGANCg0KYGBge3IgZmlnLmhlaWdodD0xMCwgZmlnLndpZHRoPTE0fQ0KcGxvdF9iYXJfY29kb25zX25hdHVyYWwoDQogIC5zcGVjaWVzID0gIkVzY2hlcmljaGlhIGNvbGkiLA0KICAuc2F2ZSA9IEZBTFNFDQopDQpgYGANCg0KDQpgYGB7ciBmaWcuaGVpZ2h0PTEwLCBmaWcud2lkdGg9MTR9DQpwbG90X2Jhcl9jb2RvbnNfbmF0dXJhbCgNCiAgLnNwZWNpZXMgPSAiU2FjY2hhcm9teWNlcyBjZXJldmlzaWFlIiwNCiAgLnNhdmUgPSBGQUxTRQ0KKQ0KYGBgDQoNCmBgYHtyIGZpZy5oZWlnaHQ9MTAsIGZpZy53aWR0aD0xNH0NCnBsb3RfYmFyX2NvZG9uc19uYXR1cmFsKA0KICAuc3BlY2llcyA9ICJBcmFiaWRvcHNpcyB0aGFsaWFuYSIsDQogIC5zYXZlID0gRkFMU0UNCikNCmBgYA0KDQpgYGB7ciBmaWcuaGVpZ2h0PTEwLCBmaWcud2lkdGg9MTR9DQpwbG90X2Jhcl9jb2RvbnNfbmF0dXJhbCgNCiAgLnNwZWNpZXMgPSAiTXVzIG11c2N1bHVzIiwNCiAgLnNhdmUgPSBGQUxTRQ0KKQ0KYGBgDQpgYGB7ciBmaWcuaGVpZ2h0PTEwLCBmaWcud2lkdGg9MTR9DQpwbG90X2Jhcl9jb2RvbnNfbmF0dXJhbCgNCiAgLnNwZWNpZXMgPSAiSG9tbyBzYXBpZW5zIiwNCiAgLnNhdmUgPSBGQUxTRQ0KKQ0KYGBgDQoNCiMjICVtaW5tYXggDQoNCmBgYHtyfQ0KbmF0dXJhbF9taW5tYXhfcmVzdWx0cyA8LSBuYXR1cmFsX3NlcXVlbmNlX2RhdGEgfD4NCiAgc2VsZWN0KG9yZ2FuaXNtLCBkZXNjcmlwdGlvbiwgbW9kZWwsIHNlcXVlbmNlKSB8Pg0KICBtdXRhdGUoc3BlY2llcyA9IGlmZWxzZShzdHJfZGV0ZWN0KG9yZ2FuaXNtLCAiY29saSIpLCAiZWNvbGkiLA0KICAgIGlmZWxzZShzdHJfZGV0ZWN0KG9yZ2FuaXNtLCAiY2VyZXZpc2lhZSIpLCAic2FjY2hhcm9teWNlcyIsDQogICAgICBpZmVsc2Uoc3RyX2RldGVjdChvcmdhbmlzbSwgInRoYWxpYW5hIiksICJhcmFiaWRvcHNpcyIsDQogICAgICAgIGlmZWxzZShzdHJfZGV0ZWN0KG9yZ2FuaXNtLCAibXVzY3VsdXMiKSwgIm1vdXNlIiwNCiAgICAgICAgICBpZmVsc2Uoc3RyX2RldGVjdChvcmdhbmlzbSwgInNhcGllbnMiKSwgImh1bWFuIiwgTkEpDQogICAgICAgICkNCiAgICAgICkNCiAgICApDQogICkpIHw+DQogIG5lc3QoZGF0YSA9IGMoc2VxdWVuY2UsIHNwZWNpZXMpKSB8Pg0KICBtdXRhdGUobWlubWF4ID0gbWFwKGRhdGEsIGNvbXB1dGVfbWlubWF4KSkgfD4NCiAgdW5uZXN0KGRhdGEpIHw+DQogIHVubmVzdChtaW5tYXgpIHw+DQogIGdyb3VwX2J5KG9yZ2FuaXNtLCBtb2RlbCwgZGVzY3JpcHRpb24pIHw+DQogIG11dGF0ZShpbmRleCA9IHJvd19udW1iZXIoKSkNCmBgYA0KDQojIyBzZWxlY3RlZCBnZW5lcw0KDQpFYzogTkNfMDAwOTEzLjM6MzM4MzI1LTMzOTc0MyB5YWhHIA0KDQpTYzogTk1fMDAxMTc5NDI0LjE6MS0xNDEwIFNFUjMzIA0KDQpBdDogTk1fMDAxMzQwNzg2LjE6Mjc3LTE1ODcgQVQ0RzEyNTQwIA0KDQpNbTogTk1fMDAxNDE2NDUzLjE6MzI0LTE4MDUgQ3NhZCANCg0KSHM6IFhNXzA1NDMxODI3Ni4xOjMzNy0yMjIzIFpCVEI3QyANCg0KYGBge3IgZmlnLmhlaWdodD04LCBmaWcud2lkdGg9MTB9DQpwbG90IDwtIG5hdHVyYWxfbWlubWF4X3Jlc3VsdHMgfD4NCiAgZmlsdGVyKHN0cl9kZXRlY3QoDQogICAgZGVzY3JpcHRpb24sDQogICAgIk5DXzAwMDkxMy4zOjMzODMyNS0zMzk3NDMgeWFoR3xOTV8wMDExNzk0MjQuMToxLTE0MTAgU0VSMzN8Tk1fMDAxMzQwNzg2LjE6Mjc3LTE1ODcgQVQ0RzEyNTQwfE5NXzAwMTQxNjQ1My4xOjMyNC0xODA1IENzYWR8WE1fMDU0MzE4Mjc2LjE6MzM3LTIyMjMgWkJUQjdDIg0KICApKSB8Pg0KICBtdXRhdGUoZGVzY3JpcHRpb24gPSBkZXNjcmlwdGlvbiB8Pg0KICAgIHN0cl9leHRyYWN0KCIgLiokIikgfD4NCiAgICBzdHJfcmVtb3ZlKCIgIikpIHw+DQogIG11dGF0ZShkZXNjcmlwdGlvbiA9IGZhY3RvcihkZXNjcmlwdGlvbiwNCiAgICBsZXZlbHMgPSBjKA0KICAgICAgInlhaEciLA0KICAgICAgIlNFUjMzIiwNCiAgICAgICJBVDRHMTI1NDAiLA0KICAgICAgIkNzYWQiLA0KICAgICAgIlpCVEI3QyINCiAgICApDQogICkpIHw+DQogIG11dGF0ZSh2YWx1ZSA9IG1pbm1heCAqIDEwMCkgfD4NCiAgZ2dwbG90KGFlcygNCiAgICB5ID0gdmFsdWUsDQogICAgeCA9IGluZGV4LA0KICAgIGNvbG9yID0gbW9kZWwNCiAgKSkgKw0KICBnZW9tX2xpbmUobGluZXdpZHRoID0gMC41KSArDQogIHNjYWxlX2NvbG9yX21hbnVhbCh2YWx1ZXMgPSBtb2RlbF9jb2xvcnMpICsNCiAgZmFjZXRfZ3JpZChtb2RlbCB+IGRlc2NyaXB0aW9uLA0KICAgIHNjYWxlcyA9ICJmcmVlX3giDQogICkgKw0KICBsYWJzKHggPSAiIiwgeSA9ICIiKSArDQogIHRoZW1lX2J3KCkgKw0KICB0aGVtZSgNCiAgICBsZWdlbmQucG9zaXRpb24gPSAibm9uZSIsDQogICAgdGhlbWUoDQogICAgICBsZWdlbmQucG9zaXRpb24gPSAibm9uZSIsDQogICAgICBzdHJpcC5iYWNrZ3JvdW5kID0gZWxlbWVudF9ibGFuaygpLA0KICAgICAgcGFuZWwuYm9yZGVyID0gZWxlbWVudF9ibGFuaygpDQogICAgKQ0KICApICsNCiAgdGhlbWUoc3RyaXAuYmFja2dyb3VuZCA9IGVsZW1lbnRfYmxhbmsoKSkNCnBsb3QNCmdnc2F2ZSgNCiAgcGxvdCA9IHBsb3QsDQogIGZpbGVuYW1lID0gInNlbGVjdGVkX21pbm1heC5zdmciLA0KICBoZWlnaHQgPSA4LA0KICB3aWR0aCA9IDEwDQopDQpgYGANCg0KIyMgcGxvdCBzcGVjaWVzDQoNCmBgYHtyfQ0KcGxvdCA8LSBwbG90X2xpbmVfbWlubWF4KA0KICAuZGF0YV9zdHJ1Y3R1cmUgPSBuYXR1cmFsX21pbm1heF9yZXN1bHRzLA0KICAub3JnYW5pc20gPSBvcmdhbmlzbV9sZXZlbHNbMV0sDQogIC5saW1pdHMgPSBjKC0xLCAxLjIpLA0KICAubmNvbCA9IDcNCikNCg0KIyBnZ3NhdmUocGxvdCwNCiMgICBmaWxlbmFtZSA9ICJtaW5tYXhfbmF0dXJhbF9FY29saV93PTE4LnN2ZyIsDQojICAgaGVpZ2h0ID0gMTIwLA0KIyAgIHdpZHRoID0gMjAsDQojICAgbGltaXRzaXplID0gRkFMU0UNCiMgKQ0KYGBgDQoNCmBgYHtyfQ0KcGxvdCA8LSBwbG90X2xpbmVfbWlubWF4KA0KICAuZGF0YV9zdHJ1Y3R1cmUgPSBuYXR1cmFsX21pbm1heF9yZXN1bHRzLA0KICAub3JnYW5pc20gPSBvcmdhbmlzbV9sZXZlbHNbMl0sDQogIC5saW1pdHMgPSBjKC0xLCAxLjIpLA0KICAubmNvbCA9IDYNCikNCg0KIyBnZ3NhdmUocGxvdCwNCiMgICBmaWxlbmFtZSA9ICJtaW5tYXhfbmF0dXJhbF9TY2VyZXZpc2lhZV93PTE4LnN2ZyIsDQojICAgaGVpZ2h0ID0gMTIwLA0KIyAgIHdpZHRoID0gMTYsDQojICAgbGltaXRzaXplID0gRkFMU0UNCiMgKQ0KYGBgDQoNCmBgYHtyfQ0KcGxvdCA8LSBwbG90X2xpbmVfbWlubWF4KA0KICAuZGF0YV9zdHJ1Y3R1cmUgPSBuYXR1cmFsX21pbm1heF9yZXN1bHRzLA0KICAub3JnYW5pc20gPSBvcmdhbmlzbV9sZXZlbHNbM10sDQogIC5saW1pdHMgPSBjKC0xLCAxLjIpLA0KICAubmNvbCA9IDYNCikNCg0KIyBnZ3NhdmUocGxvdCwNCiMgICBmaWxlbmFtZSA9ICJtaW5tYXhfbmF0dXJhbF9BdGhhbGlhbmFfdz0xOC5zdmciLA0KIyAgIGhlaWdodCA9IDEyMCwNCiMgICB3aWR0aCA9IDE2LA0KIyAgIGxpbWl0c2l6ZSA9IEZBTFNFDQojICkNCmBgYA0KDQpgYGB7cn0NCnBsb3QgPC0gcGxvdF9saW5lX21pbm1heCgNCiAgLmRhdGFfc3RydWN0dXJlID0gbmF0dXJhbF9taW5tYXhfcmVzdWx0cywNCiAgLm9yZ2FuaXNtID0gb3JnYW5pc21fbGV2ZWxzWzRdLA0KICAubGltaXRzID0gYygtMSwgMS4yKSwNCiAgLm5jb2wgPSA2DQopDQoNCiMgZ2dzYXZlKHBsb3QsDQojICAgZmlsZW5hbWUgPSAibWlubWF4X25hdHVyYWxfTW11c2N1bHVzX3c9MTguc3ZnIiwNCiMgICBoZWlnaHQgPSAxMjAsDQojICAgd2lkdGggPSAxNiwNCiMgICBsaW1pdHNpemUgPSBGQUxTRQ0KIyApDQpgYGANCg0KYGBge3J9DQpwbG90IDwtIHBsb3RfbGluZV9taW5tYXgoDQogIC5kYXRhX3N0cnVjdHVyZSA9IG5hdHVyYWxfbWlubWF4X3Jlc3VsdHMsDQogIC5vcmdhbmlzbSA9IG9yZ2FuaXNtX2xldmVsc1s1XSwNCiAgLmxpbWl0cyA9IGMoLTEsIDEuMiksDQogIC5uY29sID0gNg0KKQ0KDQojIGdnc2F2ZShwbG90LA0KIyAgIGZpbGVuYW1lID0gIm1pbm1heF9uYXR1cmFsX0hzYXBpZW5zX3c9MTguc3ZnIiwNCiMgICBoZWlnaHQgPSAxMjAsDQojICAgd2lkdGggPSAxNiwNCiMgICBsaW1pdHNpemUgPSBGQUxTRQ0KIyApDQpgYGANCg0KYGBge3IsIGV2YWw9RkFMU0V9DQpuYXR1cmFsX21pbm1heF9yZXN1bHRzIHw+DQogIHNlbGVjdCgtc3BlY2llcywgLXNlcXVlbmNlKSB8Pg0KICBtdXRhdGUoDQogICAgbW9kZWwgPSBtb2RlbCB8PiBzdHJfcmVtb3ZlX2FsbCgiXG4iKSwNCiAgICBtaW5tYXggPSBtaW5tYXggfD4gcm91bmQoZGlnaXRzID0gMikNCiAgKSB8Pg0KICBwaXZvdF93aWRlcihuYW1lc19mcm9tID0gaW5kZXgsIHZhbHVlc19mcm9tID0gbWlubWF4KSB8Pg0KICB1bml0ZShjb2wgPSAibWlubWF4IiwgbWF0Y2hlcygiXFxkIiksIHNlcCA9ICIsIikgfD4NCiAgbXV0YXRlKG1pbm1heCA9IG1pbm1heCB8PiBzdHJfcmVtb3ZlX2FsbCgiLE5BIikpIHw+DQogIHdyaXRlX3RzdigibmF0dXJhbF9taW5tYXhfcHJvZmlsZXMudHN2IikNCmBgYA0KDQojIyBEVFcNCg0KYGBge3IsIGV2YWw9RkFMU0V9DQpuYXR1cmFsX0RUV19yZXN1bHRzIDwtIG5hdHVyYWxfbWlubWF4X3Jlc3VsdHMgfD4NCiAgc2VsZWN0KC1zcGVjaWVzLCAtaW5kZXgsIC1zZXF1ZW5jZSkgfD4NCiAgZ3JvdXBfYnkob3JnYW5pc20sIGRlc2NyaXB0aW9uLCBtb2RlbCkgfD4NCiAgIyBtaW5tYXggcmVzdWx0cyBhcmUgaW4gbG9uZyBmb3JtYXQNCiAgbmVzdChtaW5tYXggPSBtaW5tYXgpIHw+DQogICMgZHVwbGljYXRlIG1vZGVscw0KICBtdXRhdGUoDQogICAgbW9kZWwxID0gbW9kZWwsIG1pbm1heDEgPSBtaW5tYXgsDQogICAgbW9kZWwyID0gbW9kZWwsIG1pbm1heDIgPSBtaW5tYXgNCiAgKSB8Pg0KICAjIGxpbmsgbW9kZWwgYW5kIG1pbm1heCBwcm9maWxlcw0KICBuZXN0KA0KICAgIGNvbXBhcmlzb24xID0gYyhtb2RlbDEsIG1pbm1heDEpLA0KICAgIGNvbXBhcmlzb24yID0gYyhtb2RlbDIsIG1pbm1heDIpDQogICkgfD4NCiAgdW5ncm91cCgpIHw+DQogIHNlbGVjdChvcmdhbmlzbSwgZGVzY3JpcHRpb24sIGNvbXBhcmlzb24xLCBjb21wYXJpc29uMikgfD4NCiAgZ3JvdXBfYnkob3JnYW5pc20sIGRlc2NyaXB0aW9uKSB8Pg0KICAjIG1ha2UgYWxsIGNvbXBhcmlzb24gYmV0d2VlbiBjKG1vZGVsLHByb2ZpbGVzKQ0KICBjb21wbGV0ZShjb21wYXJpc29uMSwgY29tcGFyaXNvbjIpIHw+DQogIHVubmVzdChjKGNvbXBhcmlzb24xLCBjb21wYXJpc29uMikpIHw+DQogIG5lc3QoZHR3X2RhdGEgPSBjKG1pbm1heDEsIG1pbm1heDIpKSB8Pg0KICAjIGNvbXB1dGUgRFRXDQogIG11dGF0ZShkdHdfb2JqZWN0ID0gbWFwKGR0d19kYXRhLCBjb21wdXRlX0RUVykpIHw+DQogIG11dGF0ZSgNCiAgICBkaXN0YW5jZSA9IG1hcChkdHdfb2JqZWN0LCBwbHVjaygiZGlzdGFuY2UiKSksDQogICAgbm9ybWFsaXplZF9kaXN0YW5jZSA9IG1hcChkdHdfb2JqZWN0LCBwbHVjaygibm9ybWFsaXplZERpc3RhbmNlIikpDQogICkgfD4NCiAgbXV0YXRlKA0KICAgIGRpc3RhbmNlID0gcGFyc2VfbnVtYmVyKGRpc3RhbmNlKSwNCiAgICBub3JtYWxpemVkX2Rpc3RhbmNlID0gcGFyc2VfbnVtYmVyKG5vcm1hbGl6ZWRfZGlzdGFuY2UpDQogICkgfD4NCiAgc2VsZWN0KG9yZ2FuaXNtLCBkZXNjcmlwdGlvbiwgbW9kZWwxLCBtb2RlbDIsIGRpc3RhbmNlLCBub3JtYWxpemVkX2Rpc3RhbmNlKQ0KDQpzYXZlUkRTKG5hdHVyYWxfRFRXX3Jlc3VsdHMsICJuYXR1cmFsX0RUV19yZXN1bHRzLnJkcyIpDQpgYGANCg0KYGBge3J9DQpuYXR1cmFsX0RUV19yZXN1bHRzIDwtIHJlYWRSRFMoZmlsZSA9ICJuYXR1cmFsX0RUV19yZXN1bHRzLnJkcyIpIHw+DQogIG11dGF0ZSgNCiAgICBkaXN0YW5jZSA9IGFzLm51bWVyaWMoZGlzdGFuY2UpLA0KICAgIG5vcm1hbGl6ZWRfZGlzdGFuY2UgPSBhcy5udW1lcmljKG5vcm1hbGl6ZWRfZGlzdGFuY2UpDQogICkNCmBgYA0KDQpgYGB7ciBmaWcuaGVpZ2h0PTUsIGZpZy53aWR0aD03LCBldmFsPVRSVUV9DQpkdHdfZ2VuZV9lY29saSA8LSBkdHc6OmR0dygNCiAgbmF0dXJhbF9taW5tYXhfcmVzdWx0cyB8Pg0KICAgIGRwbHlyOjpmaWx0ZXIoDQogICAgICBvcmdhbmlzbSA9PSAiRXNjaGVyaWNoaWEgY29saSIsDQogICAgICBkZXNjcmlwdGlvbiA9PSAiTkNfMDAwOTEzLjM6MzM4MzI1LTMzOTc0MyB5YWhHIg0KICAgICkgfD4NCiAgICBkcGx5cjo6ZmlsdGVyKG1vZGVsID09ICJOYXR1cmFsIikgfD4NCiAgICBkcGx5cjo6cHVsbChtaW5tYXgpICogMTAwLA0KICBuYXR1cmFsX21pbm1heF9yZXN1bHRzIHw+DQogICAgZHBseXI6OmZpbHRlcigNCiAgICAgIG9yZ2FuaXNtID09ICJFc2NoZXJpY2hpYSBjb2xpIiwNCiAgICAgIGRlc2NyaXB0aW9uID09ICJOQ18wMDA5MTMuMzozMzgzMjUtMzM5NzQzIHlhaEciDQogICAgKSB8Pg0KICAgIGRwbHlyOjpmaWx0ZXIobW9kZWwgPT0gbW9kZWxfbGV2ZWxzWzNdKSB8Pg0KICAgIGRwbHlyOjpwdWxsKG1pbm1heCkgKiAxMDAsDQogIGtlZXAgPSBUUlVFDQopDQoNCmR0dzo6ZHR3UGxvdFR3b1dheShkdHdfZ2VuZV9lY29saSwNCiAgeWxhYiA9ICJOYXR1cmFsIHNlcXVlbmNlIiwNCiAgeGxhYiA9ICJNb2RlbCBzZXF1ZW5jZSIsDQogIHRzLnR5cGUgPSAibCIsDQogIGx0eSA9IDEsDQogIGx3ZCA9IDMsDQogIG9mZnNldCA9IDgwLA0KICBtYXRjaC5jb2wgPSAiZ3JleSINCikNCmBgYA0KDQojIyMjIEVjDQoNCmBgYHtyIGZpZy5oZWlnaHQ9NiwgZmlnLndpZHRoPTh9DQpwbG90IDwtIHBsb3RfdGlsZV9zaW1pbGFyaXR5KA0KICAuZGF0YV9zdHJ1YyA9IG5hdHVyYWxfRFRXX3Jlc3VsdHMgfD4NCiAgICBtdXRhdGUodmFsdWUgPSBub3JtYWxpemVkX2Rpc3RhbmNlKSwNCiAgLm9yZ2FuaXNtID0gb3JnYW5pc21fbGV2ZWxzWzFdLA0KICAubWV0cmljID0gIkRUVyBkaXN0YW5jZSINCikgKw0KICBzY2FsZV9maWxsX2dyYWRpZW50KA0KICAgIGxvdyA9ICIjRjdGRUFEIiwNCiAgICBoaWdoID0gIiMwMTcwOEIiDQogICkNCg0KcGxvdA0KIyBnZ3NhdmUocGxvdCwNCiMgICBmaWxlbmFtZSA9ICJEVFdfZWNvbGlfbmF0dXJhbC5zdmciLA0KIyAgIGhlaWdodCA9IHNpbWlsYXJpdHlfcGxvdF9oZWlnaHQsDQojICAgd2lkdGggPSBzaW1pbGFyaXR5X3Bsb3Rfd2lkdGgNCiMgKQ0KYGBgDQoNCiMjIyMgU2MNCg0KYGBge3IgZmlnLmhlaWdodD02LCBmaWcud2lkdGg9OH0NCnBsb3QgPC0gcGxvdF90aWxlX3NpbWlsYXJpdHkoDQogIC5kYXRhX3N0cnVjID0gbmF0dXJhbF9EVFdfcmVzdWx0cyB8Pg0KICAgIG11dGF0ZSh2YWx1ZSA9IG5vcm1hbGl6ZWRfZGlzdGFuY2UpLA0KICAub3JnYW5pc20gPSBvcmdhbmlzbV9sZXZlbHNbMl0sDQogIC5tZXRyaWMgPSAiRFRXIGRpc3RhbmNlIg0KKSArDQogIHNjYWxlX2ZpbGxfZ3JhZGllbnQoDQogICAgbG93ID0gIiNGN0ZFQUQiLA0KICAgIGhpZ2ggPSAiIzAxNzA4QiINCiAgKQ0KDQpwbG90DQojIGdnc2F2ZShwbG90LA0KIyAgIGZpbGVuYW1lID0gIkRUV19zY2VyZXZpc2lhZV9uYXR1cmFsLnN2ZyIsDQojICAgaGVpZ2h0ID0gc2ltaWxhcml0eV9wbG90X2hlaWdodCwNCiMgICB3aWR0aCA9IHNpbWlsYXJpdHlfcGxvdF93aWR0aA0KIyApDQpgYGANCg0KIyMjIyBBdA0KDQpgYGB7ciBmaWcuaGVpZ2h0PTYsIGZpZy53aWR0aD04fQ0KcGxvdCA8LSBwbG90X3RpbGVfc2ltaWxhcml0eSgNCiAgLmRhdGFfc3RydWMgPSBuYXR1cmFsX0RUV19yZXN1bHRzIHw+DQogICAgbXV0YXRlKHZhbHVlID0gbm9ybWFsaXplZF9kaXN0YW5jZSksDQogIC5vcmdhbmlzbSA9IG9yZ2FuaXNtX2xldmVsc1szXSwNCiAgLm1ldHJpYyA9ICJEVFcgZGlzdGFuY2UiDQopICsNCiAgc2NhbGVfZmlsbF9ncmFkaWVudCgNCiAgICBsb3cgPSAiI0Y3RkVBRCIsDQogICAgaGlnaCA9ICIjMDE3MDhCIg0KICApDQoNCnBsb3QNCiMgZ2dzYXZlKHBsb3QsDQojICAgZmlsZW5hbWUgPSAiRFRXX0F0aGFsaWFuYV9uYXR1cmFsLnN2ZyIsDQojICAgaGVpZ2h0ID0gc2ltaWxhcml0eV9wbG90X2hlaWdodCwNCiMgICB3aWR0aCA9IHNpbWlsYXJpdHlfcGxvdF93aWR0aA0KIyApDQpgYGANCg0KIyMjIyBNbQ0KDQpgYGB7ciBmaWcuaGVpZ2h0PTYsIGZpZy53aWR0aD04fQ0KcGxvdCA8LSBwbG90X3RpbGVfc2ltaWxhcml0eSgNCiAgLmRhdGFfc3RydWMgPSBuYXR1cmFsX0RUV19yZXN1bHRzIHw+DQogICAgbXV0YXRlKHZhbHVlID0gbm9ybWFsaXplZF9kaXN0YW5jZSksDQogIC5vcmdhbmlzbSA9IG9yZ2FuaXNtX2xldmVsc1s0XSwNCiAgLm1ldHJpYyA9ICJEVFcgZGlzdGFuY2UiDQopICsNCiAgc2NhbGVfZmlsbF9ncmFkaWVudCgNCiAgICBsb3cgPSAiI0Y3RkVBRCIsDQogICAgaGlnaCA9ICIjMDE3MDhCIg0KICApDQoNCnBsb3QNCiMgZ2dzYXZlKHBsb3QsDQojICAgZmlsZW5hbWUgPSAiRFRXX21tdXNjdWx1c19uYXR1cmFsLnN2ZyIsDQojICAgaGVpZ2h0ID0gc2ltaWxhcml0eV9wbG90X2hlaWdodCwNCiMgICB3aWR0aCA9IHNpbWlsYXJpdHlfcGxvdF93aWR0aA0KIyApDQpgYGANCg0KIyMjIyBIcw0KDQpgYGB7ciBmaWcuaGVpZ2h0PTYsIGZpZy53aWR0aD04fQ0KcGxvdCA8LSBwbG90X3RpbGVfc2ltaWxhcml0eSgNCiAgLmRhdGFfc3RydWMgPSBuYXR1cmFsX0RUV19yZXN1bHRzIHw+DQogICAgbXV0YXRlKHZhbHVlID0gbm9ybWFsaXplZF9kaXN0YW5jZSksDQogIC5vcmdhbmlzbSA9IG9yZ2FuaXNtX2xldmVsc1s1XSwNCiAgLm1ldHJpYyA9ICJEVFcgZGlzdGFuY2UiDQopICsNCiAgc2NhbGVfZmlsbF9ncmFkaWVudCgNCiAgICBsb3cgPSAiI0Y3RkVBRCIsDQogICAgaGlnaCA9ICIjMDE3MDhCIg0KICApDQoNCnBsb3QNCiMgZ2dzYXZlKHBsb3QsDQojICAgZmlsZW5hbWUgPSAiRFRXX2hzYXBpZW5zX25hdHVyYWwuc3ZnIiwNCiMgICBoZWlnaHQgPSBzaW1pbGFyaXR5X3Bsb3RfaGVpZ2h0LA0KIyAgIHdpZHRoID0gc2ltaWxhcml0eV9wbG90X3dpZHRoDQojICkNCmBgYA0KDQojIyMjIGFsbA0KDQpgYGB7ciBmaWcuaGVpZ2h0PTYsIGZpZy53aWR0aD04fQ0KcGxvdCA8LSBwbG90X3RpbGVfc2ltaWxhcml0eSgNCiAgLmRhdGFfc3RydWMgPSBuYXR1cmFsX0RUV19yZXN1bHRzIHw+DQogICAgbXV0YXRlKHZhbHVlID0gbm9ybWFsaXplZF9kaXN0YW5jZSksDQogIC5vcmdhbmlzbSA9ICIuKiIsDQogIC5tZXRyaWMgPSAiRFRXIGRpc3RhbmNlIg0KKSArDQogIHNjYWxlX2ZpbGxfZ3JhZGllbnQoDQogICAgbG93ID0gIiNGN0ZFQUQiLA0KICAgIGhpZ2ggPSAiIzAxNzA4QiINCiAgKQ0KDQpwbG90DQojIGdnc2F2ZShwbG90LA0KIyAgIGZpbGVuYW1lID0gIkRUV19kaXN0YW5jZXNfYmV0d2Vlbl9tb2RlbHNfZm9yX25hdHVyYWxfZ2VuZXMuc3ZnIiwNCiMgICBoZWlnaHQgPSBzaW1pbGFyaXR5X3Bsb3RfaGVpZ2h0LA0KIyAgIHdpZHRoID0gc2ltaWxhcml0eV9wbG90X3dpZHRoDQojICkNCmBgYA0KDQojIyMgbmF0dXJhbCBEVFcgZGlzdGFuY2VzDQoNCmBgYHtyfQ0KZHR3X2JldHdlZW5fbmF0dXJhbF9hbmRfbW9kZWwgPC0gbmF0dXJhbF9EVFdfcmVzdWx0cyB8Pg0KICBmaWx0ZXIobW9kZWwxID09ICJOYXR1cmFsIikgfD4NCiAgZmlsdGVyKG1vZGVsMiAhPSAiTmF0dXJhbCIpIHw+DQogIG11dGF0ZShtb2RlbCA9IG1vZGVsMikgfD4NCiAgbXV0YXRlKG9yZ2FuaXNtID0gZmFjdG9yKG9yZ2FuaXNtLCBsZXZlbHMgPSBvcmdhbmlzbV9sZXZlbHMpKSB8Pg0KICBkcm9wX25hKCkNCmBgYA0KDQoNCmBgYHtyIGZpZy5oZWlnaHQ9MywgZmlnLndpZHRoPTEwfQ0Kc3RhdC50ZXN0X2Rpc3RhbmNlIDwtIGR0d19iZXR3ZWVuX25hdHVyYWxfYW5kX21vZGVsIHw+DQogIGdyb3VwX2J5KG9yZ2FuaXNtKSB8Pg0KICB0X3Rlc3QoZGlzdGFuY2UgfiBtb2RlbCwgcmVmLmdyb3VwID0gbW9kZWxfbGV2ZWxzWzNdKSAgDQpzdGF0LnRlc3RfZGlzdGFuY2UNCmBgYA0KDQoNCmBgYHtyIGZpZy5oZWlnaHQ9MywgZmlnLndpZHRoPTE1fQ0Kc3RhdC50ZXN0X2Rpc3RhbmNlIDwtIHN0YXQudGVzdF9kaXN0YW5jZSB8Pg0KICBhZGRfeV9wb3NpdGlvbigNCiAgICBzY2FsZXMgPSAiZnJlZV95IiwNCiAgICBzdGVwLmluY3JlYXNlID0gMC4yDQogICkgfD4NCiAgbXV0YXRlKHAuYWRqLnNpZ25pZiA9IGlmZWxzZShwLmFkai5zaWduaWYgPT0gIm5zIiwgIiIsIHAuYWRqLnNpZ25pZikpDQoNCnBsb3QgPC0gZHR3X2JldHdlZW5fbmF0dXJhbF9hbmRfbW9kZWwgfD4NCiAgZ2dwbG90KGFlcyh4ID0gbW9kZWwsIHkgPSBkaXN0YW5jZSwgY29sb3IgPSBtb2RlbCkpICsNCiAgZ2VvbV9ib3hwbG90KGNvbG9yID0gImJsYWNrIiwgb3V0bGllci5zaGFwZSA9IE5BKSArDQogIGdlb21faml0dGVyKA0KICAgIGRhdGEgPSB+IHN1YnNldCgueCwgZGVzY3JpcHRpb24gIT0gIk5DXzAwMDkxMy4zOjMzODMyNS0zMzk3NDMgeWFoRyIgJg0KICAgICAgZGVzY3JpcHRpb24gIT0gIk5NXzAwMTE3OTQyNC4xOjEtMTQxMCBTRVIzMyIgJg0KICAgICAgZGVzY3JpcHRpb24gIT0gIk5NXzAwMTM0MDc4Ni4xOjI3Ny0xNTg3IEFUNEcxMjU0MCIgJg0KICAgICAgZGVzY3JpcHRpb24gIT0gIk5NXzAwMTQxNjQ1My4xOjMyNC0xODA1IENzYWQiICYNCiAgICAgIGRlc2NyaXB0aW9uICE9ICJYTV8wNTQzMTgyNzYuMTozMzctMjIyMyBaQlRCN0MiKSwNCiAgICB3aWR0aCA9IDAuMiwgaGVpZ2h0ID0gMCwgc2l6ZSA9IDEsIGFscGhhID0gMC41DQogICkgKw0KICBzY2FsZV9jb2xvcl9tYW51YWwodmFsdWVzID0gbW9kZWxfY29sb3JzW2MoLTEpXSkgKw0KICBnZW9tX2ppdHRlcigNCiAgICBkYXRhID0gfiBzdWJzZXQoLngsIGRlc2NyaXB0aW9uID09ICJOQ18wMDA5MTMuMzozMzgzMjUtMzM5NzQzIHlhaEciIHwNCiAgICAgIGRlc2NyaXB0aW9uID09ICJOTV8wMDExNzk0MjQuMToxLTE0MTAgU0VSMzMiIHwNCiAgICAgIGRlc2NyaXB0aW9uID09ICJOTV8wMDEzNDA3ODYuMToyNzctMTU4NyBBVDRHMTI1NDAiIHwNCiAgICAgIGRlc2NyaXB0aW9uID09ICJOTV8wMDE0MTY0NTMuMTozMjQtMTgwNSBDc2FkIiB8DQogICAgICBkZXNjcmlwdGlvbiA9PSAiWE1fMDU0MzE4Mjc2LjE6MzM3LTIyMjMgWkJUQjdDIiksDQogICAgY29sb3IgPSAiZ3JleTMwIiwgaGVpZ2h0ID0gMCwgc2l6ZSA9IDIsIGFscGhhID0gMC43DQogICkgKw0KICBzdGF0X3B2YWx1ZV9tYW51YWwoDQogICAgc3RhdC50ZXN0X2Rpc3RhbmNlLA0KICAgIGJyYWNrZXQubnVkZ2UueSA9IC0yLCBoaWRlLm5zID0gRkFMU0UsDQogICAgbGFiZWwgPSAie3NjYWxlczo6c2NpZW50aWZpYyhwLmFkail9IHtwLmFkai5zaWduaWZ9IiwNCiAgICB0aXAubGVuZ3RoID0gMA0KICApICsNCiAgc2NhbGVfeV9jb250aW51b3VzKGV4cGFuZCA9IGV4cGFuc2lvbihtdWx0ID0gYygwLCAwLjEpKSkgKw0KICBmYWNldF93cmFwKH5vcmdhbmlzbSwgbmNvbCA9IDUsIHNjYWxlcyA9ICJmcmVlIikgKw0KICBsYWJzKHkgPSAiIiwgeCA9ICIiKSArDQogIHRoZW1lX2J3KCkgKw0KICB0aGVtZSgNCiAgICBheGlzLnRpdGxlLnggPSBlbGVtZW50X2JsYW5rKCksDQogICAgYXhpcy50ZXh0LnggPSBlbGVtZW50X2JsYW5rKCksDQogICAgbGVnZW5kLnRpdGxlID0gZWxlbWVudF9ibGFuaygpLA0KICAgIGxlZ2VuZC5wb3NpdGlvbiA9ICJub25lIiwNCiAgICBzdHJpcC50ZXh0ID0gZWxlbWVudF90ZXh0KGZhY2UgPSAiaXRhbGljIikNCiAgKQ0KcGxvdA0KIyBnZ3NhdmUocGxvdCwNCiMgICBmaWxlbmFtZSA9ICJEVFdfdG9fbmF0dXJhbF9hbGxfc3BlY2llc193aXRoX3N0YXRpc3RpY3Muc3ZnIiwgaGVpZ2h0ID0gMywgd2lkdGggPSAxNQ0KIyApDQpgYGANCg0KYGBge3IsIGV2YWw9RkFMU0V9DQpuYXR1cmFsX0RUV19yZXN1bHRzIHw+DQogIG11dGF0ZSgNCiAgICBtb2RlbDEgPSBtb2RlbDEgfD4gc3RyX3JlbW92ZV9hbGwoIlxuIiksDQogICAgbW9kZWwyID0gbW9kZWwyIHw+IHN0cl9yZW1vdmVfYWxsKCJcbiIpDQogICkgfD4NCiAgc2VsZWN0KG9yZ2FuaXNtLCBkZXNjcmlwdGlvbiwgbW9kZWwxLCBtb2RlbDIsIGRpc3RhbmNlLCBub3JtYWxpemVkX2Rpc3RhbmNlKSB8Pg0KICBtdXRhdGUoDQogICAgZGlzdGFuY2UgPSB1bmxpc3QoZGlzdGFuY2UpLA0KICAgIG5vcm1hbGl6ZWRfZGlzdGFuY2UgPSB1bmxpc3Qobm9ybWFsaXplZF9kaXN0YW5jZSkNCiAgKSB8Pg0KICB3cml0ZV90c3YoIm5hdHVyYWxfRFRXX21pbm1heC50c3YiKQ0KYGBgDQoNCiMjIFJOQSBmb2xkDQoNCmBgYHtyLCBldmFsPUZBTFNFfQ0KbmF0dXJhbF9STkFfZm9sZGluZ19yZXN1bHRzIDwtIG5hdHVyYWxfc2VxdWVuY2VfZGF0YSB8Pg0KICBtdXRhdGUoUk5BX2ZvbGRfb2JqZWN0ID0gbWFwKHNlcXVlbmNlLCBjb21wdXRlX1JOQV9mb2xkKSkgfD4NCiAgc2VsZWN0KG9yZ2FuaXNtLCBkZXNjcmlwdGlvbiwgbW9kZWwsIHNlcXVlbmNlLCBSTkFfZm9sZF9vYmplY3QpDQoNCnNhdmVSRFMobmF0dXJhbF9STkFfZm9sZGluZ19yZXN1bHRzLCAibmF0dXJhbF9STkFfZm9sZGluZy5yZHMiKQ0KYGBgDQoNCmBgYHtyfQ0KbmF0dXJhbF9STkFfZm9sZGluZ19yZXN1bHRzIDwtIHJlYWRSRFMoIm5hdHVyYWxfUk5BX2ZvbGRpbmcucmRzIikgfD4gbXV0YXRlKA0KICBvcmdhbmlzbSA9IGZhY3RvcihvcmdhbmlzbSwgbGV2ZWxzID0gb3JnYW5pc21fbGV2ZWxzKSwNCiAgbWZlID0gbWFwKFJOQV9mb2xkX29iamVjdCwgcGx1Y2soIm1mZSIpKSwNCiAgbWZlID0gYXMubnVtZXJpYyhtZmUpLA0KICBsZW5ndGggPSBzdHJfY291bnQoc2VxdWVuY2UpLA0KICBtZmVfbm9ybWFsaXplZF9ieV9sZW5ndGggPSBtZmUgLyBsZW5ndGgsDQogIEdDX2NvbnRlbnQgPSBzdHJfY291bnQoc2VxdWVuY2UsICJHfEMiKSAvIGxlbmd0aA0KKQ0KYGBgDQoNCiMjIyMgbWZlICMgbGVuZ3RoDQoNCmBgYHtyIGZpZy5oZWlnaHQ9NCwgZmlnLndpZHRoPTZ9DQpwbG90IDwtIG5hdHVyYWxfUk5BX2ZvbGRpbmdfcmVzdWx0cyB8Pg0KICBtdXRhdGUoeV9wbG90ID0gbWZlLCB4X3Bsb3QgPSBsZW5ndGgpIHw+DQogIHBsb3Rfc21vb3RoX21vZGVsKCkgKw0KICBzY2FsZV9jb2xvcl9tYW51YWwodmFsdWVzID0gbW9kZWxfY29sb3JzKSArDQogIHNjYWxlX2ZpbGxfbWFudWFsKHZhbHVlcyA9IG1vZGVsX2NvbG9ycykNCnBsb3QNCg0KIyBnZ3NhdmUocGxvdCwNCiMgICBmaWxlbmFtZSA9ICJSTkFfZm9sZGluZ18xX2xpbmVhcl9yZWxhdGlvbl90b19sZW5ndGhfbmF0dXJhbC5zdmciLA0KIyAgIGhlaWdodCA9IDQsDQojICAgd2lkdGggPSA2DQojICkNCmBgYA0KDQojIyMjIG5vcm1hbGl6ZWQgIyBuYXR1cmFsDQoNCmBgYHtyIGZpZy5oZWlnaHQ9NCwgZmlnLndpZHRoPTZ9DQpwbG90IDwtIGlubmVyX2pvaW4oDQogIG5hdHVyYWxfUk5BX2ZvbGRpbmdfcmVzdWx0cyB8Pg0KICAgIGZpbHRlcihtb2RlbCAhPSAiTmF0dXJhbCIpLA0KICBuYXR1cmFsX1JOQV9mb2xkaW5nX3Jlc3VsdHMgfD4NCiAgICBmaWx0ZXIobW9kZWwgPT0gIk5hdHVyYWwiKSB8Pg0KICAgIG11dGF0ZShyZWZfbm9ybSA9IG1mZV9ub3JtYWxpemVkX2J5X2xlbmd0aCkgfD4NCiAgICBzZWxlY3QoLW1mZV9ub3JtYWxpemVkX2J5X2xlbmd0aCwgLW1vZGVsKSwNCiAgYnkgPSBjKCJvcmdhbmlzbSIsICJkZXNjcmlwdGlvbiIsICJsZW5ndGgiKQ0KKSB8Pg0KICBtdXRhdGUob3JnYW5pc20gPSBmYWN0b3Iob3JnYW5pc20sIGxldmVscyA9IG9yZ2FuaXNtX2xldmVscykpIHw+DQogIG11dGF0ZSh4X3Bsb3QgPSByZWZfbm9ybSwgeV9wbG90ID0gbWZlX25vcm1hbGl6ZWRfYnlfbGVuZ3RoKSB8Pg0KICBwbG90X3Ntb290aF9tb2RlbCgpICsNCiAgc3RhdF9yZWdsaW5lX2VxdWF0aW9uKA0KICAgIGxhYmVsLngubnBjID0gMC42LA0KICAgIGxhYmVsLnkubnBjID0gMC4zNSwNCiAgKSArDQogIHlsYWIoIm1mZSAvIGxlbmd0aCIpICsNCiAgeGxhYigibWZlIC8gbGVuZ3RoIGZvciBuYXR1cmFsIHNlcXVlbmNlIikgKw0KICB4bGltKGMoLTAuNTUsIC0wLjEpKSArDQogIHNjYWxlX2NvbG9yX21hbnVhbCh2YWx1ZXMgPSBtb2RlbF9jb2xvcnNbLTFdKSArDQogIHNjYWxlX2ZpbGxfbWFudWFsKHZhbHVlcyA9IG1vZGVsX2NvbG9yc1stMV0pDQpwbG90DQojIGdnc2F2ZShwbG90LA0KIyAgIGZpbGVuYW1lID0gIlJOQV9mb2xkaW5nXzJfbGluZWFyX2ZpdF9wbG90bm9ybWFsaXplZF9uYXR1cmFsLnN2ZyIsDQojICAgaGVpZ2h0ID0gNCwNCiMgICB3aWR0aCA9IDYNCiMgKQ0KYGBgDQoNCiMjIyMgbm9ybWFsaXplZCBieSBzcGVjaWVzDQoNCmBgYHtyIGZpZy5oZWlnaHQ9NCwgZmlnLndpZHRoPTZ9DQpwbG90IDwtIG5hdHVyYWxfUk5BX2ZvbGRpbmdfcmVzdWx0cyB8Pg0KICBtdXRhdGUodmFsdWUgPSBtZmVfbm9ybWFsaXplZF9ieV9sZW5ndGgpIHw+DQogIHBsb3Rfaml0dGVyX3NwZWNpZXMoKSArDQogIHhsYWIoIm1mZSAvIGxlbmd0aCIpDQpwbG90DQojIGdnc2F2ZShwbG90LA0KIyAgIGZpbGVuYW1lID0gIlJOQV9mb2xkaW5nXzNfc3BlY2llc19zZXBhcmF0aW9uX25vcm1hbGl6ZWRfbmF0dXJhbC5zdmciLA0KIyAgIGhlaWdodCA9IDQsDQojICAgd2lkdGggPSA2DQojICkNCmBgYA0KDQojIyMjIEdDIGJ5IHNwZWNpZXMNCg0KYGBge3IgZmlnLmhlaWdodD00LCBmaWcud2lkdGg9Nn0NCnBsb3QgPC0gbmF0dXJhbF9STkFfZm9sZGluZ19yZXN1bHRzIHw+DQogIG11dGF0ZSh2YWx1ZSA9IEdDX2NvbnRlbnQpIHw+DQogIHBsb3Rfaml0dGVyX3NwZWNpZXMoKSArDQogIHhsYWIoIkdDIGNvbnRlbnQiKQ0KcGxvdA0KIyBnZ3NhdmUocGxvdCwNCiMgICBmaWxlbmFtZSA9ICJSTkFfZm9sZGluZ180X3NwZWNpZXNfc2VwYXJhdGlvbl9HQ19jb250ZW50LnN2ZyIsDQojICAgaGVpZ2h0ID0gNCwNCiMgICB3aWR0aCA9IDYNCiMgKQ0KYGBgDQoNCiMjIyMgR0MgIyBuYXR1cmFsDQoNCmBgYHtyIGZpZy5oZWlnaHQ9NCwgZmlnLndpZHRoPTZ9DQpwbG90IDwtIGlubmVyX2pvaW4oDQogIG5hdHVyYWxfUk5BX2ZvbGRpbmdfcmVzdWx0cyB8Pg0KICAgIGZpbHRlcihtb2RlbCAhPSAiTmF0dXJhbCIpLA0KICBuYXR1cmFsX1JOQV9mb2xkaW5nX3Jlc3VsdHMgfD4NCiAgICBmaWx0ZXIobW9kZWwgPT0gIk5hdHVyYWwiKSB8Pg0KICAgIG11dGF0ZShyZWZfR0MgPSBHQ19jb250ZW50KSB8Pg0KICAgIHNlbGVjdCgtR0NfY29udGVudCwgLW1vZGVsKSwNCiAgYnkgPSBjKCJvcmdhbmlzbSIsICJkZXNjcmlwdGlvbiIsICJsZW5ndGgiKQ0KKSB8Pg0KICBtdXRhdGUob3JnYW5pc20gPSBmYWN0b3Iob3JnYW5pc20sIGxldmVscyA9IG9yZ2FuaXNtX2xldmVscykpIHw+DQogIG11dGF0ZSh4X3Bsb3QgPSByZWZfR0MsIHlfcGxvdCA9IEdDX2NvbnRlbnQpIHw+DQogIHBsb3Rfc21vb3RoX21vZGVsKCkgKw0KICBzdGF0X3JlZ2xpbmVfZXF1YXRpb24oDQogICAgbGFiZWwueC5ucGMgPSAwLjYsDQogICAgbGFiZWwueS5ucGMgPSAwLjM1LA0KICApICsNCiAgeWxhYigiR0MgY29udGVudCIpICsNCiAgeGxhYigiR0MgY29udGVudCBvZiBuYXR1cmFsIHNlcXVlbmNlIikgKw0KICBzY2FsZV9jb2xvcl9tYW51YWwodmFsdWVzID0gbW9kZWxfY29sb3JzWy0xXSkgKw0KICBzY2FsZV9maWxsX21hbnVhbCh2YWx1ZXMgPSBtb2RlbF9jb2xvcnNbLTFdKQ0KDQpwbG90DQojIGdnc2F2ZShwbG90LA0KIyAgIGZpbGVuYW1lID0gIlJOQV9mb2xkaW5nXzVfbGluZWFyX2ZpdF9HQ2NvbnRlbnRfbmF0dXJhbC5zdmciLA0KIyAgIGhlaWdodCA9IDQsDQojICAgd2lkdGggPSA2DQojICkNCmBgYA0KDQpgYGB7ciwgZXZhbD1GQUxTRX0NCm5hdHVyYWxfUk5BX2ZvbGRpbmdfcmVzdWx0cyB8Pg0KICBtdXRhdGUobW9kZWwgPSBtb2RlbCB8PiBzdHJfcmVtb3ZlX2FsbCgiXG4iKSkgfD4NCiAgc2VsZWN0KG9yZ2FuaXNtLCBkZXNjcmlwdGlvbiwgbWZlLCBsZW5ndGgsIG1mZV9ub3JtYWxpemVkX2J5X2xlbmd0aCwgR0NfY29udGVudCkgfD4NCiAgd3JpdGVfdHN2KCJuYXR1cmFsX1JOQV9mb2xkLnRzdiIpDQpgYGANCg0KDQojIGJlbmNobWFyayBzZXF1ZW5jZXMNCg0KYGBge3J9DQojIGltcG9ydCBzZXF1ZW5jZXMgZ2VuZXJhdGVkIGJ5IGRpZmZlcmVudCB0b29scw0KYmVuY2htYXJrX3NlcXVlbmNlX2RhdGEgPC0gcmVhZF94bHN4KCJTdXBwbGVtZW50YXJ5IERhdGEgMi54bHN4Iiwgc2hlZXQgPSAiRE5BIHNlcXVlbmNlcywgQ1NJLCBDSVMtZWxlbWVudCIpIHw+DQogIHNlbGVjdCgtbWF0Y2hlcygiQ1NJIikpIHw+DQogICMgbWFrZSBjb2x1bW5zIGNvbnRhaW5pbmcgZG5hIHNlcXVlbmNlIGFzIHJvd3MNCiAgcGl2b3RfbG9uZ2VyKA0KICAgIGNvbHMgPSBtYXRjaGVzKCJETkEiKSwNCiAgICBuYW1lc190byA9ICJtb2RlbCIsDQogICAgdmFsdWVzX3RvID0gInNlcXVlbmNlIg0KICApIHw+DQogICMgbWFrZSBjb2x1bW5zIGNvbnRhaW5pbmcgQ1NJIGFzIHJvd3MNCiAgcGl2b3RfbG9uZ2VyKA0KICAgIGNvbHMgPSBtYXRjaGVzKCJDSVMiKSwNCiAgICBuYW1lc190byA9ICJtb2RlbF9iaXMiLA0KICAgIHZhbHVlc190byA9ICJuYl9jaXNfZWxlbWVudCINCiAgKSB8Pg0KICBtdXRhdGUoDQogICAgbW9kZWwgPSBtb2RlbCB8PiBzdHJfcmVtb3ZlKCJfZG5hJHxfRE5BJCIpLA0KICAgIG1vZGVsX2JpcyA9IG1vZGVsX2JpcyB8PiBzdHJfcmVtb3ZlKCJfQ0lTJCIpDQogICkgfD4NCiAgZmlsdGVyKG1vZGVsID09IG1vZGVsX2JpcykgfD4NCiAgIyBJQ09SIGFyZSBOQSBmb3Igb3RoZXIgb3JnYW5pc21zDQogIGRyb3BfbmEoKSB8Pg0KICBtdXRhdGUobW9kZWwgPSBmYWN0b3IoDQogICAgaWZlbHNlKA0KICAgICAgc3RyX2RldGVjdChtb2RlbCwgbW9kZWxfcGF0dGVybnNbMV0pLA0KICAgICAgbW9kZWxfbGV2ZWxzWzFdLA0KICAgICAgaWZlbHNlKA0KICAgICAgICBzdHJfZGV0ZWN0KG1vZGVsLCBtb2RlbF9wYXR0ZXJuc1syXSksDQogICAgICAgIG1vZGVsX2xldmVsc1syXSwNCiAgICAgICAgaWZlbHNlKA0KICAgICAgICAgIHN0cl9kZXRlY3QobW9kZWwsIG1vZGVsX3BhdHRlcm5zWzNdKSwNCiAgICAgICAgICBtb2RlbF9sZXZlbHNbM10sDQogICAgICAgICAgaWZlbHNlKA0KICAgICAgICAgICAgc3RyX2RldGVjdChtb2RlbCwgbW9kZWxfcGF0dGVybnNbNF0pLA0KICAgICAgICAgICAgbW9kZWxfbGV2ZWxzWzRdLA0KICAgICAgICAgICAgaWZlbHNlKA0KICAgICAgICAgICAgICBzdHJfZGV0ZWN0KG1vZGVsLCBtb2RlbF9wYXR0ZXJuc1s1XSksDQogICAgICAgICAgICAgIG1vZGVsX2xldmVsc1s1XSwNCiAgICAgICAgICAgICAgaWZlbHNlKA0KICAgICAgICAgICAgICAgIHN0cl9kZXRlY3QobW9kZWwsIG1vZGVsX3BhdHRlcm5zWzZdKSwNCiAgICAgICAgICAgICAgICBtb2RlbF9sZXZlbHNbNl0sDQogICAgICAgICAgICAgICAgaWZlbHNlKA0KICAgICAgICAgICAgICAgICAgc3RyX2RldGVjdChtb2RlbCwgbW9kZWxfcGF0dGVybnNbN10pLA0KICAgICAgICAgICAgICAgICAgbW9kZWxfbGV2ZWxzWzddLA0KICAgICAgICAgICAgICAgICAgTkENCiAgICAgICAgICAgICAgICApDQogICAgICAgICAgICAgICkNCiAgICAgICAgICAgICkNCiAgICAgICAgICApDQogICAgICAgICkNCiAgICAgICkNCiAgICApLA0KICAgIGxldmVscyA9IG1vZGVsX2xldmVscw0KICApKSB8Pg0KICBzZWxlY3Qob3JnYW5pc20sIHByb3RlaW4sIG1vZGVsLCBzZXF1ZW5jZSwgbmJfY2lzX2VsZW1lbnQpDQoNCm9yZ2FuaXNtX2xldmVscyA8LSBiZW5jaG1hcmtfc2VxdWVuY2VfZGF0YSB8Pg0KICBwdWxsKG9yZ2FuaXNtKSB8Pg0KICB1bmlxdWUoKQ0KYGBgDQoNCmBgYHtyfQ0KYmVuY2htYXJrX3NlcXVlbmNlX2RhdGEgfD4NCiAgZ3JvdXBfYnkob3JnYW5pc20sIG1vZGVsKSB8Pg0KICBzdW1tYXJpc2UobmJfb2Zfc2VxdWVuY2VzID0gbigpLCAuZ3JvdXBzID0gImRyb3AiKQ0KYGBgDQoNCiMjIENvZG9uIGRpc3RyaWJ1dGlvbg0KDQpgYGB7cn0NCmJlbmNobWFya19zZXF1ZW5jZV9kYXRhIHw+IA0KICBtdXRhdGUoY29kb249c2VxdWVuY2UgfD4gc3RyX2V4dHJhY3RfYWxsKCJbQS1aXXszfSIpKSB8PiANCiAgdW5uZXN0X2xvbmdlcihjb2RvbikgfD4gDQogIGlubmVyX2pvaW4oY29kb25fYWFfdGFibGUsIGJ5PWpvaW5fYnkoY29kb249PWNvZG9uKSkgfD4gDQogIG11dGF0ZShtb2RlbD1tb2RlbCB8PiBzdHJfcmVtb3ZlX2FsbCgiXG4iKSkgfD4gDQogIGdyb3VwX2J5KG9yZ2FuaXNtLCBtb2RlbCwgdG9rZW4pIHw+IA0KICBzdW1tYXJpc2UoY29kb25fY291bnQ9bigpKSB8PiANCiAgd3JpdGVfdHN2KCJiZW5jaG1hcmtfc2VxdWVuY2VzX2NvZG9uX2NvdW50cy50c3YiKQ0KYGBgDQoNCmBgYHtyIGZpZy5oZWlnaHQ9MTAsIGZpZy53aWR0aD0xNH0NCnBsb3RfYmFyX2NvZG9uc19iZW5jaG1hcmsoDQogIC5zcGVjaWVzID0gIkVzY2hlcmljaGlhIGNvbGkiLA0KICAuc2F2ZSA9IEZBTFNFDQopDQpgYGANCg0KYGBge3IgZmlnLmhlaWdodD0xMCwgZmlnLndpZHRoPTE0fQ0KcGxvdF9iYXJfY29kb25zX2JlbmNobWFyaygNCiAgLnNwZWNpZXMgPSAiU2FjY2hhcm9teWNlcyBjZXJldmlzaWFlIiwNCiAgLnNhdmUgPSBGQUxTRQ0KKQ0KYGBgDQoNCmBgYHtyIGZpZy5oZWlnaHQ9MTAsIGZpZy53aWR0aD0xNH0NCnBsb3RfYmFyX2NvZG9uc19iZW5jaG1hcmsoDQogIC5zcGVjaWVzID0gIkFyYWJpZG9wc2lzIHRoYWxpYW5hIiwNCiAgLnNhdmUgPSBGQUxTRQ0KKQ0KYGBgDQoNCmBgYHtyIGZpZy5oZWlnaHQ9MTAsIGZpZy53aWR0aD0xNH0NCnBsb3RfYmFyX2NvZG9uc19iZW5jaG1hcmsoDQogIC5zcGVjaWVzID0gIk11cyBtdXNjdWx1cyIsDQogIC5zYXZlID0gRkFMU0UNCikNCmBgYA0KDQpgYGB7ciBmaWcuaGVpZ2h0PTEwLCBmaWcud2lkdGg9MTR9DQpwbG90X2Jhcl9jb2RvbnNfYmVuY2htYXJrKA0KICAuc3BlY2llcyA9ICJIb21vIHNhcGllbnMiLA0KICAuc2F2ZSA9IEZBTFNFDQopDQpgYGANCg0KDQojIyBKYWNjYXJkIGluZGV4DQoNCmBgYHtyfQ0KIyBmdW5jdGlvbiB0byBjb21wdXRlIEphY2NhcmQgaW5kZXggZm9yIHR3byBpbnB1dCBzZXF1ZW5jZXMNCmphY2NhcmRfc2ltaWxhcml0eSA8LSBmdW5jdGlvbihkZikgew0KICBBIDwtIGRmJGNvZG9uczEgfD4gdW5saXN0KCkNCiAgQiA8LSBkZiRjb2RvbnMyIHw+IHVubGlzdCgpDQogIGlmIChsZW5ndGgoQSkgIT0gbGVuZ3RoKEIpKSB7DQogICAgcmV0dXJuKCJkaWZmZXJlbnQgbGVuZ3RoIikNCiAgfSBlbHNlIGlmIChpcy5udWxsKEEpIHwgaXMubnVsbChCKSkgew0KICAgIHJldHVybihOQSkNCiAgfSBlbHNlIHsNCiAgICBpbnRlcnNlY3Rpb24gPC0gbGVuZ3RoKGJhc2U6OmludGVyc2VjdChBLCBCKSkNCiAgICB1bmlvbiA8LSBsZW5ndGgoYmFzZTo6dW5pb24oQSwgQikpDQogICAgcmV0dXJuKGludGVyc2VjdGlvbiAvIHVuaW9uKQ0KICB9DQp9DQoNCmJlbmNobWFya19qYWNjYXJkX3Jlc3VsdHMgPC0gYmVuY2htYXJrX3NlcXVlbmNlX2RhdGEgfD4NCiAgbXV0YXRlKGNvZG9ucyA9IHNlcXVlbmNlIHw+DQogICAgc3RyX2V4dHJhY3RfYWxsKHBhdHRlcm4gPSAiWzphbHBoYTpdezN9IikpIHw+DQogIG11dGF0ZSgNCiAgICBtb2RlbDEgPSBtb2RlbCwgY29kb25zMSA9IGNvZG9ucywNCiAgICBtb2RlbDIgPSBtb2RlbCwgY29kb25zMiA9IGNvZG9ucw0KICApIHw+DQogIG5lc3QoDQogICAgY29tcGFyaXNvbjEgPSBjKG1vZGVsMSwgY29kb25zMSksDQogICAgY29tcGFyaXNvbjIgPSBjKG1vZGVsMiwgY29kb25zMikNCiAgKSB8Pg0KICBzZWxlY3Qob3JnYW5pc20sIHByb3RlaW4sIGNvbXBhcmlzb24xLCBjb21wYXJpc29uMikgfD4NCiAgZ3JvdXBfYnkob3JnYW5pc20sIHByb3RlaW4pIHw+DQogIGNvbXBsZXRlKGNvbXBhcmlzb24xLCBjb21wYXJpc29uMikgfD4NCiAgdW5uZXN0KGMoY29tcGFyaXNvbjEsIGNvbXBhcmlzb24yKSkgfD4NCiAgbmVzdChqYWNjYXJkX2RhdGEgPSBjKGNvZG9uczEsIGNvZG9uczIpKSB8Pg0KICBtdXRhdGUodmFsdWUgPSBtYXAoamFjY2FyZF9kYXRhLCBqYWNjYXJkX3NpbWlsYXJpdHkpKQ0KYGBgDQoNCiMjIyMgRWMNCg0KYGBge3IgZmlnLmhlaWdodD02LCBmaWcud2lkdGg9OH0NCnBsb3QgPC0gcGxvdF90aWxlX3NpbWlsYXJpdHkoDQogIC5kYXRhX3N0cnVjID0gYmVuY2htYXJrX2phY2NhcmRfcmVzdWx0cywNCiAgLm9yZ2FuaXNtID0gb3JnYW5pc21fbGV2ZWxzWzFdLA0KICAubWV0cmljID0gIkphY2NhcmQgaW5kZXgiDQopICsNCiAgc2NhbGVfZmlsbF9kaXN0aWxsZXIoDQogICAgcGFsZXR0ZSA9ICJHcmVlbnMiLA0KICAgIGRpcmVjdGlvbiA9IDENCiAgKQ0KcGxvdA0KIyBnZ3NhdmUocGxvdCwNCiMgICBmaWxlbmFtZSA9ICJqYWNjYXJkX2Vjb2xpLnN2ZyIsDQojICAgaGVpZ2h0ID0gc2ltaWxhcml0eV9wbG90X2hlaWdodCwNCiMgICB3aWR0aCA9IHNpbWlsYXJpdHlfcGxvdF93aWR0aA0KIyApDQpgYGANCg0KIyMjIyBTYw0KDQpgYGB7ciBmaWcuaGVpZ2h0PTYsIGZpZy53aWR0aD04fQ0KcGxvdCA8LSBwbG90X3RpbGVfc2ltaWxhcml0eSgNCiAgLmRhdGFfc3RydWMgPSBiZW5jaG1hcmtfamFjY2FyZF9yZXN1bHRzLA0KICAub3JnYW5pc20gPSBvcmdhbmlzbV9sZXZlbHNbMl0sDQogIC5tZXRyaWMgPSAiSmFjY2FyZCBpbmRleCINCikgKw0KICBzY2FsZV9maWxsX2Rpc3RpbGxlcigNCiAgICBwYWxldHRlID0gIkdyZWVucyIsDQogICAgZGlyZWN0aW9uID0gMQ0KICApDQpwbG90DQojIGdnc2F2ZShwbG90LA0KIyAgIGZpbGVuYW1lID0gImphY2NhcmRfc2NlcmV2aXNpYWUuc3ZnIiwNCiMgICBoZWlnaHQgPSBzaW1pbGFyaXR5X3Bsb3RfaGVpZ2h0LA0KIyAgIHdpZHRoID0gc2ltaWxhcml0eV9wbG90X3dpZHRoDQojICkNCmBgYA0KDQojIyMjIEF0DQoNCmBgYHtyIGZpZy5oZWlnaHQ9NiwgZmlnLndpZHRoPTh9DQpwbG90IDwtIHBsb3RfdGlsZV9zaW1pbGFyaXR5KA0KICAuZGF0YV9zdHJ1YyA9IGJlbmNobWFya19qYWNjYXJkX3Jlc3VsdHMsDQogIC5vcmdhbmlzbSA9IG9yZ2FuaXNtX2xldmVsc1szXSwNCiAgLm1ldHJpYyA9ICJKYWNjYXJkIGluZGV4Ig0KKSArDQogIHNjYWxlX2ZpbGxfZGlzdGlsbGVyKA0KICAgIHBhbGV0dGUgPSAiR3JlZW5zIiwNCiAgICBkaXJlY3Rpb24gPSAxDQogICkNCnBsb3QNCiMgZ2dzYXZlKHBsb3QsDQojICAgZmlsZW5hbWUgPSAiamFjY2FyZF9hdGhhbGlhbmEuc3ZnIiwNCiMgICBoZWlnaHQgPSBzaW1pbGFyaXR5X3Bsb3RfaGVpZ2h0LA0KIyAgIHdpZHRoID0gc2ltaWxhcml0eV9wbG90X3dpZHRoDQojICkNCmBgYA0KDQojIyMjIE1tDQoNCmBgYHtyIGZpZy5oZWlnaHQ9NiwgZmlnLndpZHRoPTh9DQpwbG90IDwtIHBsb3RfdGlsZV9zaW1pbGFyaXR5KA0KICAuZGF0YV9zdHJ1YyA9IGJlbmNobWFya19qYWNjYXJkX3Jlc3VsdHMsDQogIC5vcmdhbmlzbSA9IG9yZ2FuaXNtX2xldmVsc1s0XSwNCiAgLm1ldHJpYyA9ICJKYWNjYXJkIGluZGV4Ig0KKSArDQogIHNjYWxlX2ZpbGxfZGlzdGlsbGVyKA0KICAgIHBhbGV0dGUgPSAiR3JlZW5zIiwNCiAgICBkaXJlY3Rpb24gPSAxDQogICkNCnBsb3QNCiMgZ2dzYXZlKHBsb3QsDQojICAgZmlsZW5hbWUgPSAiamFjY2FyZF9tbXVzY3VsdXMuc3ZnIiwNCiMgICBoZWlnaHQgPSBzaW1pbGFyaXR5X3Bsb3RfaGVpZ2h0LA0KIyAgIHdpZHRoID0gc2ltaWxhcml0eV9wbG90X3dpZHRoDQojICkNCmBgYA0KDQojIyMjIEhzDQoNCmBgYHtyIGZpZy5oZWlnaHQ9NiwgZmlnLndpZHRoPTh9DQpwbG90IDwtIHBsb3RfdGlsZV9zaW1pbGFyaXR5KA0KICAuZGF0YV9zdHJ1YyA9IGJlbmNobWFya19qYWNjYXJkX3Jlc3VsdHMsDQogIC5vcmdhbmlzbSA9IG9yZ2FuaXNtX2xldmVsc1s1XSwNCiAgLm1ldHJpYyA9ICJKYWNjYXJkIGluZGV4Ig0KKSArDQogIHNjYWxlX2ZpbGxfZGlzdGlsbGVyKA0KICAgIHBhbGV0dGUgPSAiR3JlZW5zIiwNCiAgICBkaXJlY3Rpb24gPSAxDQogICkNCnBsb3QNCiMgZ2dzYXZlKHBsb3QsDQojICAgZmlsZW5hbWUgPSAiamFjY2FyZF9oc2FwaWVucy5zdmciLA0KIyAgIGhlaWdodCA9IHNpbWlsYXJpdHlfcGxvdF9oZWlnaHQsDQojICAgd2lkdGggPSBzaW1pbGFyaXR5X3Bsb3Rfd2lkdGgNCiMgKQ0KYGBgDQoNCiMjIyBhbGwNCg0KYGBge3IgZmlnLmhlaWdodD02LCBmaWcud2lkdGg9OH0NCnBsb3QgPC0gcGxvdF90aWxlX3NpbWlsYXJpdHkoDQogIC5kYXRhX3N0cnVjID0gYmVuY2htYXJrX2phY2NhcmRfcmVzdWx0cywNCiAgLm9yZ2FuaXNtID0gIi4qIiwNCiAgLm1ldHJpYyA9ICJKYWNjYXJkIGluZGV4Ig0KKSArDQogIHNjYWxlX2ZpbGxfZGlzdGlsbGVyKA0KICAgIHBhbGV0dGUgPSAiR3JlZW5zIiwNCiAgICBkaXJlY3Rpb24gPSAxDQogICkNCnBsb3QNCiMgZ2dzYXZlKHBsb3QsDQojICAgZmlsZW5hbWUgPSAiamFjY2FyZF9hbGxfc3BlY2llc19tZWFuLnN2ZyIsDQojICAgaGVpZ2h0ID0gc2ltaWxhcml0eV9wbG90X2hlaWdodCwNCiMgICB3aWR0aCA9IHNpbWlsYXJpdHlfcGxvdF93aWR0aA0KIyApDQpgYGANCg0KYGBge3IsIGV2YWw9RkFMU0V9DQpiZW5jaG1hcmtfamFjY2FyZF9yZXN1bHRzIHw+DQogIG11dGF0ZSgNCiAgICBtb2RlbDEgPSBtb2RlbDEgfD4gc3RyX3JlbW92ZV9hbGwoIlxuIiksDQogICAgbW9kZWwyID0gbW9kZWwyIHw+IHN0cl9yZW1vdmVfYWxsKCJcbiIpDQogICkgfD4NCiAgbXV0YXRlKGphY2NhcmQgPSB1bmxpc3QodmFsdWUpKSB8Pg0KICBzZWxlY3QoLWphY2NhcmRfZGF0YSwgdmFsdWUpIHw+DQogIHdyaXRlX3RzdigiYmVuY2htYXJrX2phY2NhcmRfdmFsdWVzLnRzdiIpDQpgYGANCg0KIyMgU2VxIHNpbWlsYXJpdHkNCg0KYGBge3J9DQpzZXF1ZW5jZV9zaW1pbGFyaXR5IDwtIGZ1bmN0aW9uKGRmKSB7DQogIEEgPC0gZGYkY29kb25zMSB8PiB1bmxpc3QoKQ0KICBCIDwtIGRmJGNvZG9uczIgfD4gdW5saXN0KCkNCiAgaWYgKGxlbmd0aChBKSAhPSBsZW5ndGgoQikpIHsNCiAgICByZXR1cm4oImRpZmZlcmVudCBsZW5ndGgiKQ0KICB9IGVsc2UgaWYgKGlzLm51bGwoQSkgfCBpcy5udWxsKEIpKSB7DQogICAgcmV0dXJuKE5BKQ0KICB9IGVsc2Ugew0KICAgIHN1bSA8LSAwDQogICAgZm9yIChpIGluIHNlcSgxLCBsZW5ndGgoQSkpKSB7DQogICAgICBpZiAoQVtpXSA9PSBCW2ldKSB7DQogICAgICAgIHN1bSA8LSBzdW0gKyAxDQogICAgICB9DQogICAgfQ0KICAgIHN1bSA8LSAoc3VtICogMTAwKSAvIGxlbmd0aChBKQ0KICAgIHJldHVybihzdW0pDQogIH0NCn0NCg0KYmVuY2htYXJrX3NlcXVlbmNlX3NpbWlsYXJpdHlfcmVzdWx0cyA8LSBiZW5jaG1hcmtfc2VxdWVuY2VfZGF0YSB8Pg0KICBtdXRhdGUoY29kb25zID0gc2VxdWVuY2UgfD4NCiAgICBzdHJfZXh0cmFjdF9hbGwocGF0dGVybiA9ICJbOmFscGhhOl17M30iKSkgfD4NCiAgbXV0YXRlKA0KICAgIG1vZGVsMSA9IG1vZGVsLCBjb2RvbnMxID0gY29kb25zLA0KICAgIG1vZGVsMiA9IG1vZGVsLCBjb2RvbnMyID0gY29kb25zDQogICkgfD4NCiAgbmVzdCgNCiAgICBjb21wYXJpc29uMSA9IGMobW9kZWwxLCBjb2RvbnMxKSwNCiAgICBjb21wYXJpc29uMiA9IGMobW9kZWwyLCBjb2RvbnMyKQ0KICApIHw+DQogIHNlbGVjdChvcmdhbmlzbSwgcHJvdGVpbiwgY29tcGFyaXNvbjEsIGNvbXBhcmlzb24yKSB8Pg0KICBncm91cF9ieShvcmdhbmlzbSwgcHJvdGVpbikgfD4NCiAgY29tcGxldGUoY29tcGFyaXNvbjEsIGNvbXBhcmlzb24yKSB8Pg0KICB1bm5lc3QoYyhjb21wYXJpc29uMSwgY29tcGFyaXNvbjIpKSB8Pg0KICBuZXN0KHNlcV9zaW1pbGFyaXR5X2RhdGEgPSBjKGNvZG9uczEsIGNvZG9uczIpKSB8Pg0KICBtdXRhdGUodmFsdWUgPSBtYXAoc2VxX3NpbWlsYXJpdHlfZGF0YSwgc2VxdWVuY2Vfc2ltaWxhcml0eSkpDQpgYGANCg0KIyMjIyBFYw0KDQpgYGB7ciBmaWcuaGVpZ2h0PTYsIGZpZy53aWR0aD04fQ0KcGxvdCA8LSBwbG90X3RpbGVfc2ltaWxhcml0eSgNCiAgLmRhdGFfc3RydWMgPSBiZW5jaG1hcmtfc2VxdWVuY2Vfc2ltaWxhcml0eV9yZXN1bHRzLA0KICAub3JnYW5pc20gPSBvcmdhbmlzbV9sZXZlbHNbMV0sDQogIC5tZXRyaWMgPSAic2VxdWVuY2Ugc2ltaWxhcml0eSINCikgKw0KICBzY2FsZV9maWxsX2Rpc3RpbGxlcigNCiAgICBwYWxldHRlID0gIkJsdWVzIiwNCiAgICBkaXJlY3Rpb24gPSAxDQogICkNCnBsb3QNCiMgZ2dzYXZlKHBsb3QsDQojICAgZmlsZW5hbWUgPSAic2VxX3NpbWlsYXJpdHlfZWNvbGkuc3ZnIiwNCiMgICBoZWlnaHQgPSBzaW1pbGFyaXR5X3Bsb3RfaGVpZ2h0LA0KIyAgIHdpZHRoID0gc2ltaWxhcml0eV9wbG90X3dpZHRoDQojICkNCmBgYA0KIyMjIyBTYw0KDQpgYGB7ciBmaWcuaGVpZ2h0PTYsIGZpZy53aWR0aD04fQ0KcGxvdCA8LSBwbG90X3RpbGVfc2ltaWxhcml0eSgNCiAgLmRhdGFfc3RydWMgPSBiZW5jaG1hcmtfc2VxdWVuY2Vfc2ltaWxhcml0eV9yZXN1bHRzLA0KICAub3JnYW5pc20gPSBvcmdhbmlzbV9sZXZlbHNbMl0sDQogIC5tZXRyaWMgPSAic2VxdWVuY2Ugc2ltaWxhcml0eSINCikgKw0KICBzY2FsZV9maWxsX2Rpc3RpbGxlcigNCiAgICBwYWxldHRlID0gIkJsdWVzIiwNCiAgICBkaXJlY3Rpb24gPSAxDQogICkNCnBsb3QNCiMgZ2dzYXZlKHBsb3QsDQojICAgZmlsZW5hbWUgPSAic2VxX3NpbWlsYXJpdHlfc2NlcmV2aXNpYWUuc3ZnIiwNCiMgICBoZWlnaHQgPSBzaW1pbGFyaXR5X3Bsb3RfaGVpZ2h0LA0KIyAgIHdpZHRoID0gc2ltaWxhcml0eV9wbG90X3dpZHRoDQojICkNCmBgYA0KIyMjIyBBdA0KDQpgYGB7ciBmaWcuaGVpZ2h0PTYsIGZpZy53aWR0aD04fQ0KcGxvdCA8LSBwbG90X3RpbGVfc2ltaWxhcml0eSgNCiAgLmRhdGFfc3RydWMgPSBiZW5jaG1hcmtfc2VxdWVuY2Vfc2ltaWxhcml0eV9yZXN1bHRzLA0KICAub3JnYW5pc20gPSBvcmdhbmlzbV9sZXZlbHNbM10sDQogIC5tZXRyaWMgPSAic2VxdWVuY2Ugc2ltaWxhcml0eSINCikgKw0KICBzY2FsZV9maWxsX2Rpc3RpbGxlcigNCiAgICBwYWxldHRlID0gIkJsdWVzIiwNCiAgICBkaXJlY3Rpb24gPSAxDQogICkNCnBsb3QNCiMgZ2dzYXZlKHBsb3QsDQojICAgZmlsZW5hbWUgPSAic2VxX3NpbWlsYXJpdHlfQXRoYWxpYW5hLnN2ZyIsDQojICAgaGVpZ2h0ID0gc2ltaWxhcml0eV9wbG90X2hlaWdodCwNCiMgICB3aWR0aCA9IHNpbWlsYXJpdHlfcGxvdF93aWR0aA0KIyApDQpgYGANCiMjIyMgTW0NCg0KYGBge3IgZmlnLmhlaWdodD02LCBmaWcud2lkdGg9OH0NCnBsb3QgPC0gcGxvdF90aWxlX3NpbWlsYXJpdHkoDQogIC5kYXRhX3N0cnVjID0gYmVuY2htYXJrX3NlcXVlbmNlX3NpbWlsYXJpdHlfcmVzdWx0cywNCiAgLm9yZ2FuaXNtID0gb3JnYW5pc21fbGV2ZWxzWzRdLA0KICAubWV0cmljID0gInNlcXVlbmNlIHNpbWlsYXJpdHkiDQopICsNCiAgc2NhbGVfZmlsbF9kaXN0aWxsZXIoDQogICAgcGFsZXR0ZSA9ICJCbHVlcyIsDQogICAgZGlyZWN0aW9uID0gMQ0KICApDQpwbG90DQojIGdnc2F2ZShwbG90LA0KIyAgIGZpbGVuYW1lID0gInNlcV9zaW1pbGFyaXR5X21tdXNjdWx1cy5zdmciLA0KIyAgIGhlaWdodCA9IHNpbWlsYXJpdHlfcGxvdF9oZWlnaHQsDQojICAgd2lkdGggPSBzaW1pbGFyaXR5X3Bsb3Rfd2lkdGgNCiMgKQ0KYGBgDQojIyMjIEhzDQoNCmBgYHtyIGZpZy5oZWlnaHQ9NiwgZmlnLndpZHRoPTh9DQpwbG90IDwtIHBsb3RfdGlsZV9zaW1pbGFyaXR5KA0KICAuZGF0YV9zdHJ1YyA9IGJlbmNobWFya19zZXF1ZW5jZV9zaW1pbGFyaXR5X3Jlc3VsdHMsDQogIC5vcmdhbmlzbSA9IG9yZ2FuaXNtX2xldmVsc1s1XSwNCiAgLm1ldHJpYyA9ICJzZXF1ZW5jZSBzaW1pbGFyaXR5Ig0KKSArDQogIHNjYWxlX2ZpbGxfZGlzdGlsbGVyKA0KICAgIHBhbGV0dGUgPSAiQmx1ZXMiLA0KICAgIGRpcmVjdGlvbiA9IDENCiAgKQ0KcGxvdA0KIyBnZ3NhdmUocGxvdCwNCiMgICBmaWxlbmFtZSA9ICJzZXFfc2ltaWxhcml0eV9oc2FwaWVucy5zdmciLA0KIyAgIGhlaWdodCA9IHNpbWlsYXJpdHlfcGxvdF9oZWlnaHQsDQojICAgd2lkdGggPSBzaW1pbGFyaXR5X3Bsb3Rfd2lkdGgNCiMgKQ0KYGBgDQojIyMgYWxsDQoNCmBgYHtyIGZpZy5oZWlnaHQ9NiwgZmlnLndpZHRoPTh9DQpwbG90IDwtIHBsb3RfdGlsZV9zaW1pbGFyaXR5KA0KICAuZGF0YV9zdHJ1YyA9IGJlbmNobWFya19zZXF1ZW5jZV9zaW1pbGFyaXR5X3Jlc3VsdHMsDQogIC5vcmdhbmlzbSA9ICIuKiIsDQogIC5tZXRyaWMgPSAic2VxdWVuY2Ugc2ltaWxhcml0eSINCikgKw0KICBzY2FsZV9maWxsX2Rpc3RpbGxlcigNCiAgICBwYWxldHRlID0gIkJsdWVzIiwNCiAgICBkaXJlY3Rpb24gPSAxDQogICkNCnBsb3QNCiMgZ2dzYXZlKHBsb3QsDQojICAgZmlsZW5hbWUgPSAic2VxX3NpbWlsYXJpdHlfYWxsX3NwZWNpZXNfbWVhbi5zdmciLA0KIyAgIGhlaWdodCA9IHNpbWlsYXJpdHlfcGxvdF9oZWlnaHQsDQojICAgd2lkdGggPSBzaW1pbGFyaXR5X3Bsb3Rfd2lkdGgNCiMgKQ0KYGBgDQoNCmBgYHtyLCBldmFsPUZBTFNFfQ0KYmVuY2htYXJrX3NlcXVlbmNlX3NpbWlsYXJpdHlfcmVzdWx0cyB8Pg0KICBtdXRhdGUoDQogICAgbW9kZWwxID0gbW9kZWwxIHw+IHN0cl9yZW1vdmVfYWxsKCJcbiIpLA0KICAgIG1vZGVsMiA9IG1vZGVsMiB8PiBzdHJfcmVtb3ZlX2FsbCgiXG4iKQ0KICApIHw+DQogIG11dGF0ZShzZXFfc2ltaWxhcml0eSA9IHVubGlzdCh2YWx1ZSkpIHw+DQogIHNlbGVjdCgtc2VxX3NpbWlsYXJpdHlfZGF0YSwgLXZhbHVlKSB8Pg0KICB3cml0ZV90c3YoImJlbmNobWFya19zZXF1ZW5jZV9zaW1pbGFyaXR5LnRzdiIpDQpgYGANCg0KIyMgJW1pbm1heCANCg0KYGBge3J9DQpiZW5jaG1hcmtfbWlubWF4X3Jlc3VsdHMgPC0gYmVuY2htYXJrX3NlcXVlbmNlX2RhdGEgfD4NCiAgc2VsZWN0KG9yZ2FuaXNtLCBwcm90ZWluLCBtb2RlbCwgc2VxdWVuY2UpIHw+DQogIG11dGF0ZShzcGVjaWVzID0gaWZlbHNlKHN0cl9kZXRlY3Qob3JnYW5pc20sICJjb2xpIiksICJlY29saSIsDQogICAgaWZlbHNlKHN0cl9kZXRlY3Qob3JnYW5pc20sICJjZXJldmlzaWFlIiksICJzYWNjaGFyb215Y2VzIiwNCiAgICAgIGlmZWxzZShzdHJfZGV0ZWN0KG9yZ2FuaXNtLCAidGhhbGlhbmEiKSwgImFyYWJpZG9wc2lzIiwNCiAgICAgICAgaWZlbHNlKHN0cl9kZXRlY3Qob3JnYW5pc20sICJtdXNjdWx1cyIpLCAibW91c2UiLA0KICAgICAgICAgIGlmZWxzZShzdHJfZGV0ZWN0KG9yZ2FuaXNtLCAic2FwaWVucyIpLCAiaHVtYW4iLCBOQSkNCiAgICAgICAgKQ0KICAgICAgKQ0KICAgICkNCiAgKSkgfD4NCiAgbmVzdChkYXRhID0gYyhzZXF1ZW5jZSwgc3BlY2llcykpIHw+DQogIG11dGF0ZShtaW5tYXggPSBtYXAoZGF0YSwgY29tcHV0ZV9taW5tYXgpKSB8Pg0KICB1bm5lc3QoZGF0YSkgfD4NCiAgdW5uZXN0KG1pbm1heCkgfD4NCiAgZ3JvdXBfYnkob3JnYW5pc20sIG1vZGVsLCBwcm90ZWluKSB8Pg0KICBtdXRhdGUoaW5kZXggPSByb3dfbnVtYmVyKCkpDQpgYGANCg0KIyMjIHBsb3Qgc3BlY2llcw0KDQpgYGB7cn0NCnBsb3QgPC0gcGxvdF9saW5lX21pbm1heCgNCiAgLmRhdGFfc3RydWN0dXJlID0gYmVuY2htYXJrX21pbm1heF9yZXN1bHRzLA0KICAub3JnYW5pc20gPSBvcmdhbmlzbV9sZXZlbHNbMV0sDQogIC5saW1pdHMgPSBjKC0xLCAxLjIpLA0KICAubmNvbCA9IDYNCikNCg0KIyBnZ3NhdmUocGxvdCwNCiMgICBmaWxlbmFtZSA9ICJtaW5tYXhfYmVuY2htYXJrX0Vjb2xpX3c9MTguc3ZnIiwNCiMgICBoZWlnaHQgPSAxMjAsDQojICAgd2lkdGggPSAyMCwNCiMgICBsaW1pdHNpemUgPSBGQUxTRQ0KIyApDQpgYGANCg0KYGBge3J9DQpwbG90IDwtIHBsb3RfbGluZV9taW5tYXgoDQogIC5kYXRhX3N0cnVjdHVyZSA9IGJlbmNobWFya19taW5tYXhfcmVzdWx0cywNCiAgLm9yZ2FuaXNtID0gb3JnYW5pc21fbGV2ZWxzWzJdLA0KICAubGltaXRzID0gYygtMSwgMS4yKSwNCiAgLm5jb2wgPSA1DQopDQoNCiMgZ2dzYXZlKHBsb3QsDQojICAgZmlsZW5hbWUgPSAibWlubWF4X2JlbmNobWFya19TY2VyZXZpc2lhZV93PTE4LnN2ZyIsDQojICAgaGVpZ2h0ID0gMTIwLA0KIyAgIHdpZHRoID0gMTYsDQojICAgbGltaXRzaXplID0gRkFMU0UNCiMgKQ0KYGBgDQoNCmBgYHtyfQ0KcGxvdCA8LSBwbG90X2xpbmVfbWlubWF4KA0KICAuZGF0YV9zdHJ1Y3R1cmUgPSBiZW5jaG1hcmtfbWlubWF4X3Jlc3VsdHMsDQogIC5vcmdhbmlzbSA9IG9yZ2FuaXNtX2xldmVsc1szXSwNCiAgLmxpbWl0cyA9IGMoLTEsIDEuMiksDQogIC5uY29sID0gNQ0KKQ0KDQojIGdnc2F2ZShwbG90LA0KIyAgIGZpbGVuYW1lID0gIm1pbm1heF9iZW5jaG1hcmtfQXRoYWxpYW5hX3c9MTguc3ZnIiwNCiMgICBoZWlnaHQgPSAxMjAsDQojICAgd2lkdGggPSAxNiwNCiMgICBsaW1pdHNpemUgPSBGQUxTRQ0KIyApDQpgYGANCg0KYGBge3J9DQpwbG90IDwtIHBsb3RfbGluZV9taW5tYXgoDQogIC5kYXRhX3N0cnVjdHVyZSA9IGJlbmNobWFya19taW5tYXhfcmVzdWx0cywNCiAgLm9yZ2FuaXNtID0gb3JnYW5pc21fbGV2ZWxzWzRdLA0KICAubGltaXRzID0gYygtMSwgMS4yKSwNCiAgLm5jb2wgPSA1DQopDQoNCiMgZ2dzYXZlKHBsb3QsDQojICAgZmlsZW5hbWUgPSAibWlubWF4X2JlbmNobWFya19NbXVzY3VsdXNfdz0xOC5zdmciLA0KIyAgIGhlaWdodCA9IDEyMCwNCiMgICB3aWR0aCA9IDE2LA0KIyAgIGxpbWl0c2l6ZSA9IEZBTFNFDQojICkNCmBgYA0KDQpgYGB7cn0NCnBsb3QgPC0gcGxvdF9saW5lX21pbm1heCgNCiAgLmRhdGFfc3RydWN0dXJlID0gYmVuY2htYXJrX21pbm1heF9yZXN1bHRzLA0KICAub3JnYW5pc20gPSBvcmdhbmlzbV9sZXZlbHNbNV0sDQogIC5saW1pdHMgPSBjKC0xLCAxLjIpLA0KICAubmNvbCA9IDUNCikNCg0KIyBnZ3NhdmUocGxvdCwNCiMgICBmaWxlbmFtZSA9ICJtaW5tYXhfYmVuY2htYXJrX0hzYXBpZW5zX3c9MTguc3ZnIiwNCiMgICBoZWlnaHQgPSAxMjAsDQojICAgd2lkdGggPSAxNiwNCiMgICBsaW1pdHNpemUgPSBGQUxTRQ0KIyApDQpgYGANCg0KYGBge3IsIGV2YWw9RkFMU0V9DQpiZW5jaG1hcmtfbWlubWF4X3Jlc3VsdHMgfD4NCiAgc2VsZWN0KC1zcGVjaWVzLCAtc2VxdWVuY2UpIHw+DQogIG11dGF0ZSgNCiAgICBtb2RlbCA9IG1vZGVsIHw+IHN0cl9yZW1vdmVfYWxsKCJcbiIpLA0KICAgIG1pbm1heCA9IG1pbm1heCB8PiByb3VuZChkaWdpdHMgPSAyKQ0KICApIHw+DQogIHBpdm90X3dpZGVyKG5hbWVzX2Zyb20gPSBpbmRleCwgdmFsdWVzX2Zyb20gPSBtaW5tYXgpIHw+DQogIHVuaXRlKGNvbCA9ICJtaW5tYXgiLCBtYXRjaGVzKCJcXGQiKSwgc2VwID0gIiwiKSB8Pg0KICBtdXRhdGUobWlubWF4ID0gbWlubWF4IHw+IHN0cl9yZW1vdmVfYWxsKCIsTkEiKSkgfD4NCiAgd3JpdGVfdHN2KCJiZW5jaG1hcmtfbWlubWF4X3Byb2ZpbGVzLnRzdiIpDQpgYGANCg0KIyMgRFRXDQoNCmBgYHtyLCBldmFsPUZBTFNFfQ0KYmVuY2htYXJrX0RUV19yZXN1bHRzIDwtIGJlbmNobWFya19taW5tYXhfcmVzdWx0cyB8Pg0KICBzZWxlY3QoLXNwZWNpZXMsIC1pbmRleCwgLXNlcXVlbmNlKSB8Pg0KICBncm91cF9ieShvcmdhbmlzbSwgcHJvdGVpbiwgbW9kZWwpIHw+DQogIG5lc3QobWlubWF4ID0gbWlubWF4KSB8Pg0KICBtdXRhdGUoDQogICAgbW9kZWwxID0gbW9kZWwsIG1pbm1heDEgPSBtaW5tYXgsDQogICAgbW9kZWwyID0gbW9kZWwsIG1pbm1heDIgPSBtaW5tYXgNCiAgKSB8Pg0KICBuZXN0KA0KICAgIGNvbXBhcmlzb24xID0gYyhtb2RlbDEsIG1pbm1heDEpLA0KICAgIGNvbXBhcmlzb24yID0gYyhtb2RlbDIsIG1pbm1heDIpDQogICkgfD4NCiAgdW5ncm91cCgpIHw+DQogIHNlbGVjdChvcmdhbmlzbSwgcHJvdGVpbiwgY29tcGFyaXNvbjEsIGNvbXBhcmlzb24yKSB8Pg0KICBncm91cF9ieShvcmdhbmlzbSwgcHJvdGVpbikgfD4NCiAgY29tcGxldGUoY29tcGFyaXNvbjEsIGNvbXBhcmlzb24yKSB8Pg0KICB1bm5lc3QoYyhjb21wYXJpc29uMSwgY29tcGFyaXNvbjIpKSB8Pg0KICBuZXN0KGR0d19kYXRhID0gYyhtaW5tYXgxLCBtaW5tYXgyKSkgfD4NCiAgbXV0YXRlKGR0d19vYmplY3QgPSBtYXAoZHR3X2RhdGEsIGNvbXB1dGVfRFRXKSkgfD4NCiAgbXV0YXRlKA0KICAgIGRpc3RhbmNlID0gbWFwKGR0d19vYmplY3QsIHBsdWNrKCJkaXN0YW5jZSIpKSwNCiAgICBub3JtYWxpemVkX2Rpc3RhbmNlID0gbWFwKGR0d19vYmplY3QsIHBsdWNrKCJub3JtYWxpemVkRGlzdGFuY2UiKSkNCiAgKSB8Pg0KICBzZWxlY3Qob3JnYW5pc20sIHByb3RlaW4sIG1vZGVsMSwgbW9kZWwyLCBkaXN0YW5jZSwgbm9ybWFsaXplZF9kaXN0YW5jZSkgfD4NCiAgc2VsZWN0KG9yZ2FuaXNtLCBkZXNjcmlwdGlvbiwgbW9kZWwxLCBtb2RlbDIsIGR0d19vYmplY3QpDQoNCnNhdmVSRFMoYmVuY2htYXJrX0RUV19yZXN1bHRzLCAiYmVuY2htYXJrX0RUV19yZXN1bHRzLnJkcyIpDQpgYGANCg0KYGBge3J9DQpiZW5jaG1hcmtfRFRXX3Jlc3VsdHMgPC0gcmVhZFJEUygiYmVuY2htYXJrX0RUV19yZXN1bHRzLnJkcyIpDQpgYGANCg0KIyMjIyBFYw0KDQpgYGB7ciBmaWcuaGVpZ2h0PTYsIGZpZy53aWR0aD04fQ0KcGxvdCA8LSBwbG90X3RpbGVfc2ltaWxhcml0eSgNCiAgLmRhdGFfc3RydWMgPSBiZW5jaG1hcmtfRFRXX3Jlc3VsdHMgfD4NCiAgICBtdXRhdGUodmFsdWUgPSBub3JtYWxpemVkX2Rpc3RhbmNlKSwNCiAgLm9yZ2FuaXNtID0gb3JnYW5pc21fbGV2ZWxzWzFdLA0KICAubWV0cmljID0gIkRUVyBkaXN0YW5jZSINCikgKw0KICBzY2FsZV9maWxsX2dyYWRpZW50KA0KICAgIGxvdyA9ICIjRjdGRUFEIiwNCiAgICBoaWdoID0gIiMwMTcwOEIiDQogICkNCg0KcGxvdA0KIyBnZ3NhdmUocGxvdCwNCiMgICBmaWxlbmFtZSA9ICJEVFdfZWNvbGlfYmVuY2htYXJrLnN2ZyIsDQojICAgaGVpZ2h0ID0gc2ltaWxhcml0eV9wbG90X2hlaWdodCwNCiMgICB3aWR0aCA9IHNpbWlsYXJpdHlfcGxvdF93aWR0aA0KIyApDQpgYGANCg0KIyMjIyBTYw0KDQpgYGB7ciBmaWcuaGVpZ2h0PTYsIGZpZy53aWR0aD04fQ0KcGxvdCA8LSBwbG90X3RpbGVfc2ltaWxhcml0eSgNCiAgLmRhdGFfc3RydWMgPSBiZW5jaG1hcmtfRFRXX3Jlc3VsdHMgfD4gbXV0YXRlKHZhbHVlID0gbm9ybWFsaXplZF9kaXN0YW5jZSksDQogIC5vcmdhbmlzbSA9IG9yZ2FuaXNtX2xldmVsc1syXSwNCiAgLm1ldHJpYyA9ICJEVFcgZGlzdGFuY2UiDQopICsNCiAgc2NhbGVfZmlsbF9ncmFkaWVudCgNCiAgICBsb3cgPSAiI0Y3RkVBRCIsDQogICAgaGlnaCA9ICIjMDE3MDhCIg0KICApDQoNCnBsb3QNCiMgZ2dzYXZlKHBsb3QsDQojICAgZmlsZW5hbWUgPSAiRFRXX3NjZXJldmlzaWFlX2JlbmNobWFyay5zdmciLA0KIyAgIGhlaWdodCA9IHNpbWlsYXJpdHlfcGxvdF9oZWlnaHQsDQojICAgd2lkdGggPSBzaW1pbGFyaXR5X3Bsb3Rfd2lkdGgNCiMgKQ0KYGBgDQoNCiMjIyMgQXQNCg0KYGBge3IgZmlnLmhlaWdodD02LCBmaWcud2lkdGg9OH0NCnBsb3QgPC0gcGxvdF90aWxlX3NpbWlsYXJpdHkoDQogIC5kYXRhX3N0cnVjID0gYmVuY2htYXJrX0RUV19yZXN1bHRzIHw+IG11dGF0ZSh2YWx1ZSA9IG5vcm1hbGl6ZWRfZGlzdGFuY2UpLA0KICAub3JnYW5pc20gPSBvcmdhbmlzbV9sZXZlbHNbM10sDQogIC5tZXRyaWMgPSAiRFRXIGRpc3RhbmNlIg0KKSArDQogIHNjYWxlX2ZpbGxfZ3JhZGllbnQoDQogICAgbG93ID0gIiNGN0ZFQUQiLA0KICAgIGhpZ2ggPSAiIzAxNzA4QiINCiAgKQ0KDQpwbG90DQojIGdnc2F2ZShwbG90LA0KIyAgIGZpbGVuYW1lID0gIkRUV19BdGhhbGlhbmFfYmVuY2htYXJrLnN2ZyIsDQojICAgaGVpZ2h0ID0gc2ltaWxhcml0eV9wbG90X2hlaWdodCwNCiMgICB3aWR0aCA9IHNpbWlsYXJpdHlfcGxvdF93aWR0aA0KIyApDQpgYGANCg0KIyMjIyBNbQ0KDQpgYGB7ciBmaWcuaGVpZ2h0PTYsIGZpZy53aWR0aD04fQ0KcGxvdCA8LSBwbG90X3RpbGVfc2ltaWxhcml0eSgNCiAgLmRhdGFfc3RydWMgPSBiZW5jaG1hcmtfRFRXX3Jlc3VsdHMgfD4gbXV0YXRlKHZhbHVlID0gbm9ybWFsaXplZF9kaXN0YW5jZSksDQogIC5vcmdhbmlzbSA9IG9yZ2FuaXNtX2xldmVsc1s0XSwNCiAgLm1ldHJpYyA9ICJEVFcgZGlzdGFuY2UiDQopICsNCiAgc2NhbGVfZmlsbF9ncmFkaWVudCgNCiAgICBsb3cgPSAiI0Y3RkVBRCIsDQogICAgaGlnaCA9ICIjMDE3MDhCIg0KICApDQoNCnBsb3QNCiMgZ2dzYXZlKHBsb3QsDQojICAgZmlsZW5hbWUgPSAiRFRXX21tdXNjdWx1c19iZW5jaG1hcmsuc3ZnIiwNCiMgICBoZWlnaHQgPSBzaW1pbGFyaXR5X3Bsb3RfaGVpZ2h0LA0KIyAgIHdpZHRoID0gc2ltaWxhcml0eV9wbG90X3dpZHRoDQojICkNCmBgYA0KIyMjIyBIcw0KDQpgYGB7ciBmaWcuaGVpZ2h0PTYsIGZpZy53aWR0aD04fQ0KcGxvdCA8LSBwbG90X3RpbGVfc2ltaWxhcml0eSgNCiAgLmRhdGFfc3RydWMgPSBiZW5jaG1hcmtfRFRXX3Jlc3VsdHMgfD4gbXV0YXRlKHZhbHVlID0gbm9ybWFsaXplZF9kaXN0YW5jZSksDQogIC5vcmdhbmlzbSA9IG9yZ2FuaXNtX2xldmVsc1s1XSwNCiAgLm1ldHJpYyA9ICJEVFcgZGlzdGFuY2UiDQopICsNCiAgc2NhbGVfZmlsbF9ncmFkaWVudCgNCiAgICBsb3cgPSAiI0Y3RkVBRCIsDQogICAgaGlnaCA9ICIjMDE3MDhCIg0KICApDQoNCnBsb3QNCiMgZ2dzYXZlKHBsb3QsDQojICAgZmlsZW5hbWUgPSAiRFRXX2hzYXBpZW5zX2JlbmNobWFyay5zdmciLA0KIyAgIGhlaWdodCA9IHNpbWlsYXJpdHlfcGxvdF9oZWlnaHQsDQojICAgd2lkdGggPSBzaW1pbGFyaXR5X3Bsb3Rfd2lkdGgNCiMgKQ0KYGBgDQojIyMjIGFsbA0KYGBge3IgZmlnLmhlaWdodD02LCBmaWcud2lkdGg9OH0NCnBsb3QgPC0gcGxvdF90aWxlX3NpbWlsYXJpdHkoDQogIC5kYXRhX3N0cnVjID0gYmVuY2htYXJrX0RUV19yZXN1bHRzIHw+IG11dGF0ZSh2YWx1ZSA9IG5vcm1hbGl6ZWRfZGlzdGFuY2UpLA0KICAub3JnYW5pc20gPSAiLioiLA0KICAubWV0cmljID0gIkRUVyBkaXN0YW5jZSINCikgKw0KICBzY2FsZV9maWxsX2dyYWRpZW50KA0KICAgIGxvdyA9ICIjRjdGRUFEIiwNCiAgICBoaWdoID0gIiMwMTcwOEIiDQogICkNCg0KcGxvdA0KIyBnZ3NhdmUocGxvdCwNCiMgICBmaWxlbmFtZSA9ICJEVFdfZGlzdGFuY2VzX2JldHdlZW5fbW9kZWxzX2Zvcl9iZW5jaG1hcmtfZ2VuZXMuc3ZnIiwNCiMgICBoZWlnaHQgPSBzaW1pbGFyaXR5X3Bsb3RfaGVpZ2h0LA0KIyAgIHdpZHRoID0gc2ltaWxhcml0eV9wbG90X3dpZHRoDQojICkNCmBgYA0KDQpgYGB7ciwgZXZhbD1GQUxTRX0NCmJlbmNobWFya19EVFdfcmVzdWx0cyB8Pg0KICBtdXRhdGUoDQogICAgbW9kZWwxID0gbW9kZWwxIHw+IHN0cl9yZW1vdmVfYWxsKCJcbiIpLA0KICAgIG1vZGVsMiA9IG1vZGVsMiB8PiBzdHJfcmVtb3ZlX2FsbCgiXG4iKQ0KICApIHw+DQogIHNlbGVjdChvcmdhbmlzbSwgcHJvdGVpbiwgbW9kZWwxLCBtb2RlbDIsIGRpc3RhbmNlLCBub3JtYWxpemVkX2Rpc3RhbmNlKSB8Pg0KICBtdXRhdGUoDQogICAgZGlzdGFuY2UgPSB1bmxpc3QoZGlzdGFuY2UpLA0KICAgIG5vcm1hbGl6ZWRfZGlzdGFuY2UgPSB1bmxpc3Qobm9ybWFsaXplZF9kaXN0YW5jZSkNCiAgKSB8Pg0KICB3cml0ZV90c3YoImJlbmNobWFya19EVFdfbWlubWF4LnRzdiIpDQpgYGANCg0KIyMgTmVnYXRpdmUgY2lzLWVsZW1lbnRzDQoNCmBgYHtyIGZpZy5oZWlnaHQ9MywgZmlnLndpZHRoPTEwfQ0KcGxvdCA8LSBiZW5jaG1hcmtfc2VxdWVuY2VfZGF0YSB8Pg0KICBtdXRhdGUob3JnYW5pc20gPSBmYWN0b3Iob3JnYW5pc20sIGxldmVscyA9IG9yZ2FuaXNtX2xldmVscykpIHw+DQogIGdncGxvdChhZXMoeCA9IG1vZGVsLCB5ID0gYXMubnVtZXJpYyhuYl9jaXNfZWxlbWVudCksIGNvbG9yID0gbW9kZWwpKSArDQogIGdlb21fYm94cGxvdChjb2xvciA9ICJibGFjayIsIG91dGxpZXIuc2hhcGUgPSBOQSkgKw0KICBnZW9tX2ppdHRlcih3aWR0aCA9IDAuMiwgaGVpZ2h0ID0gMC4yLCBzaXplID0gMSwgYWxwaGEgPSAwLjUpICsNCiAgc2NhbGVfY29sb3JfbWFudWFsKHZhbHVlcyA9IG1vZGVsX2NvbG9yc1stMV0pICsNCiAgc3RhdF9zdW1tYXJ5KA0KICAgIGZ1bi55ID0gbWVhbiwgZnVuLm1pbiA9IG1lYW4sIGZ1bi5tYXggPSBtZWFuLA0KICAgIGdlb20gPSAicG9pbnQiLCBjb2xvciA9ICJibGFjayIsIHNoYXBlID0gNCwgc2l6ZSA9IDQNCiAgKSArDQogIHNjYWxlX3lfY29udGludW91cyhicmVha3MgPSBjKDAsIDIsIDQsIDYsIDgsIDEwKSkgKw0KICBmYWNldF93cmFwKH5vcmdhbmlzbSwgbmNvbCA9IDUsIHNjYWxlcyA9ICJmcmVlIikgKw0KICB5bGFiKCJOdW1iZXIgb2YgY2lzIHJlZ3VsYXRvcnkgZWxlbWVudCIpICsNCiAgdGhlbWVfYncoKSArDQogIHRoZW1lKA0KICAgIGF4aXMudGl0bGUueCA9IGVsZW1lbnRfYmxhbmsoKSwNCiAgICBheGlzLnRleHQueCA9IGVsZW1lbnRfYmxhbmsoKSwNCiAgICBsZWdlbmQudGl0bGUgPSBlbGVtZW50X2JsYW5rKCksDQogICAgcGxvdC50aXRsZSA9IGVsZW1lbnRfdGV4dChoanVzdCA9IDAuNSksDQogICAgc3RyaXAudGV4dCA9IGVsZW1lbnRfdGV4dChmYWNlID0gIml0YWxpYyIpLA0KICAgIGxlZ2VuZC5wb3NpdGlvbiA9ICJub25lIg0KICApDQoNCnBsb3QNCiMgZ2dzYXZlKHBsb3QsDQojICAgZmlsZW5hbWUgPSAiQ2lzX3JlZ3VsYXRvcnlfZWxlbWVudHNfd2l0aG91dF9sZWdlbmQuc3ZnIiwNCiMgICBoZWlnaHQgPSAzLA0KIyAgIHdpZHRoID0gMTANCiMgKQ0KYGBgDQojIyBSTkEgZm9sZA0KDQpgYGB7ciwgZXZhbD1GQUxTRX0NCmJlbmNobWFya19STkFfZm9sZGluZ19yZXN1bHRzIDwtIGJlbmNobWFya19zZXF1ZW5jZV9kYXRhIHw+DQogIG11dGF0ZShSTkFfZm9sZF9vYmplY3QgPSBtYXAoc2VxdWVuY2UsIGNvbXB1dGVfUk5BX2ZvbGQpKSB8Pg0KICBzZWxlY3Qob3JnYW5pc20sIHByb3RlaW4sIG1vZGVsLCBzZXF1ZW5jZSwgUk5BX2ZvbGRfb2JqZWN0KQ0KDQpzYXZlUkRTKGJlbmNobWFya19STkFfZm9sZGluZ19yZXN1bHRzLCAiYmVuY2htYXJrX1JOQV9mb2xkaW5nLnJkcyIpDQpgYGANCg0KYGBge3J9DQpiZW5jaG1hcmtfUk5BX2ZvbGRpbmdfcmVzdWx0cyA8LSByZWFkUkRTKCJiZW5jaG1hcmtfUk5BX2ZvbGRpbmcucmRzIikgfD4NCiAgbXV0YXRlKA0KICAgIG9yZ2FuaXNtID0gZmFjdG9yKG9yZ2FuaXNtLCBsZXZlbHMgPSBvcmdhbmlzbV9sZXZlbHMpLA0KICAgIG1mZSA9IG1hcChSTkFfZm9sZF9vYmplY3QsIHBsdWNrKCJtZmUiKSksDQogICAgbWZlID0gYXMubnVtZXJpYyhtZmUpLA0KICAgIGxlbmd0aCA9IHN0cl9jb3VudChzZXF1ZW5jZSksDQogICAgbWZlX25vcm1hbGl6ZWRfYnlfbGVuZ3RoID0gbWZlIC8gbGVuZ3RoLA0KICAgIEdDX2NvbnRlbnQgPSBzdHJfY291bnQoc2VxdWVuY2UsICJHfEMiKSAvIGxlbmd0aA0KICApDQpgYGANCg0KIyMjIyBtZmUgIyBsZW5ndGgNCg0KYGBge3IgZmlnLmhlaWdodD00LCBmaWcud2lkdGg9Nn0NCnBsb3QgPC0gYmVuY2htYXJrX1JOQV9mb2xkaW5nX3Jlc3VsdHMgfD4NCiAgbXV0YXRlKHlfcGxvdCA9IG1mZSwgeF9wbG90ID0gbGVuZ3RoKSB8Pg0KICBwbG90X3Ntb290aF9tb2RlbCgpICsNCiAgc2NhbGVfY29sb3JfbWFudWFsKHZhbHVlcyA9IG1vZGVsX2NvbG9yc1stMV0pICsNCiAgc2NhbGVfZmlsbF9tYW51YWwodmFsdWVzID0gbW9kZWxfY29sb3JzWy0xXSkNCnBsb3QNCiMgZ2dzYXZlKHBsb3QsDQojICAgZmlsZW5hbWUgPSAiUk5BX2ZvbGRpbmdfMV9saW5lYXJfcmVsYXRpb25fdG9fbGVuZ3RoX2JlbmNobWFyay5zdmciLA0KIyAgIGhlaWdodCA9IDQsDQojICAgd2lkdGggPSA2DQojICkNCmBgYA0KDQojIyMjIG5vcm1hbGl6ZWQgYnkgc3BlY2llcw0KYGBge3IgZmlnLmhlaWdodD00LCBmaWcud2lkdGg9Nn0NCnBsb3QgPC0gYmVuY2htYXJrX1JOQV9mb2xkaW5nX3Jlc3VsdHMgfD4NCiAgbXV0YXRlKHZhbHVlID0gbWZlX25vcm1hbGl6ZWRfYnlfbGVuZ3RoKSB8Pg0KICBwbG90X2ppdHRlcl9zcGVjaWVzKCkgKw0KICB4bGFiKCJtZmUgLyBsZW5ndGgiKQ0KcGxvdA0KIyBnZ3NhdmUocGxvdCwNCiMgICBmaWxlbmFtZSA9ICJSTkFfZm9sZGluZ18zX3NwZWNpZXNfc2VwYXJhdGlvbl9ub3JtYWxpemVkX2JlbmNobWFyay5zdmciLA0KIyAgIGhlaWdodCA9IDQsDQojICAgd2lkdGggPSA2DQojICkNCmBgYA0KDQojIyMjIEdDIGJ5IHNwZWNpZXMNCg0KYGBge3IgZmlnLmhlaWdodD00LCBmaWcud2lkdGg9Nn0NCnBsb3QgPC0gYmVuY2htYXJrX1JOQV9mb2xkaW5nX3Jlc3VsdHMgfD4NCiAgbXV0YXRlKHZhbHVlID0gR0NfY29udGVudCkgfD4NCiAgcGxvdF9qaXR0ZXJfc3BlY2llcygpICsNCiAgeGxhYigiR0MgY29udGVudCIpDQpwbG90DQojIGdnc2F2ZShwbG90LA0KIyAgIGZpbGVuYW1lID0gIlJOQV9mb2xkaW5nXzRfc3BlY2llc19zZXBhcmF0aW9uX0dDX2NvbnRlbnQuc3ZnIiwNCiMgICBoZWlnaHQgPSA0LA0KIyAgIHdpZHRoID0gNg0KIyApDQpgYGANCg0KYGBge3IsIGV2YWw9RkFMU0V9DQpiZW5jaG1hcmtfUk5BX2ZvbGRpbmdfcmVzdWx0cyB8Pg0KICBtdXRhdGUobW9kZWwgPSBtb2RlbCB8PiBzdHJfcmVtb3ZlX2FsbCgiXG4iKSkgfD4NCiAgc2VsZWN0KG9yZ2FuaXNtLCBwcm90ZWluLCBtZmUsIGxlbmd0aCwgbWZlX25vcm1hbGl6ZWRfYnlfbGVuZ3RoLCBHQ19jb250ZW50KSB8Pg0KICB3cml0ZV90c3YoImJlbmNobWFya19STkFfZm9sZC50c3YiKQ0KYGBgDQoNCkdlbm9taWMgYW5kIG1vZGVsIGdlbmVyYXRlZCBzZXF1ZW5jZXMgc2hvdWxkIGJlIHJlY292ZXJlZCBvbiBaZW5vZG8uDQoNCiMgZ2Vub21lIHNlcXVlbmNlcw0KDQpgYGB7ciwgZXZhbD1GQUxTRX0NCiMgaW1wb3J0IG5hdHVyYWwgYW5kIG9wdGltaXplZCBzZXF1ZW5jZXMNCmNvZG9uX2Fjcm9zc19nZW5vbWVzIDwtIGdlbm9taWNfc2VxdWVuY2VzIHw+IA0KICBtdXRhdGUoIk5hdHVyYWwiPXN0cl9leHRyYWN0X2FsbChvcmlnaW5hbF9zZXF1ZW5jZSwgIltBLVpdezN9IiksDQogICAgICAgICAiQmFzZSBcbiBtb2RlbCI9c3RyX2V4dHJhY3RfYWxsKGJhc2VfbW9kZWwsICJbQS1aXXszfSIpLA0KICAgICAgICAgIkZpbmUgXG4gdHVuZWQiPXN0cl9leHRyYWN0X2FsbChmaW5lX3R1bmVkLCAiW0EtWl17M30iKSkgfD4gDQogIHBpdm90X2xvbmdlcihjb2xzPWMoIk5hdHVyYWwiLCAiQmFzZSBcbiBtb2RlbCIsICJGaW5lIFxuIHR1bmVkIiksDQogICAgICAgICAgICAgICBuYW1lc190bz0ibW9kZWwiLA0KICAgICAgICAgICAgICAgdmFsdWVzX3RvID0gImNvZG9uIikgfD4gDQogIHVubmVzdF9sb25nZXIoY29kb24pIHw+IA0KICBpbm5lcl9qb2luKGNvZG9uX2FhX3RhYmxlLA0KICAgICAgICAgICAgIGJ5PWMoImNvZG9uIj0iY29kb24iKSkgfD4gDQogIG11dGF0ZShtb2RlbD1mYWN0b3IobW9kZWwsDQogICAgICAgICAgICAgICAgICAgICAgbGV2ZWxzPWMoIk5hdHVyYWwiLCAiQmFzZSBcbiBtb2RlbCIsICJGaW5lIFxuIHR1bmVkIikpKQ0Kcm0oZ2Vub21pY19zZXF1ZW5jZXMpDQpgYGANCg0KIyMgY29kb24gZGlzdHJpYnV0aW9uDQoNCmBgYHtyLCBldmFsPUZBTFNFfQ0KY29kb25fYWNyb3NzX2dlbm9tZXMgfD4NCiAgbXV0YXRlKG1vZGVsPW1vZGVsIHw+IHN0cl9yZW1vdmVfYWxsKCJcbiIpKSB8PiANCiAgZ3JvdXBfYnkob3JnYW5pc20sIG1vZGVsLCB0b2tlbikgfD4gDQogIHN1bW1hcmlzZShjb2Rvbl9jb3VudD1uKCkpIHw+IA0KICB3cml0ZV90c3YoImdlbm9tZV9zZXF1ZW5jZXNfY29kb25fY291bnRzLnRzdiIpDQpgYGANCg0KYGBge3IsIGV2YWw9RkFMU0V9DQpwbG90MSA8LSBjb2Rvbl9hY3Jvc3NfZ2Vub21lcyB8PiANCiAgZmlsdGVyKG9yZ2FuaXNtPT0iRXNjaGVyaWNoaWEgY29saSIpICsNCiAgZ2dwbG90KGFlcyh5ID0gdG9rZW4sIGZpbGw9bW9kZWwpKSArDQogICAgZ2VvbV9iYXIoKSArDQogIHNjYWxlX2ZpbGxfbWFudWFsKHZhbHVlcz1tb2RlbF9jb2xvcnNbMTozXSkrDQogICAgICBzY2FsZV94X2NvbnRpbnVvdXMoYnJlYWtzID0gYygwLCAxMDAwMDApKSsNCiAgICBmYWNldF93cmFwKH5tb2RlbCwgbmNvbD0zKSArDQogICAgdGhlbWVfYncoKSArDQogICAgeWxhYigiQ29kb25zIikgKw0KICAgIHhsYWIoIiIpICsNCiAgICBnZ3RpdGxlKCJFLiBjb2xpIikgKw0KICAgIHRoZW1lKHBsb3QudGl0bGUgPSBlbGVtZW50X3RleHQoaGp1c3QgPSAwLjUpLA0KICAgICAgICAgIGF4aXMudGl0bGUueT1lbGVtZW50X3RleHQoZmFjZT0iaXRhbGljIikpDQpwbG90MiA8LSBjb2Rvbl9hY3Jvc3NfZ2Vub21lcyB8PiANCiAgZmlsdGVyKG9yZ2FuaXNtPT0iU2FjY2hhcm9teWNlcyBjZXJldmlzaWFlIikgfD4gDQogIGdncGxvdChhZXMoeSA9IHRva2VuLCBmaWxsPW1vZGVsKSkgKw0KICAgIGdlb21fYmFyKCkgKw0KICBzY2FsZV9maWxsX21hbnVhbCh2YWx1ZXM9bW9kZWxfY29sb3JzWzE6M10pKw0KICAgICAgc2NhbGVfeF9jb250aW51b3VzKGJyZWFrcyA9IGMoMCwgMjAwMDAwKSkrDQogICAgZmFjZXRfd3JhcCh+bW9kZWwsIG5jb2w9MykgKw0KICAgIHRoZW1lX2J3KCkgKw0KICAgIHlsYWIoIkNvZG9ucyIpICsNCiAgICB4bGFiKCIiKSArDQogICAgZ2d0aXRsZSgiUy4gY2VyZXZpc2lhZSIpICsNCiAgICB0aGVtZShwbG90LnRpdGxlID0gZWxlbWVudF90ZXh0KGhqdXN0ID0gMC41KSwNCiAgICAgICAgICBheGlzLnRpdGxlLnk9ZWxlbWVudF90ZXh0KGZhY2U9Iml0YWxpYyIpKQ0KcGxvdDMgPC0gY29kb25fYWNyb3NzX2dlbm9tZXMgfD4gDQogIGZpbHRlcihvcmdhbmlzbT09IkFyYWJpZG9wc2lzIHRoYWxpYW5hIikgfD4gDQogIGdncGxvdChhZXMoeSA9IHRva2VuLCBmaWxsPW1vZGVsKSkgKw0KICAgIGdlb21fYmFyKCkgKw0KICBzY2FsZV9maWxsX21hbnVhbCh2YWx1ZXM9bW9kZWxfY29sb3JzWzE6M10pKw0KICAgICAgc2NhbGVfeF9jb250aW51b3VzKGJyZWFrcyA9IGMoMCwgMTAwMDAwMCkpKw0KICAgIGZhY2V0X3dyYXAofm1vZGVsLCBuY29sPTMpICsNCiAgICB0aGVtZV9idygpICsNCiAgICB5bGFiKCJDb2RvbnMiKSArDQogICAgeGxhYigiIikgKw0KICAgIGdndGl0bGUoIkEuIHRoYWxpYW5hIikgKw0KICAgIHRoZW1lKHBsb3QudGl0bGUgPSBlbGVtZW50X3RleHQoaGp1c3QgPSAwLjUpLA0KICAgICAgICAgIGF4aXMudGl0bGUueT1lbGVtZW50X3RleHQoZmFjZT0iaXRhbGljIikpDQpwbG90NCA8LSBjb2Rvbl9hY3Jvc3NfZ2Vub21lcyB8PiANCiAgZmlsdGVyKG9yZ2FuaXNtPT0iTXVzIG11c2N1bHVzIikgfD4gDQogIGdncGxvdChhZXMoeSA9IHRva2VuLCBmaWxsPW1vZGVsKSkgKw0KICAgIGdlb21fYmFyKCkgKw0KICBzY2FsZV9maWxsX21hbnVhbCh2YWx1ZXM9bW9kZWxfY29sb3JzWzE6M10pKw0KICAgICAgc2NhbGVfeF9jb250aW51b3VzKGJyZWFrcyA9IGMoMCwzMDAwMDAwKSkrDQogICAgZmFjZXRfd3JhcCh+bW9kZWwsIG5jb2w9MykgKw0KICAgIHRoZW1lX2J3KCkgKw0KICAgIHlsYWIoIkNvZG9ucyIpICsNCiAgICB4bGFiKCIiKSArDQogICAgZ2d0aXRsZSgiTS4gbXVzY3VsdXMiKSArDQogICAgdGhlbWUocGxvdC50aXRsZSA9IGVsZW1lbnRfdGV4dChoanVzdCA9IDAuNSksDQogICAgICAgICAgYXhpcy50aXRsZS55PWVsZW1lbnRfdGV4dChmYWNlPSJpdGFsaWMiKSkNCnBsb3Q1IDwtIGNvZG9uX2Fjcm9zc19nZW5vbWVzIHw+IA0KICBmaWx0ZXIob3JnYW5pc209PSJIb21vIHNhcGllbnMiKSB8PiANCiAgZ2dwbG90KGFlcyh5ID0gdG9rZW4sIGZpbGw9bW9kZWwpKSArDQogICAgZ2VvbV9iYXIoKSArDQogIHNjYWxlX2ZpbGxfbWFudWFsKHZhbHVlcz1tb2RlbF9jb2xvcnNbMTozXSkrDQogICAgICBzY2FsZV94X2NvbnRpbnVvdXMoYnJlYWtzID0gYygwLCA1MDAwMDAwKSkrDQogICAgZmFjZXRfd3JhcCh+bW9kZWwsIG5jb2w9MykgKw0KICAgIHRoZW1lX2J3KCkgKw0KICAgIHlsYWIoIkNvZG9ucyIpICsNCiAgICB4bGFiKCIiKSArDQogICAgZ2d0aXRsZSgiSC4gc2FwaWVucyIpICsNCiAgICB0aGVtZShwbG90LnRpdGxlID0gZWxlbWVudF90ZXh0KGhqdXN0ID0gMC41KSwNCiAgICAgICAgICBheGlzLnRpdGxlLnk9ZWxlbWVudF90ZXh0KGZhY2U9Iml0YWxpYyIpKQ0KYGBgDQoNCmBgYHtyLCBldmFsPUZBTFNFfQ0KcGxvdD1wbG90MStwbG90MitwbG90MytwbG90NCtwbG90NSsNCiAgcGxvdF9sYXlvdXQoDQogICAgICBndWlkZXMgPSAiY29sbGVjdCIsDQogICAgICBheGVzID0gImNvbGxlY3QiLA0KICAgICAgbmNvbD01DQogICAgKSArDQogICAgcGxvdF9hbm5vdGF0aW9uKA0KICAgICAgdGl0bGUgPSBwYXN0ZTAoIkNvZG9uIGRpc3RyaWJ1dGlvbiBhY3Jvc3MgZ2Vub21pYyBzZXF1ZW5jZXMiKSwNCiAgICAgIHRoZW1lID0gdGhlbWUocGxvdC50aXRsZSA9IGVsZW1lbnRfdGV4dChoanVzdCA9IDAuNSkpICYNCiAgICAgICAgdGhlbWUocGxvdC50aXRsZSA9IGVsZW1lbnRfdGV4dChzaXplID0gMTgpKQ0KICAgICkNCmBgYA0KDQpgYGB7ciwgZXZhbD1GQUxTRSwgZmlnLmhlaWdodD0xMiwgZmlnLndpZHRoPTE0fQ0KcGxvdA0KYGBgDQoNCiMgU2Vzc2lvbiBpbmZvDQoNCmBgYHtyfQ0KcHJpbnQoc2Vzc2lvbkluZm8oKSwgbG9jYWxlID0gRkFMU0UpDQpgYGANCg0K
